# RpoN1 and RpoN2 play different regulatory roles in virulence traits, flagellar biosynthesis, and basal metabolism in *Xanthomonas campestris*

**DOI:** 10.1111/mpp.12938

**Published:** 2020-04-13

**Authors:** Kaihuai Li, Guichun Wu, Yuling Liao, Quan Zeng, Haihong Wang, Fengquan Liu

**Affiliations:** ^1^ College of Plant Protection Nanjing Agricultural University Nanjing China; ^2^ Institute of Plant Protection Jiangsu Academy of Agricultural Sciences Nanjing China; ^3^ Guangdong Provincial Key Laboratory of Protein Function and Regulation in Agricultural Organisms College of Life Sciences South China Agricultural University Guangzhou China; ^4^ Department of Plant Pathology and Ecology The Connecticut Agricultural Experiment Station New Haven CT USA

**Keywords:** flagellar synthesis, sigma factor 54 RpoN, virulence, *Xanthomonas campestris* pv. *campestris*

## Abstract

Homologous regulatory factors are widely present in bacteria, but whether homologous regulators synergistically or differentially regulate different biological functions remains mostly unknown. Here, we report that the homologous regulators RpoN1 and RpoN2 of the plant pathogen *Xanthomonas campestris* pv. *campestris* (Xcc) play different regulatory roles with respect to virulence traits, flagellar biosynthesis, and basal metabolism. RpoN2 directly regulated Xcc *fliC* and *fliQ* to modulate flagellar synthesis in *X. campestris*, thus affecting the swimming motility of *X. campestris*. Mutation of *rpoN2* resulted in reduced production of biofilms and extracellular polysaccharides in Xcc. These defects may together cause reduced virulence of the *rpoN2* mutant against the host plant. Moreover, we demonstrated that RpoN1 could regulate branched‐chain fatty acid production and modulate the synthesis of diffusible signal factor family quorum sensing signals. Although RpoN1 and RpoN2 are homologues, the regulatory roles and biological functions of these proteins were not interchangeable. Overall, our report provides new insights into the two different molecular roles that form the basis for the transcriptional specialization of RpoN homologues.

## Graphical abstract

RpoN1 and RpoN2 have distinct roles and different regulatory functions in basal metabolism, flagellar biosynthesis, extracellular polysaccharides formation, biofilm formation, and virulence in *Xanthomonas campestris*.

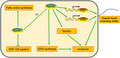

## 1 INTRODUCTION

Homologous regulatory factors are widely present in bacterial genomes. These homologous regulators may display functional redundancy or different biological functions, and some homologues even have no biological function, for example the two homologous ArsR regulators of *Pseudomonas putida* KT2440 bind arsenite with similar affinities (Fernandez *et al.*, 2016). AgaR2 and AgaR1 are two novel regulators that are specific for GalN/GalNAc catabolism and have been assigned distinct roles in bacterial infection (Zhang *et al.*, 2015). NagC and Mlc, homologous members of the ROK (repressors, open reading frames [ORFs] and kinases) family of proteins with almost identical helix‐turn‐helix DNA‐binding motifs, specifically regulate genes for the transport and utilization of *N*‐acetylglucosamine and glucose (Brechemier‐Baey *et al.*, 2015). Two homologous members of the cyclic‐AMP receptor protein (CRP) family of transcription factors (MSMEG_0539 and MSMEG_6189) show differences in cAMP binding affinity, trypsin sensitivity, and binding to a CRP site in *Mycobacterium smegmatis* (Sharma *et al.*, 2014). Three homologous LysR‐type transcriptional regulators (MetR, CysR, and HomR) control sulphur amino acid supply in *Streptococcus mutans* (Sperandio *et al.*, 2010), while *rpoN1* and *rpoN2* of *Xanthomonas citri* subsp. *citri* play different roles in virulence, nutrient utilization, and cell motility (Gicharu *et al.*, 2016). However, some homologous transcription factors still have unknown biological and transcriptional functions. Here, we use *Xanthomonas campestris* pv. *campestris* (Xcc) sigma factor 54 (σ^54^) proteins (XCC RpoN1 and XCC RpoN1) as a model to illustrate the characteristics of homologous transcription factors in a single bacterial genome.

Sigma factor 54, alternatively named RpoN, is also required for the initiation of transcription at specific DNA sequences (Hirschman *et al.*, 1985). The σ factor facilitates transcription at specific DNA sequences by binding to the core RNA polymerase (RNAP) to form the σ‐RNAP holoenzyme, recognizing and binding to a specific DNA sequence adjacent to the transcription start site, called the promoter element, and opening the double‐stranded DNA to initiate transcription (Barrios *et al.*, 1999; Doucleff *et al.*, 2005; Wiesler *et al.*, 2012; Schaefer *et al.*, 2015).

RpoN can regulate the transcription and expression of many genes, and is historically known for its role in nitrogen assimilation (Kohler *et al.*, 1989; Rajeev *et al.*, 2015). This protein has been shown to be involved in the regulation of other important lifestyle‐associated functions in bacteria, such as in the regulation of the type III secretion system (Lee *et al.*, 2016) and type VI secretion system (Dong and Mekalanos, 2012; Sana *et al.*, 2013). RpoN also affects the growth, virulence, motility, and biofilm formation of bacteria (Hao *et al.*, 2013; Hayrapetyan *et al.*, 2015; Ray *et al.*, 2015). In addition, RpoN can affect the assembly of bacterial flagella (Yang *et al.*, 2009; Schulz *et al.*, 2012), quorum sensing (QS) (Cai *et al.*, 2015), amino acid utilization, the regulation of catalase expression at the transcriptional level (Diep *et al.*, 2015), and carbohydrate metabolism (Stevens *et al.*, 2010; Hayrapetyan *et al.*, 2015).

Analysis of the complete genome sequences of several microorganisms has shown that the *rpoN* gene (encoding the σ^54^ factor) is widely distributed among bacteria (Studholme *et al.*, 2000; Studholme and Buck, 2000a, 2000b; Dombrecht *et al.*, 2002; Studholme and Dixon, 2003). A few organisms have two copies of *rpoN*, such as *Ralstonia solanacearum* (Ray *et al.*, 2015), *Burkholderia pseudomallei* (Diep *et al.*, 2015), *Bradyrhizobium japonicum* (Kullik *et al.*, 1991), and *X. campestris* (Yang *et al.*, 2009). In *B. japonicum*, these copies are highly similar and functionally interchangeable (Kullik *et al.*, 1991). The genome of *R. solanacearum* contains two copies of *rpoN* that have different functions and are involved in twitching motility, natural competence, growth on nitrate, and virulence (Ray *et al.*, 2015). In contrast, the four different σ^54^ factors of *Rhodobacter sphaeroides* are not functionally interchangeable (Poggio *et al.*, 2002), and this unusually high number of *rpoN* gene copies does not seem to be the result of recent duplication events because the products of these genes show degrees of similarity ranging from 48% to 87% (Poggio *et al.*, 2006). However, why is it that among highly similar RpoNs some are functionally interchangeable and some are not? The molecular determinants that confer this specificity remain unknown.

**FIGURE 1:**
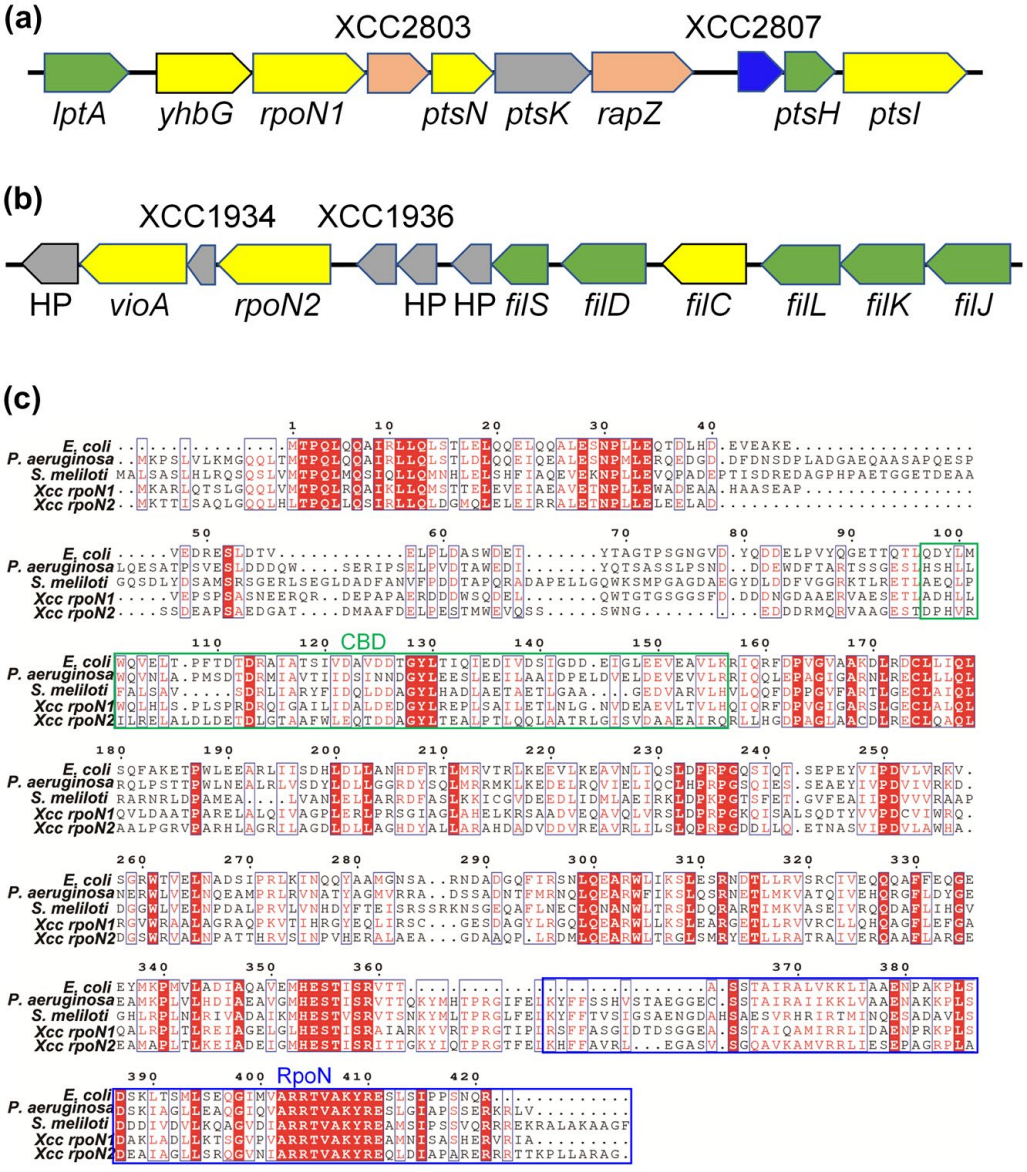
Identification and sequence characterization of RpoN in *Xanthomonas campestris.* (a) Chromosomal region of *X. campestris* surrounding the *rpoN1* gene. *ptsA*, lipopolysaccharide export system protein LptA; *yhbG*, ABC transporter ATP‐binding protein; *rpoN1*, RNA polymerase σ^54^ factor, XCC2803, σ^54^ modulation protein; *ptsN*, nitrogen regulatory IIA protein; *ptsK*, HPr kinase/phosphorylase; *rapZ*, RNase adapter protein RapZ; XCC2807, PTS, ascorbate‐specific IIA component; *ptsH*, phosphotransferase system HPr enzyme; *ptsI*, phosphotransferase system enzyme I. (b) Chromosomal region of *X. campestris* surrounding the *rpoN2* gene. HP, hypothetical protein; *vioA*, nucleotide sugar transaminase; *fleQ*, transcriptional regulator; XCC1934, response regulator; *rpoN2*, RNA polymerase σ^54^ factor; XCC1936, response regulator; HP, hypothetical protein; *fliS*, flagellar protein; *fliD*, flagellar protein; *fliC*, flagellin; *flgL*, flagellar hook‐associated protein FlgL; *flgK*, flagellar hook‐associated protein FlgK; *flgJ*, flagellar rod assembly protein/muramidase FlgJ. (c) Alignment of *X. campestris, Pseudomonas aeruginosa*, and *Sinorhizobium meliloti* RpoN sequences. Alignment was performed with ClustalW based on identical residues. CBD and RpoN are highly conserved among σ^54^ proteins from different species

A majority of bacterial species belonging to the genus *Xanthomonas* are plant pathogens. Among these species, *X. campestris* pv. *campestris* causes black rot disease in crucifers (Zhou *et al.*, 2015a; Wang *et al.*, 2018). Two *rpoN* homologues genes encoding σ^54^ were also identified in the genome of *X. campestris*. To distinguish these two genes, the *rpoN* gene XCC2802, located in a phosphotransferase system (PTS), was named *rpoN1* and the other (XCC1935), located in a large flagellar gene cluster, was named *rpoN2* (Figure 1a,b) (da Silva *et al.*, 2002; Yang *et al.*, 2009). The flagellar biogenesis of *X*. *campestris* requires the alternative sigma factor RpoN2 (da Silva *et al.*, 2002; Yang *et al.*, 2009). However, the mechanism by which RpoN2 regulates flagellar biogenesis and the role of *rpoN1* in *X. campestris* remain unknown. Whether these two different σ^54^ factors of *X. campestris* are functionally interchangeable and the molecular determinants that may confer specificity on RpoN1 and RpoN2 have not yet been investigated.

In this study, we describe for the first time the distinct roles of RpoN1 and RpoN2 and their different regulatory functions in basal metabolism, flagellar biosynthesis, extracellular polysaccharide (EPS) formation, biofilm formation, and virulence in *X. campestris*. Our study therefore reveals that these functions of the two homologous RpoN proteins are not redundant in *X. campestris*. Overall, the results of the present study improve the current understanding of RpoN‐mediated regulation in *X. campestris*.

## 2 RESULTS

### 2.1 Two conserved *rpoN* genes in the *X. campestris* genome

To investigate the function of RpoN in *X. campestris*, protein sequence alignments of *X. campestris* RpoN with RpoN proteins from *Escherichia coli* (Tucker *et al.*, 2010; Wiesler *et al.*, 2012), *Pseudomonas aeruginosa* (Shao *et al.*, 2018), and *Sinorhizobium meliloti* (Iannino *et al* *.*, 2008) were examined (Figure 1c). The results showed that the *X. campestris* RpoN1 protein shares 42%, 43%, and 36% identical residues with *E. coli* RpoN, *P. aeruginosa* RpoN, and *S. meliloti* RpoN, respectively. We also aligned the sequence of *X. campestris* RpoN2 with those of *E. coli* RpoN, *P. aeruginosa* RpoN, and *S. meliloti* RpoN, and the identity values were 40%, 39%, and 35%, respectively. The RpoN1 protein shares 56% identical residues with RpoN2 in *X. campestris*. We also found that *X. campestris* RpoN1 and RpoN2 each contain a major RNAP core‐binding domain (CBD) and RpoN domain (Yang *et al.*, 2015). Based on these criteria, it seems reasonable that RpoN1 and RpoN2 could be functional σ^54^ factors that play crucial roles in basal metabolism and virulence in *X. campestris*.

### 2.2 Inactivation of *rpoN2* caused a deficiency in virulence

**FIGURE 2:**
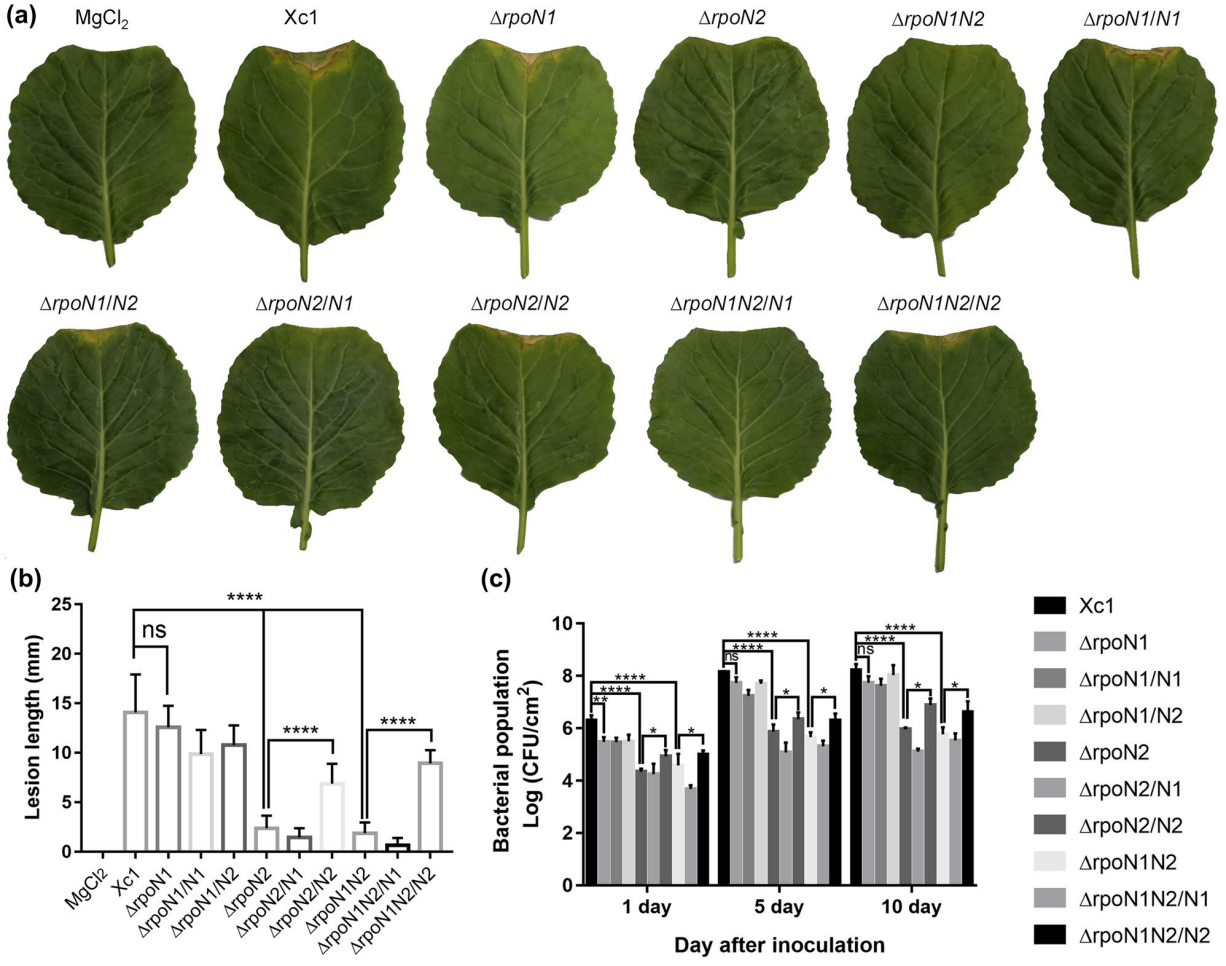
Inactivation of *rpoN2* caused a deficiency in the virulence of *Xanthomonas campestris*. (a) Bacterial strains were inoculated into leaves of the host plant *Brassica oleracea* 'Jingfeng No. 1'. Lesion length was estimated 10 days after inoculation. Sterile 10 mM MgCl_2_ was used as the negative control. (b) Lesion lengths of the wild‐type strain Xc1 and the derived strains. Virulence of the *X. campestris* strains was tested by measuring lesion length after inoculating bacteria on Jingfeng No. 1. Values are expressed as the mean and standard deviation of triplicate measurements, each comprising 20 leaves. (c) Bacterial counts in the top 2.5 cm^2^ of each lesion‐exhibiting leaf were scored. Error bars, means ± *SD* (*n* = 3). *p < .05, **p < .01, ****p < .0001, assessed by one‐way analysis of variance. All experiments were repeated three times with similar results

*X. campestris* is the causal agent of black rot disease in cruciferous vegetables. To identify the physiological functions of RpoN1 and RpoN2 in virulence against host plants, the gene knockout strains Δ*rpoN1* and Δ*rpoN2* and the double‐mutant strain Δ*rpoN1N2*, in which both the *rpoN1* (XCC2802) and *rpoN2* (XCC1935) genes were deleted, were constructed by a two‐step homologous recombination approach. To further evaluate whether RpoN contributes to the virulence of *X. campestris*, a leaf‐clipping virulence assay using a susceptible cabbage variety (*Brassica oleracea* 'Jingfeng No. 1') was conducted. The average lesion length caused by wild‐type strain Xc1 on a cabbage leaf was 14.1 mm at 10 days after inoculation (Figure 2a,b). The gene knockout strains Δ*rpoN2* and Δ*rpoN1N2* exhibited significantly reduced average lesion lengths (2.4 and 1.9 mm, respectively) (Figure 2a,b), but the lesion length of the Δ*rpoN1* strain was not significantly different compared with that of the wild‐type strain (12.6 mm). Under similar test conditions, complementation with *rpoN2* restored the virulence of Δ*rpoN2* and Δ*rpoN1N2* against host plants, and the average lesion lengths with the complemented strains were 6.9 and 8.9 mm, respectively (Figure 2a,b).

The bacterial population in planta was also measured. After inoculation, the bacterial populations of Δ*rpoN2* and Δ*rpoN1N2* were markedly decreased in planta (Figure 2c). The complemented strains Δ*rpoN2/N2* and Δ*rpoN1N2/N2*, containing a plasmid‐borne *rpoN2*, retained the wild‐type colonization ability in planta (Figure 2c). This result indicates that *rpoN2*, but not *rpoN1*, is required for the virulence of *X. campestris*, indicating that RpoN1 and RpoN2 are not functionally interchangeable in terms of the pathogenicity of *X. campestris*.

### 2.3 Transcriptomic profiling of the *rpoN* deletion strains revealed the regulatory role of this gene in basal metabolism and flagellar synthesis

**FIGURE 3:**
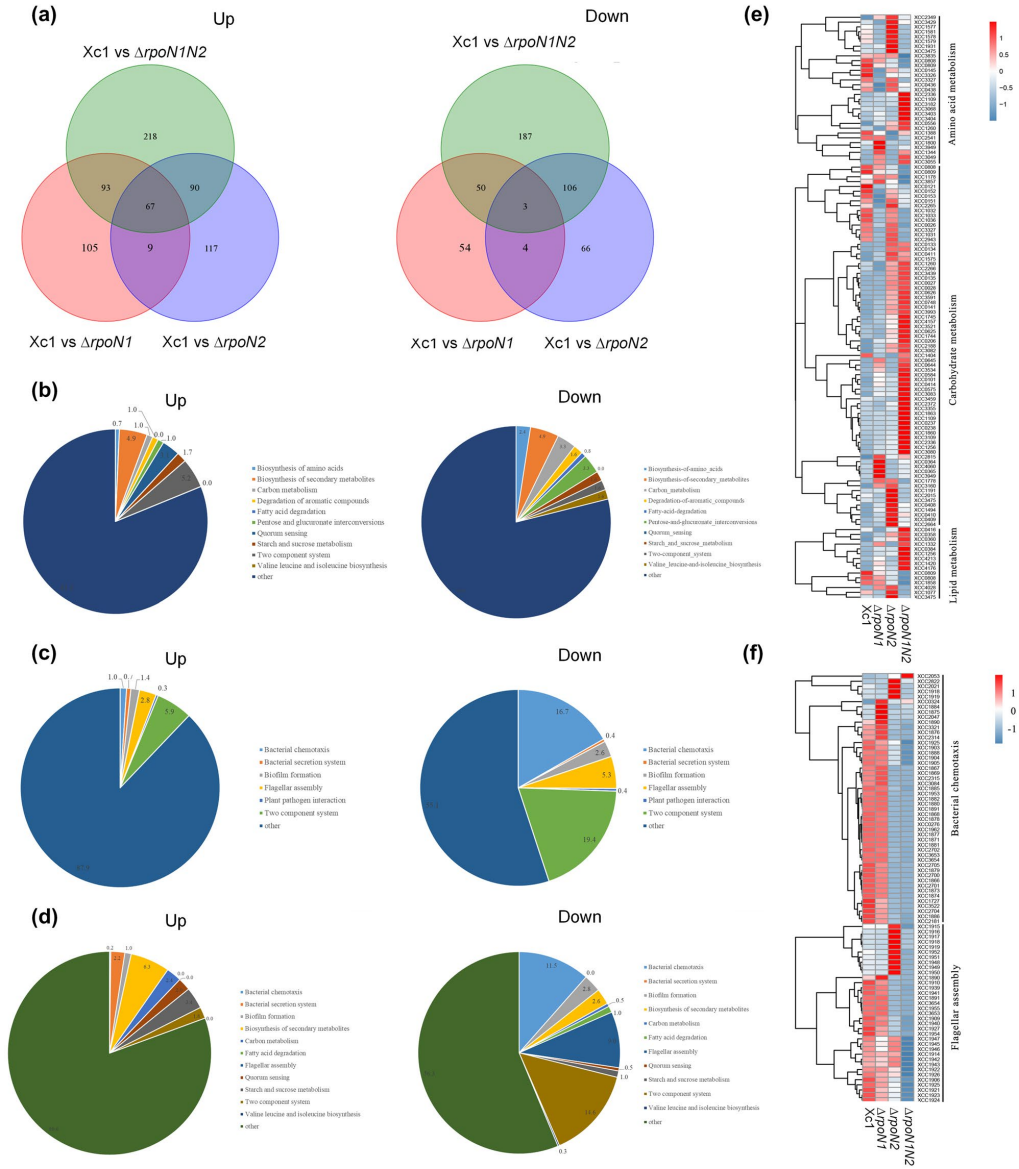
Differentially expressed genes (DEGs) involved in basal metabolism and flagellar synthesis. (a) Venn diagram showing overlap analysis of the up‐regulated and down‐regulated DEGs in the wild‐type Xc1, ∆*rpoN1*, ∆*rpoN2*, and ∆*rpoN1N2*. The Venn diagrams were drawn with the “Venn Diagram” package in R. (b) Categories of 385 significant genes assigned in the KEGG database in the Δ*rpoN1* mutant compared to the wild‐type strain: the 385 significant (*p* < .05, ≥ ±1 log_2_fold change [FC]) genes were classified into 11 different categories with diverse cellular functions. (c) Categories of 462 significant genes assigned in the KEGG database in the Δ*rpoN2* mutant compared to the wild‐type strain: the 462 significant (*p* < .05, ≥ ±log_2_FC) genes were classified into seven different categories with diverse cellular functions. (d) Categories of 814 significant genes assigned in the KEGG database in the Δ*rpoN1N2* double mutant compared to the wild‐type strain: the 814 significant (*p* < .05, ≥ ±log_2_FC) genes were classified into 12 different categories with diverse cellular functions. (e) Hierarchical cluster analysis applied to the 121 DEGs in the amino acid metabolism, carbohydrate metabolism, and lipid metabolism pathways in different mutant backgrounds. The transcriptional profiles in terms of relative gene expression values (log_2_ scale of microarray values) were analysed using the heatmap command of R. Red and blue represent up‐regulated and down‐regulated genes, respectively. (f) Hierarchical cluster analysis applied to the 84 DEGs in the bacterial chemotaxis and flagellar assembly pathway in different mutant backgrounds

To investigate whether there are new genes/functions that are controlled by *rpoN1* and *rpoN2* in *X. campestris*, we performed transcriptomic analyses (RNA‐Seq) of the wild‐type and *rpoN* mutant strains. The examination of each sample was repeated twice. The results show that compared to the wild type, a total of 385 genes were significantly differentially altered at the transcriptional level in the Δ*rpoN1* mutant strain, with 274 genes up‐regulated and 111 genes down‐regulated (Figure 3a and Table S3). We also analysed and compared the transcriptomes of the Δ*rpoN2* mutant strain and wild‐type strain using RNA‐Seq. Differential gene expression analysis showed that 283 genes were up‐regulated and 179 genes were down‐regulated in the Δ*rpoN2* mutant compared with the wild‐type strain, whereas 468 genes were up‐regulated and 346 genes were down‐regulated in the Δ*rpoN1N2* double mutant compared with the wild‐type strain (Figure 3a, Tables S4 and S5). We also compared the transcriptome profiles using Venn diagrams, which showed the overlap of 67 up‐regulated and three down‐regulated genes in different mutant backgrounds (Figure 3a). These results were further partly confirmed by quantitative reverse transcription PCR (RT‐qPCR) analysis of eight randomly selected candidate genes (Figure S1). Further bioinformatics analyses showed that the products of these differentially expressed genes (DEGs) belong to three major functional categories. In addition, each major category contains rather diverse subfunctional groups (Figure S2a–c). Notably, the *rpoN* mutants induced significant changes in mRNA abundance in the genes involved in basal metabolism and flagellar synthesis, as indicated by functional annotation analysis (Figure 3b–d). Many genes involved in basal metabolism were differentially expressed in the Δ*rpoN1* mutant and Δ*rpoN1N2* double mutant (Figure 3e). Additionally, the transcript levels of 35 flagellar assembly pathway genes and 49 chemotaxis‐related genes were significantly altered in the Δ*rpoN2* mutant and Δ*rpoN1N2* double mutant (Figure 3f). Taken together, our RNA‐Seq data reveal that the transcripts of genes involved in basal metabolism and flagellar synthesis were substantially affected by the *rpoN1* and *rpoN2* genes.

### 2.4 Deletion of *rpoN2* affected the swimming ability and flagellar synthesis of *X. campestris*

**FIGURE 4:**
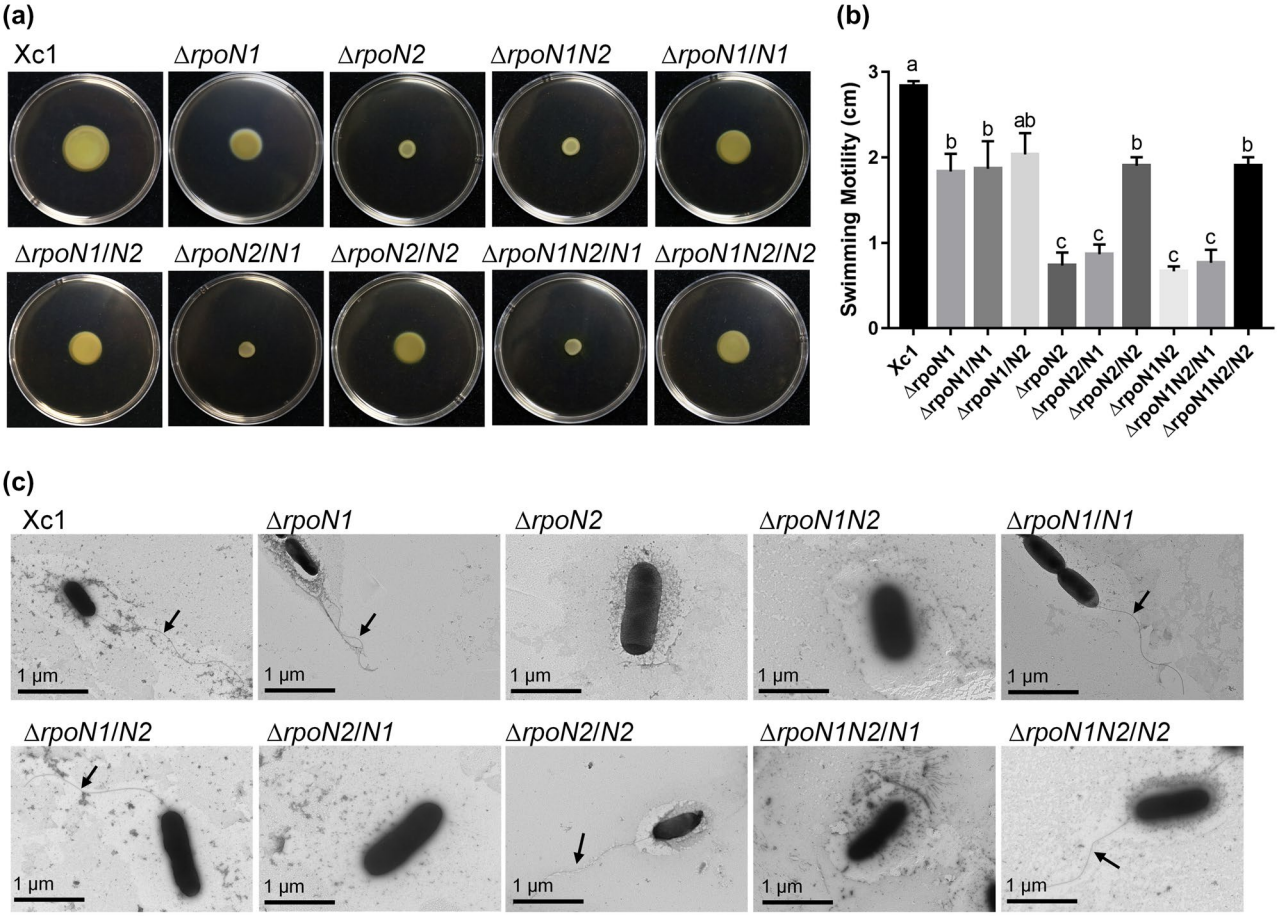
Deletion of *rpoN2* affected *Xanthomonas campestris* swimming ability and flagellar synthesis. (a) and (b) Assay of swimming motility of the wild‐type Xc1; mutants Δ*rpoN1*, Δ*rpoN2*, and Δ*rpoN1N2*; and complemented strains Δ*rpoN1/N1*, Δ*rpoN1/N2*, Δ*rpoN2/N1*, Δ*rpoN2/N2*, Δ*rpoN1N2/N1*, and Δ*rpoN1N2/N2*. The swimming zones were recorded after bacterial growth for 4 days on semisolid plates at 28 °C. Error bars, means ± *SD* (*n* = 3). Different letters indicate significant differences between treatments with the least significant difference at *p* = .05. All experiments were repeated three times with similar results. (c) Observation of flagella using transmission electron microscopy

Xcc *rpoN2* (XCC1935) is located in a flagellar synthesis gene cluster (Figure 1a). A series of flagellar synthesis‐related genes (*filDCES*, *flhAB*) and chemotaxis‐related genes (*cheABDRWY*, *motAB*) were highlighted in the RNA‐Seq data, and all of these genes were down‐regulated in the Δ*rpoN2* mutant (Table S4). We hypothesized that an in‐frame deletion mutant of *rpoN2* affected flagellar biogenesis. To further determine whether deletion of *rpoN2* affected flagellar biogenesis in *X. campestris*, the single polar flagellum of various *X. campestris* strains was observed by transmission electron microscopy (TEM). The electron micrographs demonstrated that the Δ*rpoN2* and Δ*rpoN1N2* mutants lost the typical single polar flagellum, and recovery to wild‐type levels was observed in the *rpoN2*‐complemented strains but not in the *rpoN1*‐complemented strains (Figure 4c). TEM observation showed that the Δ*rpoN1* mutant strain had a normal flagellar morphology compared with the wild‐type strain (Figure 4c). These results indicate that RpoN2 is necessary for flagellar biogenesis and motility in *X. campestris* and suggest that the function of RpoN2 in flagellar synthesis cannot be performed by RpoN1.

The function of *rpoN1* and *rpoN2* in flagellum‐dependent motility was investigated. We then tested the swimming motility of the Δ*rpoN1*, Δ*rpoN2*, and Δ*rpoN1N2* mutants on 0.3% semisolid agar plates. Compared with the wild‐type strain, deletion of *rpoN2* abolished swimming motility in *X. campestris*, suggesting that the motility was significantly impaired (Figure 4a,b). The Δ*rpoN1* mutant remained as motile as the wild type. The motility defect phenotypes of the Δ*rpoN2* and Δ*rpoN1N2* mutants could be restored to wild‐type levels by introducing *rpoN2* into Δ*rpoN2* and Δ*rpoN1N2*, respectively. Moreover, overexpression of *rpoN1* in the Δ*rpoN2* and Δ*rpoN1N2* mutants resulted in no substantial difference between the mutant and complemented strains of *X. campestris* in terms of swimming motility (Figure 4a,b). These results suggest that RpoN2, but not RpoN1, is required for the swimming motility of *X. campestris*.

**FIGURE 5:**
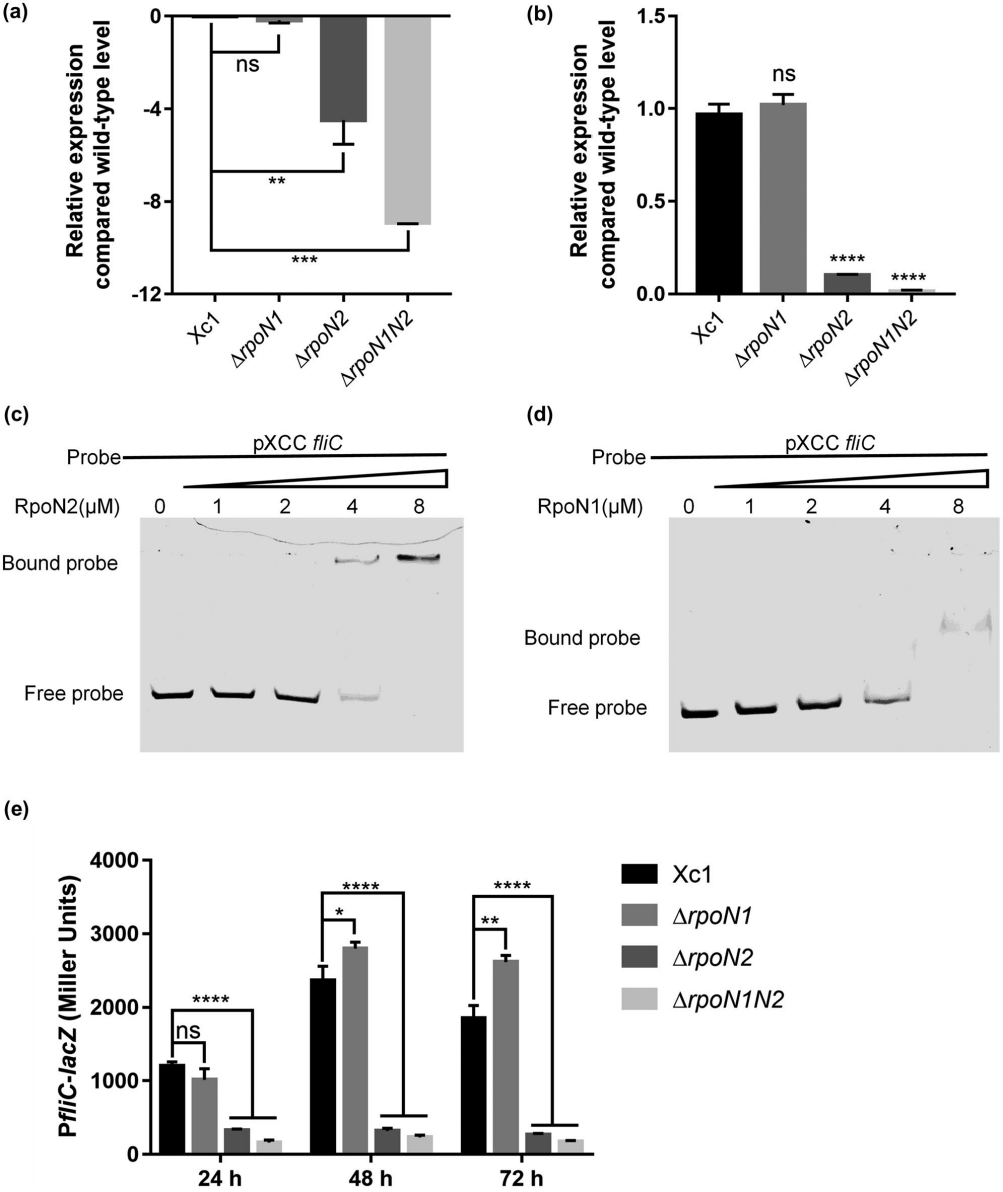
The effects of RpoN2 on binding to the promoter of *Xanthomonas campestris* pv. *campestris fliC*. (a) Relative expression of *fliC* as determined by RNA‐Seq. (b) Relative expression of *fliC* as determined by quantitative reverse transcription PCR. (c) and (d) Gel shift assay showing that RpoN2 and RpoN1 directly regulate *fliC*. RpoN2 and RpoN1 (0, 1, 2, 4, or 8 μM) were added to reaction mixtures containing 50 ng of probe DNA, and the reaction mixtures were separated on polyacrylamide gels. (e) The effect of RpoN on *fliC* gene expression was measured by assessing the β‐galactosidase activity of the *fliC*‐*lacZ* transcriptional fusions in the Xc1 wild‐type, Δ*rpoN1*, Δ*rpoN2*, and Δ*rpoN1N2* strains. Error bars, means ± *SD* (*n* = 3). *p < .05, **p < .01, ****p < .0001, assessed by one‐way analysis of variance. All experiments were repeated three times with similar results

To further demonstrate the biological function of RpoN, we performed electrophoretic mobility gel shift assays (EMSA) without a core RNAP (Cannon *et al.*, 1993). The RpoN1 and RpoN2 proteins were then expressed in *E. coli* BL21 (DE3), and the N‐terminal‐His_6_‐tagged versions of the proteins were purified with nickel chelate chromatography (Figure S3). To determine the direct link between RpoN and flagellar biogenesis genes, RpoN‐regulated candidate genes of *X. campestris* were selected for further investigation. Deletion of *rpoN2* resulted in down‐regulation of Xcc *fliC* (Figure 5a), which suggests that RpoN might bind directly to the promoter region of Xcc *fliC* to regulate transcription, in turn affecting the flagellar synthesis in *X. campestris*. To test this hypothesis, the putative promoter DNA fragment covering 409 bp upstream of the Xcc *fliC* translational start site, namely, pXCC *fliC*, was cloned and analysed using EMSA. Addition of purified RpoN2 protein, at concentrations ranging from 0 to 8 µM, to the reaction mixtures (20 μl, 28 °C, 25 min) caused a shift in the mobility of the pXCC *fliC* DNA fragment, which suggested that an RpoN2‐pXCC *fliC* complex was formed (Figure 5c). However, addition of purified RpoN1 protein indicated weak binding to the promoter of Xcc *fliC* (Figure 5d). RT‐qPCR showed that the expression of Xcc *fliC* in the Δ*rpoN2* and Δ*rpoN1N2* mutant strains was compromised compared with that in the wild‐type *X. campestris* strain, indicating that RpoN2 positively regulates the expression of Xcc *fliC* (Figure 5b). To further study regulation by RpoN, we constructed p*fliC*‐*lacZ* reporter systems in *rpoN* mutant strains. Consistent with the RNA‐Seq and RT‐qPCR results, deletion of *rpoN2* resulted in reduced expression levels of XCC *fliC* (Figure 5e). We found that RpoN2 can also bind the promoter of Xcc *fliQ* (Figure S4). Taken together, these results indicate that RpoN2 positively regulates the Xcc *fliC* and Xcc *fliQ* genes by directly binding to the corresponding promoters to regulate flagellar synthesis in *X. campestris*.

### 2.5 RpoN2 is involved in biofilm formation and EPS production

**FIGURE 6:**
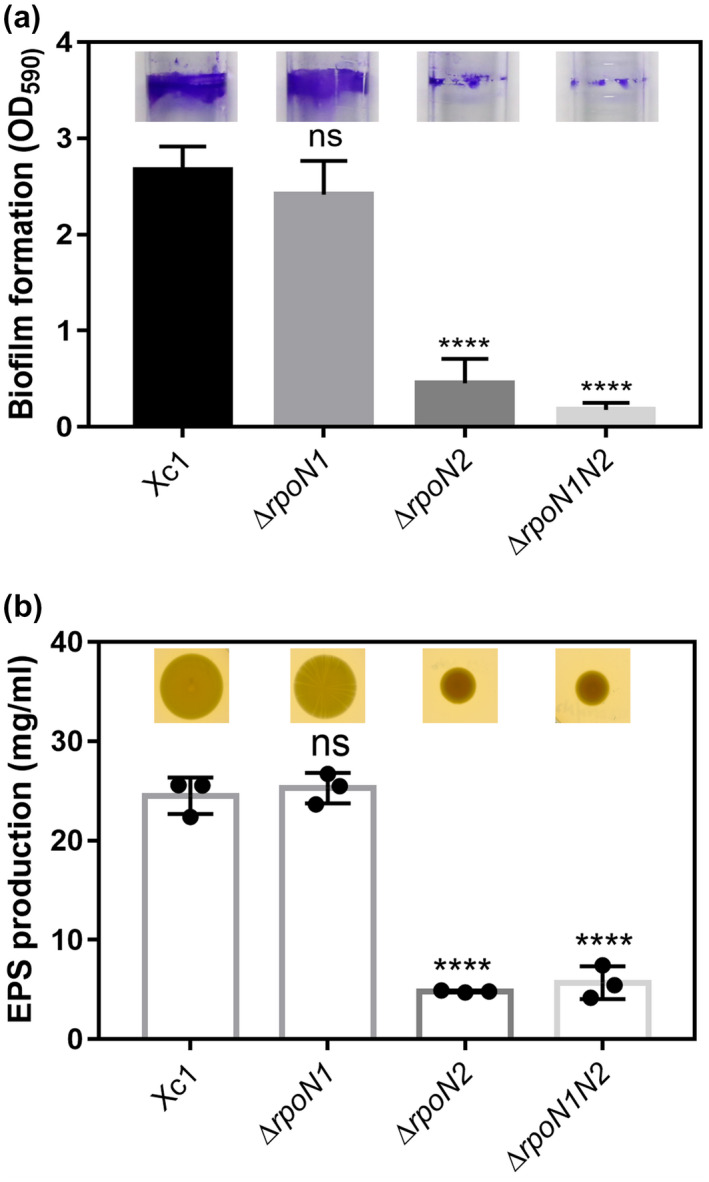
Effects of *rpoN* genes on the production of biofilms and extracellular polysaccharide (EPS) in *Xanthomonas campestris*. (a) Deletion of *rpoN2* in *X. campestris* Xc1 affects biofilm formation in nutrient yeast glycerol (NYG) broth, as determined by crystal violet (CV) staining. (b) Deletion of *rpoN2* in *X. campestris* affects EPS production in NYG broth. Error bars, means ± *SD* (*n* = 3). ****Significant differences (*p* < .0001, assessed by one‐way analysis of variance). All experiments were repeated three times with similar results

Bacterial biofilms are involved in adaptation to complex environments and are used by pathogenic bacteria to colonize host cells (Tao *et al.*, 2010). To test whether RpoN plays a role in biofilm formation, three independent assays were carried out. In the first assay, the frequently used crystal violet (CV) staining approach was applied. The results showed that the Δ*rpoN2* and Δ*rpoN1N2* mutant strains exhibited 5.8‐fold and 14.9‐fold reductions, respectively, in biofilm formation on the polystyrene surface after staining with CV, compared with the wild type (Figure 6a); however, inactivation of *rpoN1* had no effect on biofilm formation (Figure 6a). These data reveal that RpoN2 is required for full biofilm formation.

EPS is a  critical virulence factor for pathogenicity (Rai *et al.*, 2012; Cai *et al.*, 2017). Yu *et al*. found that RpoN (σ^54^) is required for floc formation but not for EPS biosynthesis in a floc‐forming *Aquincola tertiaricarbonis* strain (Yu *et al.*, 2017). To further investigate the function of Xcc RpoN in EPS production, we examined EPS synthesis in the *rpoN* mutants. As shown in Figure 6b, the EPS production ability of the Δ*rpoN2* and Δ*rpoN1N2* mutants was significantly decreased, but EPS production by the Δ*rpoN1* strain was not significantly different compared with that by the wild‐type strain. These findings suggest that RpoN2 is required for full EPS production.

### 2.6 Deletion of *rpoN1* affected the synthesis of branched‐chain fatty acids and the production of diffusible signalling factor family signals in *X. campestris*

**FIGURE 7:**
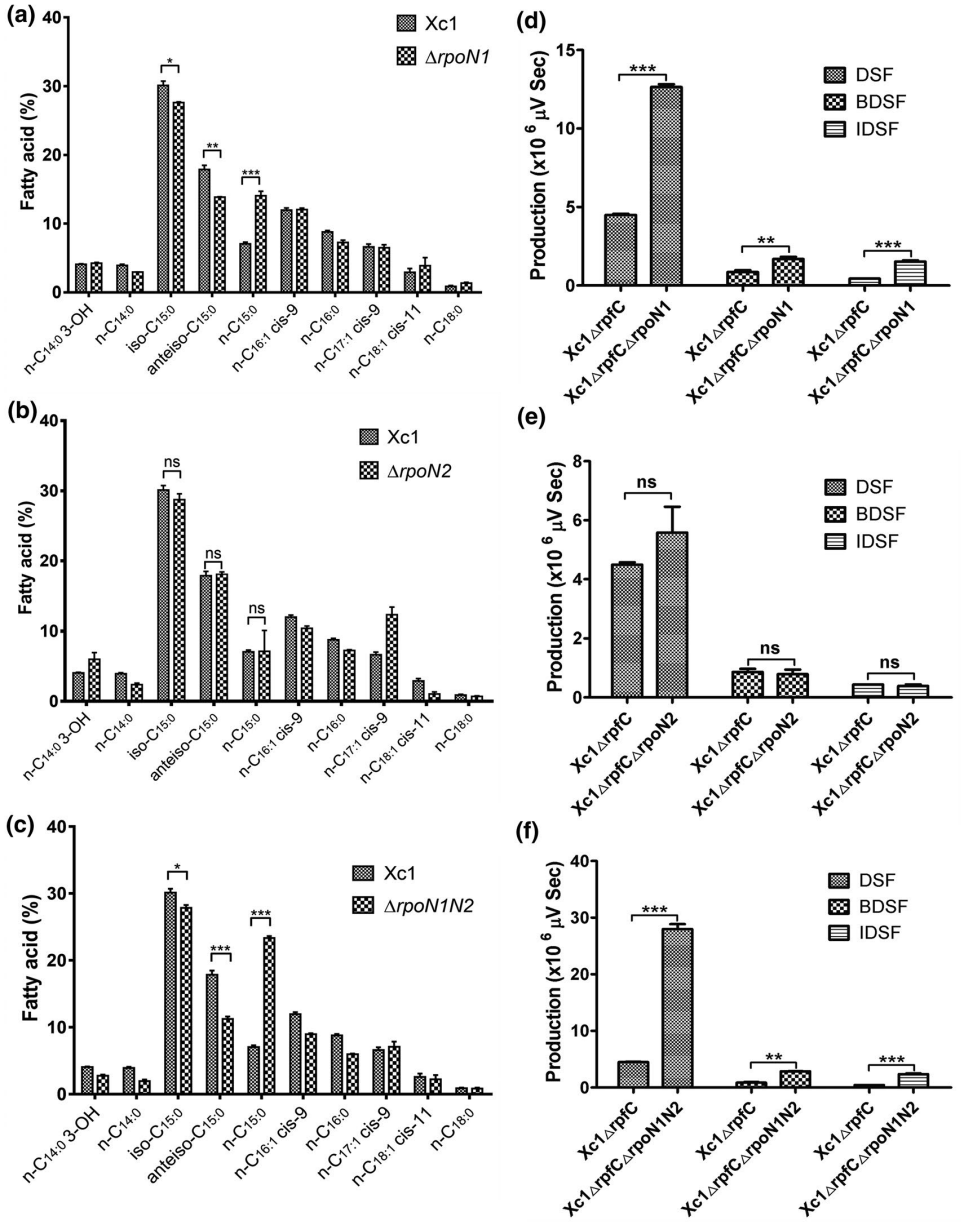
Deletion of *rpoN1* affected the synthesis of branched‐chain fatty acids and increased the production of diffusible signalling factor (DSF) family signals in *Xanthomonas campestris*. (a) Fatty acid composition of the total lipid extracts from the wild‐type Xc1 and Δ*rpoN1* mutant strains grown in nutrient yeast glycerol (NYG) medium. (b) Fatty acid composition of the total lipid extracts from the Xc1 and Δ*rpoN2* mutant strains grown in NYG medium. (c) Fatty acid composition of the total lipid extracts from the Xc1 and Δ*rpoN1N2* double‐mutant strains grown in NYG medium. Total lipids were extracted and transesterified to fatty acid methyl esters, and the products were identified by gas chromatography‐mass spectrometry (GC‐MS). Values are percentages of total fatty acid levels and are means ± *SD* of three independent experiments. *n*‐C_14:0_ 3‐OH, 3‐hydroxyltetradecanoic acid; *n*‐C_14:0_, tetradecanoic acid; *iso*‐C_15:0_, 13‐methyl‐tetradecanoic acid; *anteiso*‐C_15:0_, 12‐methyl‐tetradecanoic acid; *n*‐C_15:0_, pentadecanoic acid; *n*‐C_16:1_, *cis*‐9‐hexadecenoic acid; *n*‐C_16:0_, hexadecanoic acid; *iso*‐C_17:1_ *cis*‐9, *cis*‐9‐15‐methyl‐hexadecenoic acid; *n*‐C_18:1_ *cis*‐11, *cis*‐11‐octadecenoic acid; *n*‐C_18:0_, octadecanoic acid. (d) DSF signals produced by the *rpoN1* deletion mutant Δ*rpoN1* grown in nutrient broth (NB) medium. Supernatants (50 ml) of strain Δ*rpfC*Δ*rpoN1* grown in NB medium for 36 hr were collected and DSF signals were detected. (e) DSF signals produced by the *rpoN2* deletion mutant Δ*rpoN2* grown in NB medium. Supernatants (50 ml) of the strain Δ*rpfC*Δ*rpoN2* grown in NB medium for 36 hr were collected and DSF signals were detected. (f) DSF signals produced by the *rpoN1* and *rpoN2* double‐mutant Δ*rpoN1N2* grown in NB medium. Supernatants (50 ml) of strain Δ*rpfC*Δ*rpoN1N2* grown in NB medium for 36 hr were collected and DSF signals were detected. DSF, *cis*‐11‐methyl‐2‐dodecenoic acid; BDSF, *cis*‐2‐dodecenoic acid; IDSF, *cis*‐10‐methyl‐2‐dodecenoic acid. Error bars, mean ± *SD* (*n* = 3). *p < .05, **p < .01, ***p < .001, assessed by one‐way analysis of variance. All experiments were repeated three times with similar results

Xcc *rpoN1* (XCC2802) is located in a phosphotransferase gene cluster (Figure 1a). The transcription of genes involved in the basal metabolism pathway, including genes involved in carbon metabolism, biosynthesis of secondary metabolites, starch and sucrose metabolism, pentose and glucuronate interconversion, biosynthesis of amino acids, QS and other pathways was changed in the Δ*rpoN1* mutant cells. To test the function of RpoN in basal metabolism, we analysed the fatty acid composition of the total lipid extracts from Xcc *rpoN* mutant strains grown in nutrient yeast glycerol (NYG) medium at 28 °C by gas chromatography‐mass spectrometry (GC‐MS). Compared to the fatty acid composition of the wild‐type strain Xc1 grown in NYG medium at 28 °C, the Δ*rpoN1* mutant strain and Δ*rpoN1N2* double‐mutant strain exhibited significantly decreased amounts of branched‐chain fatty acids (BCFAs), especially *iso*‐C_15:0_ and *anteiso*‐C_15:0_ fatty acids, and increased the amount of pentadecanoic acid (*n*‐C_15:0_). The fatty acid profile of Xcc Δ*rpoN2* did not differ from that of wild‐type strain Xc1 under these temperature conditions (Figure 7a–c). These results suggest that Xcc RpoN1 is crucial for fatty acid synthesis in strain Xc1.

To further evaluate the function of RpoN in basal metabolism, we also tested the production of diffusible signalling factor (DSF) family signals by *rpoN* mutant strains grown in NYG medium using high‐performance liquid chromatography (HPLC). As the Xc1 *rpfC* deletion strain produced high levels of DSF family signals (Zhou *et al.*, 2015b), sufficient for analysis, we constructed the mutant strains Δ*rpfC*Δ*rpoN1*, Δ*rpfC*Δ*rpoN2*, and the double‐mutant strain Δ*rpfC*Δ*rpoN1N2* by deleting *rpoN1* and *rpoN2* from the parental strain Xc1 Δ*rpfC*. DSF family signal production was further studied in NYG medium. All the strains produced DSF family signals, namely, *cis*‐11‐methyl‐2‐dodecenoic acid (DSF), *cis*‐2‐dodecenoic acid (BDSF), and *cis*‐10‐methyl‐2‐dodecenoic acid (IDSF), but DSF was the main signal produced under these conditions (Figure 7d–f), consistent with the result of a previous report (Zhou *et al.*, 2015b; Li *et al.*, 2017). However, the amounts of each DSF family signal produced by the mutant strain Δ*rpfC*Δ*rpoN1* and double‐mutant strain Δ*rpfC*Δ*rpoN1N2* were significantly higher than those produced by the Xc1 Δ*rpfC* strain (*p* < .001). These findings indicated that *rpoN1* deletion increased the synthesis of DSF family signals in *X. campestris*, especially the amount of DSF.

## 3 DISCUSSION

RpoN has been shown to be involved in the regulation of many bacterial functions, such as nitrogen metabolism, flagellar biosynthesis, biofilm formation, motility, colonization, lipoprotein biosynthesis, and the activity of a type III secretion system (Kohler *et al.*, 1989; Yang *et al.*, 2009; Dong and Mekalanos, 2012; Schulz *et al.*, 2012; Hao *et al.*, 2013; Sana *et al.*, 2013; Hayrapetyan *et al.*, 2015; Rajeev *et al.*, 2015; Ray *et al.*, 2015; Lee *et al.*, 2016). RpoN also affects bacterial QS (Cai *et al.*, 2015). Although RpoN regulates bacterial virulence and shares similar functions in gram‐negative bacteria, the regulons of RpoN vary among bacteria (Schaefer *et al.*, 2015; Shao *et al.*, 2018; Xu *et al.*, 2019). In this study, we demonstrate that RpoN1 and RpoN2 play different roles in regulation of basal metabolism, flagellar biosynthesis and virulence traits in *X. campestris*.

Our data show that in‐frame deletions of the *rpoN2* coding sequence caused a significant decrease in bacterial virulence and in planta growth in a susceptible cabbage variety (*B. oleraceae* 'Jingfeng No. 1') (Figure 2), whereas the Δ*rpoN1* mutant did not cause a substantial decrease in virulence or in planta growth. However, why does the *rpoN2* mutation cause a decrease in *X. campestris* virulence? To understand the underlying mechanisms, the role of *rpoN2* in *X. campestris* virulence was examined. We evaluated several pathogenicity‐related virulence factors produced by the *rpoN* mutant strains. The activities of extracellular cellulase, amylase, and protease enzymes produced by the *rpoN* mutant strains were not significantly different from those of the enzymes produced by the wild‐type strain Xc1 (Figure S5). However, we evaluated EPS production in the *rpoN* mutant strains, and found that the ability of the Δ*rpoN2* and Δ*rpoN1N2* mutants to produce EPS was significantly decreased (Figure 6b). EPS act as a matrix material and is essential for biofilm formation in most gram‐negative bacteria (Pratt and Kolter, 1999; Donlan, 2002; Singh *et al.*, 2006). Does this finding indicate that the *rpoN2* mutant also affects biofilm formation in *X. campestris*? We tested the biofilm formation ability of Δ*rpoN1*, Δ*rpoN2*, and Δ*rpoN1N2*, and found that Δ*rpoN2* and Δ*rpoN1N2* exhibited significantly reduced biofilm formation compared with the wild type (Figure 6a). Bacterial biofilms are involved in adaptation to complex environments and are used by pathogenic bacteria to colonize host cells (Tao *et al.*, 2010). Thus, we hypothesized that reduction in EPS and biofilm formation was one of the reasons why *X. campestris* exhibited impaired virulence against host plants. However, further investigation is required to understand these mechanisms in greater detail.

RpoN has been implicated in the regulation of flagellar and motility genes in many species. We also showed that the absence of native RpoN2 significantly reduced motility‐associated phenotypes (Figure 4a–c). For many bacteria, motility is essential for survival, growth, virulence, biofilm formation, and intra/interspecies interactions and allows the bacteria to position themselves in appropriate locations at appropriate times (Nan and Zusman, 2016; Kilmury and Burrows, 2018). In prokaryotes, flagellation is essential for swimming motility. Flagellar biogenesis is a complex process that involves over 40 genes. The phytopathogen *X. campestris* possesses a single polar flagellum (Yang *et al.*, 2009). RNA‐Seq analysis revealed that the Δ*rpoN2* and Δ*rpoN1N2* mutations affected a group of genes associated with flagellar assembly, and 55.6% and 100% of these genes, respectively, were down‐regulated (Tables S4 and S5). This result suggests that the Δ*rpoN2* mutant could be disruptive to the flagellar system with subsequent disorganization of flagellar assembly. To confirm the effect of the Δ*rpoN2* mutation on *X. campestris* flagellation, we used TEM to observe the filaments of the Xc1, Δ*rpoN1*, Δ*rpoN2*, and Δ*rpoN1N2* strains and the *rpoN1‐* and *rpoN2*‐complemented strains. TEM observation showed that deletion of *rpoN2* led to defects in flagellar morphology compared with the wild‐type strain (Figure 4a). We found that RpoN1 and RpoN2 are not functionally interchangeable for *X. campestris* flagellation (Figure 4a). This discovery can provide experimental reference information for analysis of the biological functions of RpoN2 for *X. campestris* flagellar assembly in the future. RpoN binds to cognate promoters containing a TGGCACN_5_TTGCW motif (Barrios *et al.*, 1999; Doucleff *et al.*, 2005; Wiesler *et al.*, 2012; Schaefer *et al.*, 2015). To determine the genome‐wide variation caused by *X. campestris* RpoN, the conserved RpoN consensus binding motif was also used to search the complete genomic sequences of *X. campestris* to identify the conserved GC sequences in the promoter of Xcc *fliC*. We hypothesized that RpoN2 binds to the promoter of Xcc *fliC* to regulate *X. campestris* flagellation. We demonstrated by EMSA that RpoN2 could positively regulate the expression of Xcc *fliC* by directly binding to the promoter of this gene, but RpoN1 exhibited weak binding to the Xcc *fliC* promoter (Figure 5). We found that RpoN2 can also bind to the promoter of Xcc *fliQ* (Figure S4). Taken together, these results suggest that RpoN2 might be a transcriptional activator that modulates bacterial flagellation by directly binding to the promoters of Xcc *fliC* and Xcc *fliQ* in *X. campestris*. However, further details need to be studied in the future.

Fatty acids and DSF family QS signals are critical secondary metabolites for bacterial physiological metabolism (Zhou *et al.*, 2015a; Li *et al.*, 2017; Hu *et al*., 2018). In this study, in‐frame deletion of *rpoN1* led to significantly reduced production of branched‐chain amino acids (BCAAs) and increased production of DSF family QS signalling molecules, especially *cis*‐11‐methyl‐2‐dodecenoic acid. The precursors of the DSF family signals originate from the fatty acid synthesis pathway (Yu *et al.*, 2016; Li *et al.*, 2017). However, BCAAs are the primary precursors for the synthesis of BCFAs (Zhu *et al.*, 2005). RpoN also affects amino acid utilization and carbohydrate metabolism (Stevens *et al.*, 2010; Diep *et al.*, 2015; Hayrapetyan *et al.*, 2015). Therefore, we speculate that *rpoN1* directly regulates the biosynthesis of amino acids and basal metabolism to affect BCFA and DSF synthesis. RNA‐Seq showed that the *rpoN1* mutation significantly affected the expression of a valine, leucine, and isoleucine biosynthesis‐related gene (*leuA*) and a long‐chain fatty acid‐CoA ligase gene (*rpfB*) (Figure S6 and Tables S3 and S5). These results suggest that RpoN1 and RpoN2 might play different regulatory roles in the basal metabolism of *X. campestris*. However, elucidation of the detailed mechanism requires further investigation.

**FIGURE 8:**
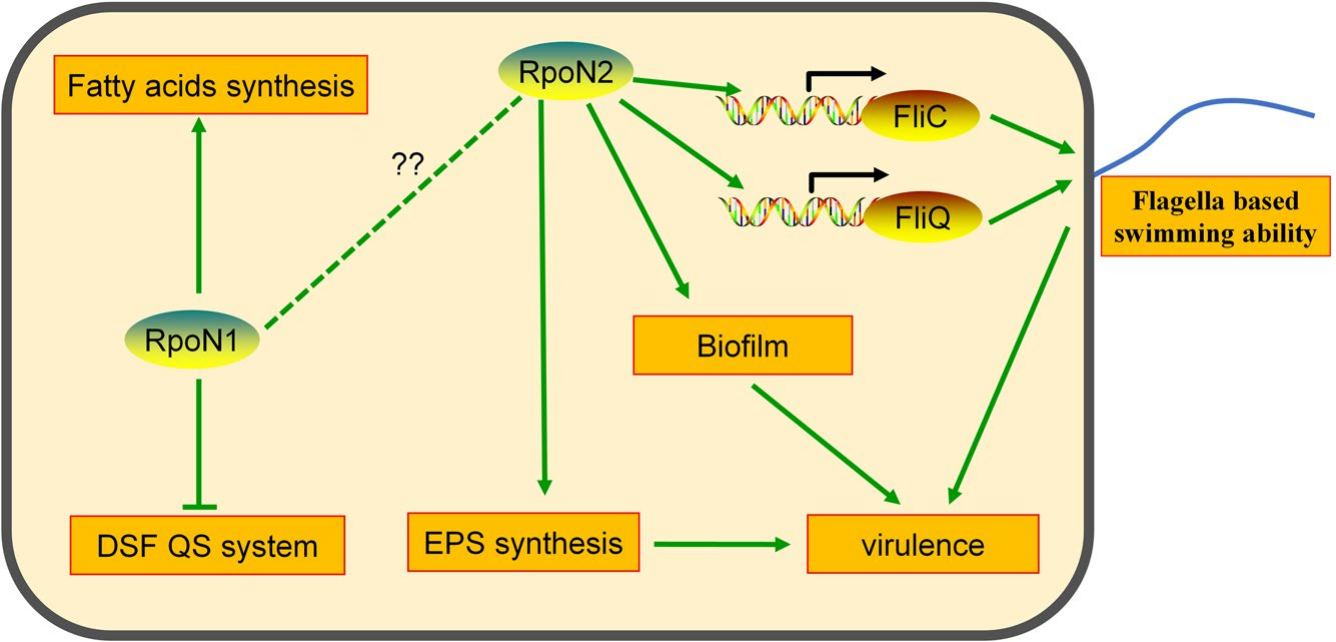
Schematic of the proposed RpoN‐mediated regulation of flagellar synthesis, virulence, extracellular polysaccharide (EPS) production, biofilm formation, quorum‐sensing (QS), and basal metabolic pathways. The potential regulatory pathways and interactions of RpoN are proposed according to our observations and previous studies. RpoN1 could regulate branched‐chain fatty acid production and modulate the synthesis of diffusible signalling factor (DSF) signals in *Xanthomonas campestris*. In the present study, we showed that RpoN2 directly bound to the *fliC* and *fliQ* promoter regions and positively regulated the expression of the flagellar assembly genes *fliC* and *fliQ*. In addition, RpoN regulated the production of EPS and biofilms, which are also used by *X. campestris* and other pathogenic bacteria as pathogenicity‐related virulence factors against host plants

Based on our results, we propose a schematic model, as shown in Figure 8. In summary, *X. campestris* RpoN2 modulates biofilm formation and EPS production, and regulates bacterial flagellation via direct binding to the promoters of Xcc *fliC* and Xcc *fliQ*. These factors together cause weakening of *X. campestris* virulence. Moreover, RpoN1 could regulate fatty acid synthesis and DSF production in *X. campestris*.

## 4 EXPERIMENTAL PROCEDURES

### 4.1 Materials

The antibiotics were obtained from Sigma‐Aldrich. Takara Biotechnology Co. provided the molecular biology reagents. Novagen provided the pET vectors. Ni‐agarose columns were obtained from Sigma‐Aldrich. Bio‐Rad provided Quick Start Bradford dye reagent. All other reagents were of the highest available quality. Genscript (Nanjing, Jiangsu, China) synthesized the oligonucleotide primers.

### 4.2 Bacterial strains, plasmids, and growth conditions

The strains and plasmids used in this study are listed in Table S1. *E. coli* strains were grown in Luria Bertani medium (10 g/L tryptone, 5 g/L yeast extract, 10 g/L NaCl, pH 7.0) at 37 °C. *X. campestris* strains were grown at 28 °C in NYG medium (5 g/L peptone, 3 g/L yeast extract, 20 g/L glycerol, pH 7.0) or nutrient broth agar (NA) (5 g/L peptone, 3 g/L beef extract, 10 g/L sucrose, 1 g/L yeast extract, pH 7.0). For culture medium preparation, tryptone, peptone, beef extract, and yeast extract were purchased from Sangon Biotech. When required, antibiotics were added at the following concentration: 100 μg/ml sodium ampicillin, 30 μg/ml kanamycin sulphate, and 30 μg/ml gentamycin for *E. coli* or 50 μg/ml rifampicin for *X. campestris*.

### 4.3 Protein expression and purification

Protein expression and purification were performed as described previously (Li *et al.*, 2017). To clone the Xcc *rpoN1* and Xcc *rpoN2* genes, genomic DNA extracted from *X. campestris* was used for PCR amplification with *Pfu* DNA polymerase using the primers listed in Table S2. PCR products were inserted into pET‐28b(+) to produce the plasmids pET‐*rpoN1* and pET‐*rpoN2*. The Xcc *rpoN1* and Xcc *rpoN2* genes were verified via nucleotide sequencing by Genscript. Xcc *rpoN1* and Xcc *rpoN2* with vector‐encoded His_6_‐tagged N‐termini were expressed in *E. coli* BL21 (DE3) and purified with Ni‐NTA agarose (Sigma‐Aldrich) using a nickel‐ion affinity column. Protein purity was monitored by sodium dodecyl sulphate polyacrylamide gel electrophoresis (SDS‐PAGE).

### 4.4 Deletion of Xcc *rpoN* genes and complementation

To disrupt the Xcc *rpoN1* and Xcc *rpoN2* genes, the pK18mobsacB‐borne in‐frame deletion suicide plasmids pK18‐Δ*rpoN1* and pK18‐Δ*rpoN2* were constructed. The 500‐bp DNA fragments flanking the Xcc *rpoN1* and Xcc *rpoN2* genes were amplified with *Pfu* DNA polymerase using *X. campestris* genomic DNA as a template, and either *rpoN1 Bam*HI and *rpoN1* up1 (for Up *rpoN1*), *rpoN1* down1 and *rpoN1 Hin*dIII (for Down *rpoN1*), *rpoN2 Bam*HI and *rpoN2* up1 (for Up *rpoN2*), or *rpoN2* down1 and *rpoN2 Hin*dIII (for Down *rpoN2*), as primers (Table S2). Fragments were purified and joined by overlap PCR. The fused fragment was digested with *Bam*HI and *Hin*dIII, and inserted into pK18mobscaB (Schafer *et al.*, 1994) to obtain the plasmids pK18‐Δ*rpoN1* and pK18‐Δ*rpoN2*. The resulting constructs were transferred into *X. campestris* by electroporation and kanamycin was used to select for integration of the nonreplicating plasmid into the recipient chromosome. A single‐crossover integrant colony was spread on NYG medium without kanamycin at 28 °C for 36 hr, and after appropriate dilution the culture was spread on NYG plates containing 15% sucrose. Colonies sensitive to kanamycin were screened by PCR using the primers listed in Table S2, and the Xcc *rpoN1* and Xcc *rpoN2* deletion strains (Δ*rpoN1* and Δ*rpoN2*) were obtained. For complementation of the Xcc *rpoN1* and Xcc *rpoN2* mutants, the coding regions of Xcc *rpoN1* and Xcc *rpoN2* were amplified by PCR and cloned into the versatile pBBR1MCS5 plasmid (Kovach *et al.*, 1995). The resulting plasmid was transferred into the *X. campestris* strain by electroporation. The Δ*rpoN1/N1* andΔ*rpoN2/N2* strains were obtained. The Δ*rpoN1N2* double‐mutant and complemented strains Δ*rpoN1/N2*, Δ*rpoN2/N1*, Δ*rpoN1N2/N1*, and Δ*rpoN1N2/N2* were obtained by the same method.

### 4.5 Pathogenicity assays

Plant inoculation and virulence assays were conducted as previously described (Cai *et al.*, 2017). In brief, 6‐week‐old plants of the cabbage cultivar *B. oleracea* 'Jingfeng No. 1' were used as host plants. The wild‐type strain Xc1 and sterile 10 mM MgCl_2_ were used as positive and negative controls, respectively. All bacterial strains were cultured overnight in NYG medium containing appropriate antibiotics. Cells were collected and washed with 10 mM MgCl_2_, and the cell densities were adjusted to OD_600_ = 0.1 before inoculation into plant leaves using sterile scissors. Lesion lengths were measured 10 days after inoculation on 20 leaves for each strain tested.

### 4.6 Quantitative reverse transcription PCR

RT‐qPCR was carried out as described in previous studies (Cui *et al.*, 2018). The bacterial cells were collected when the cell optical density (OD_600_) reached 1.0 in NYG medium. Total RNA was extracted using the TRIzol‐based method (Life Technologies). RNA quality control was performed by several steps: (a) RNA degradation degree and potential contamination were monitored on 1% agarose gels; (b) RNA purity (A_260_/A_280_, A_260_/A_230_) was checked using a NanoPhotometer spectrophotometer (IMPLEN); and (c) RNA integrity was measured using a Bioanalyzer 2,100 (Agilent). The primers used in this assay are listed in Table S2. Quantification of gene expression and melting curve analysis were completed using QuantStudio 6 Flex real‐time PCR system (Applied Biosystems), and TransStart Top Green qPCR SuperMix (TransGen Biotech) was used according to the manufacturer's instructions. As a control, RT‐qPCR was similarly applied to analyse *16S rDNA* gene expression. The relative expression levels of target genes were calculated using the 2^−ΔΔ*C*t^ method for comparative quantitation.

### 4.7 RNA‑Seq

The RNA‐Seq assay was performed as described previously (Yang *et al.*, 2017; Zhou *et al.*, 2017). Briefly, the wild‐type Xc1, Δ*rpoN1*, Δ*rpoN2*, and Δ*rpoN1N2* mutant strains were grown in NYG medium, and their cells were collected when the OD_600_ reached 1.0 based on the growth curve. The collected cells were used for RNA extraction by the TRIzol‐based method (Life Technologies), and RNA degradation and contamination were monitored on 1% agarose gels. Clustering and sequencing were performed by Genedenovo Biotechnology Co., Ltd (Guangzhou, Guangdong, China). To analyse the DEGs between the wild‐type Xc1, Δ*rpoN1*, Δ*rpoN2*, and Δ*rpoN1N2* mutant strains, the gene expression levels were further normalized using the fragments per kilobase of transcript per million (FPKM) mapped reads method to eliminate the influence of different gene lengths and amount of sequencing data on the calculation of gene expression. The edgeR package (http://www.r‐project.org/) was used to determine DEGs across samples with fold changes ≥2 and a false discovery rate‐adjusted *p* (*q* value) <.05. DEGs were then subjected to enrichment analysis of gene ontology (GO) functions and KEGG pathways, and *q* values were corrected using <.05 as the threshold.

### 4.8 Electrophoretic mobility gel shift assays

EMSAs were performed as described (Hirakawa *et al.*, 2015; Shao *et al.*, 2018). For RpoN gel shift assays, we used DNA fragments that included the pXCC *fliC* (408 bp) and pXCC *fliQ* (497 bp) promoter regions as probes. The probe DNA (50 ng) was mixed with protein in a 20 μl reaction mixture containing 10 mM Tris (pH 7.5), 50 mM KCl, 1 mM dithiothreitol, and 0.4% glycerol. After incubation for 25 min at 28 °C, the samples were electrophoresed on a 5% nondenaturing acrylamide gel in 0.5 × Tris‐borate‐EDTA (TBE) buffer at 4 °C. The gel was soaked in 10,000‐fold‐diluted SYBR Green I nucleic acid dye (Sangon Biotech), and the DNA was visualized at 300 nm.

### 4.9 Measurement of extracellular enzymatic activity and swimming motility

The relative activities of extracellular enzymes were assayed as described previously (Wei *et al.*, 2007; Yu *et al.*, 2016). Two microlitres of each *X. campestris* strain culture (OD_600_ ≈ 1.0) was spotted onto NYG agar plates containing 1% (wt/vol) skim milk (for protease), 0.5% (wt/vol) carboxymethylcellulose (for cellulase), or 0.1% (wt/vol) starch (for amylase) and incubated at 28 °C for 24–48 hr. The plates were stained where necessary as previously described (Wei *et al.*, 2007). The zones of clearance around the spots due to degradation of the substrate were photographed. Three plates were inoculated in each experiment, and each experiment was repeated three times. The relative enzymatic activity was indicated by the diameter of the clearance zone.

Swimming motility was determined on semisolid agar (0.3%). Bacteria were inoculated into the centres of NYG plates containing 0.3% agarose. The plates were incubated at 28 °C for 48 hr before the colony diameters were measured.

### 4.10 EPS formation assay

EPS production was measured as described previously (Yu *et al.*, 2016). Each *X. campestris* strain culture (2 ml, OD_600_ ≈ 1.0) was used to inoculate 100 ml of NYG medium containing 4% glucose in a 250 ml flask and kept at 28 °C with shaking at 180 rpm for 4 days. The EPS was precipitated from the culture supernatant by the addition of 4 volumes of ethanol. The pelleted EPS was washed with 70% ethanol, air dried, and weighed. Three flasks were inoculated in each experiment, and each experiment was repeated three times.

### 4.11 Biofilm formation assay

A biofilm formation assay was performed as described previously with some modifications (Wang *et al.*, 2018). Briefly, bacterial cells were cultured in NYG medium to a final OD_600_ of 1.0. Then, 3 ml of the cell suspension was added to sterilized polystyrene tubes. These tubes were kept in a humidified chamber at 28 °C for 7 days without shaking. The cultures were then moved and the tubes were washed three times in tap water. Biofilm formation on the tubes was visualized by staining with 0.1% CV, followed by washing three times in tap water. The CV‐stained biofilm in polystyrene tubes was dissolved in methanol–acetic acid–water (4:1:5, vol/vol/vol) and quantified by measuring the A_575_ using an 8,453 UV‐visible spectrophotometer (Agilent). The average of three replicates was used for quantitative measurement. The biofilm assays were repeated three times and showed similar results with three replicates each time.

### 4.12 Detection of DSF signal components in the *X. campestris* culture supernatant

The protocol for extraction and purification of DSF family components was described previously (Zhou *et al.*, 2015a). *X. campestris* strains were cultured in liquid medium for 36 hr and 50 ml of the bacterial supernatant was collected by centrifugation at 4,000 × g for 15 min at 4 °C. The pH of the supernatants was adjusted to 4.0 by adding hydrochloric acid prior to two extractions with an equal volume of ethyl acetate. The ethyl acetate fractions were collected, and the solvent was removed by rotary evaporation to dryness at 42 °C. The residue was dissolved in 100 µl of methanol. The crude extract was subjected to 0.22‐µm Mini‐star filtration, and the filtrate was concentrated to 100 µl. The extract (10 µl) was injected into a C18 reversed‐phase HPLC column (4.6 × 250 mm, Agilent Technologies, Inc.) and eluted with water in methanol (23:77 vol/vol, 0.1% formic acid) at a flow rate of 1 ml/min in an HPLC E2695 system (Waters) with a UV220 detector.

### 4.13 Analysis of fatty acid composition

Bacterial cultures were grown aerobically for 2–4 days. Cells were harvested and washed three times with sterile water. Fatty acid methyl esters were synthesized and extracted as described previously (Li *et al.*, 2017). Cellular lipids were saponified by addition of 2 ml of sodium hydroxide/methanol solution at 100 °C for 40 min with shaking (800 rpm). Fatty acids were methylated by the addition of 4 ml of hydrochloric acid/methanol solution at 80 °C for 20 min and cooled to less than 20 °C. Fatty acid methyl esters were obtained by three extractions with 1 ml of petroleum ether. The solvent was removed under a stream of nitrogen, and the residue was dissolved in 100 µl of hexane. The crude extract was filtered with 0.22‐µm Mini‐star units, and 2 µl of extract was analysed by GC‐MS. Samples were analysed with a GC‐MS system (Agilent 5975c) with a DB 5MS chromatographic column. The oven temperature was held at 100 °C for 5 min, changed at 10 °C/min to 200 °C and held for 5 min, then changed at 10 °C/min to 250 °C and held for 5 min. Electron impact ionization (EI+, 70 eV) was used for all samples. Mass spectrometry was carried out at 1 s/scan, *m*/*z* 35–500, and 1 kV, and the data were analysed by the NIST 08 database.

### 4.14 Statistical analyses

The experimental datasets were subjected to analyses of variance using GraphPad Prism 7.0. The significance of the treatment effects was determined by the *F* value (*p* = .05). If a significant *F* value was obtained, separation of means was accomplished by Fisher's protected least significant difference at *p* ≤ .05.

## CONFLICT OF INTEREST

The authors declare no competing financial interests.

## Data Availability

The data that support the findings of this study are available from the corresponding author upon reasonable request.
