# Supplementary material for: RpoN1 and RpoN2 play different regulatory roles in virulence traits, flagellar biosynthesis, and basal metabolism in Xanthomonas campestris
Source: Mol Plant Pathol. 2020 Apr 13;21(7):907–22. doi: 10.1111/mpp.12938 (PMC7280030; doi:10.1111/mpp.12938)
Supplement: Supplementary file 1 [file MPP-21-907-s001.docx]

**Fig. S1**

**
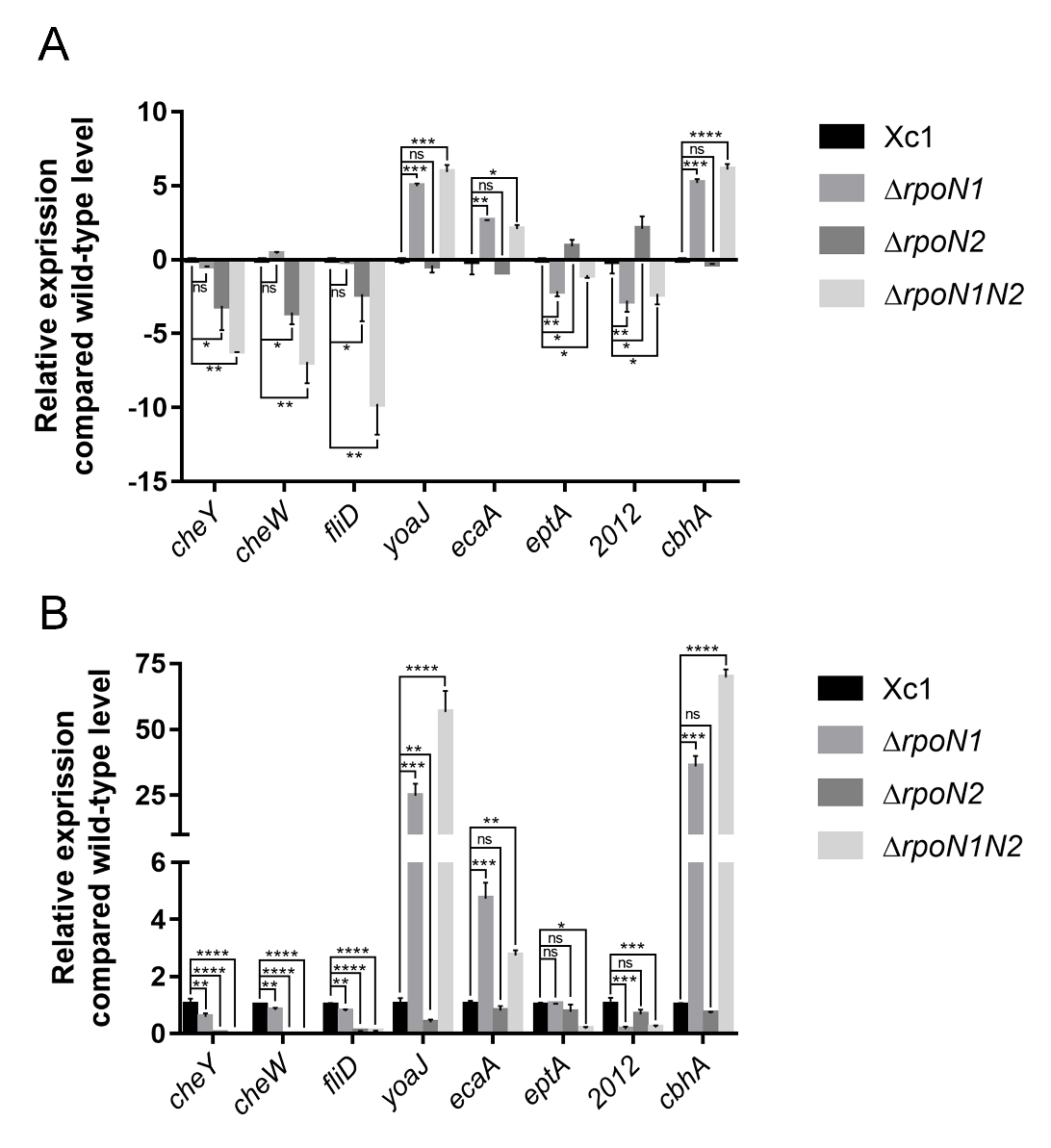
**

**Fig. S1. Differential expression of ten randomly selected genes as determined by RNA-Seq (A) and qRT-PCR (B)**. *cheY*, chemotaxis response regulator; *cheW*, chemotaxis protein; *fliD*, flagellar protein*;* *yoaJ*, glycosidase; *ecaA*, carbonic anhydrase; *eptA*, phosphoethanolamine transferase; XCC2012, NAD(P)-dependent oxidoreductase; *cbhA,* 1,4-beta-cellobiosidase. The qRT-PCR data were normalized to 16s rDNA levels and are presented as the fold change with respect to the wild type expression level of each gene. Error bars, means ± standard deviations (n = 3). ∗ P < 0.05, ∗∗ P < 0.01, ∗∗∗ P < 0.001, ∗∗∗∗ P < 0.0001, assessed by one-way ANOVA. All experiments were repeated three times with similar results.
