# Supplementary material for: RpoN1 and RpoN2 play different regulatory roles in virulence traits, flagellar biosynthesis, and basal metabolism in Xanthomonas campestris
Source: Mol Plant Pathol. 2020 Apr 13;21(7):907–22. doi: 10.1111/mpp.12938 (PMC7280030; doi:10.1111/mpp.12938)
Supplement: Supplementary file 2 [file MPP-21-907-s002.docx]

**Fig. S2**

**
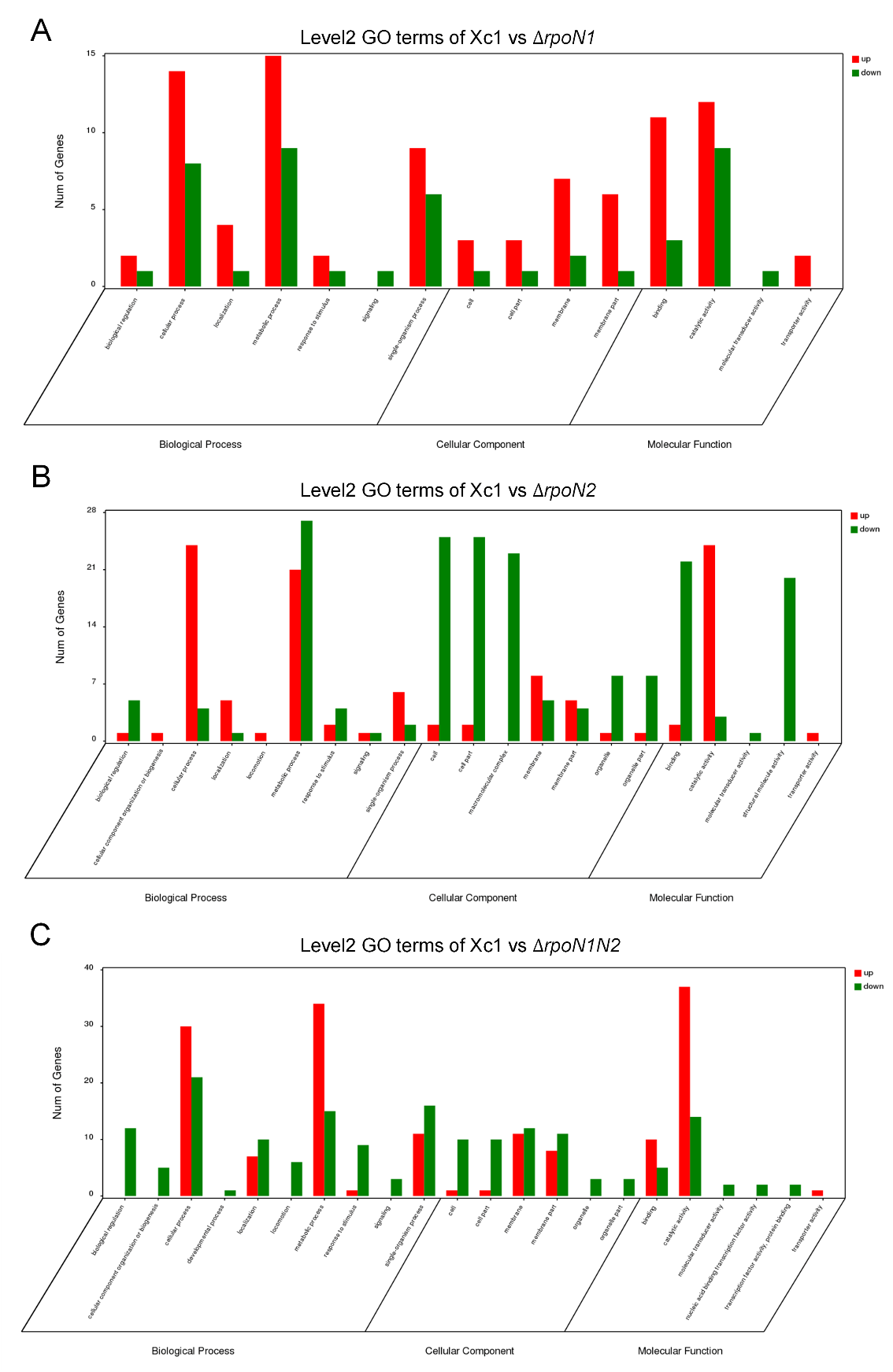
**

**Fig. S2. Differential gene expression profiles among Xc1, Δ*rpoN1*, Δ*rpoN2*, and Δ*rpoN1N2* as measured by RNA-Seq (log2-fold change ≥ 1) (A-C)** GO term enrichment analysis of DEGs: Xc1 vs Δ*rpoN1*, Xc1 vs Δ*rpoN2* and Xc1 vs Δ*rpoN1N2*.
