# Supplementary material for: RpoN1 and RpoN2 play different regulatory roles in virulence traits, flagellar biosynthesis, and basal metabolism in Xanthomonas campestris
Source: Mol Plant Pathol. 2020 Apr 13;21(7):907–22. doi: 10.1111/mpp.12938 (PMC7280030; doi:10.1111/mpp.12938)
Supplement: Supplementary file 3 [file MPP-21-907-s003.docx]

**Fig. S3**

**
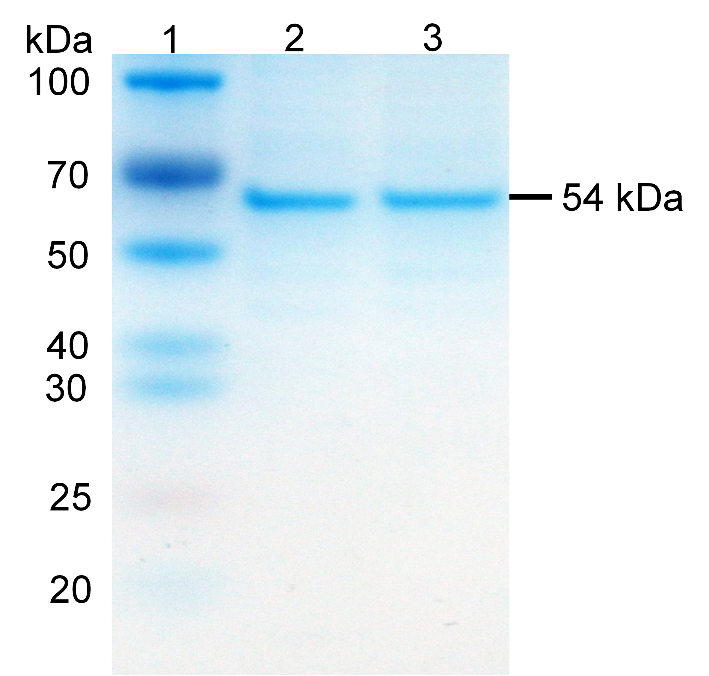
**

**Fig. S3. Purification of RpoN1 and RpoN2 by native nickel-chelate chromatography.** The purified protein was analysed by 12% SDS-PAGE. Lane 1, molecular mass markers; lane 2, RpoN1 protein; lane 3, RpoN2 protein.
