# Supplementary material for: RpoN1 and RpoN2 play different regulatory roles in virulence traits, flagellar biosynthesis, and basal metabolism in Xanthomonas campestris
Source: Mol Plant Pathol. 2020 Apr 13;21(7):907–22. doi: 10.1111/mpp.12938 (PMC7280030; doi:10.1111/mpp.12938)
Supplement: Supplementary file 4 [file MPP-21-907-s004.docx]

**Fig. S4**

**
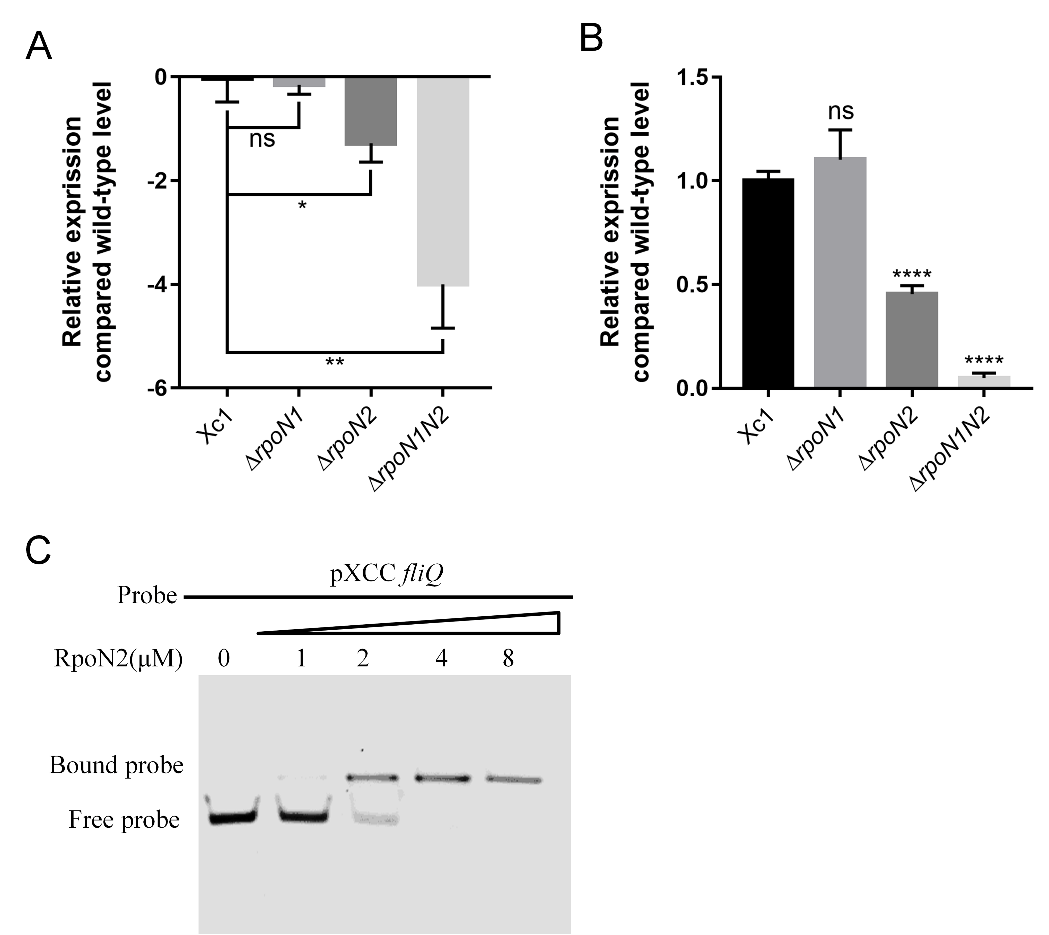
**

**Fig. S4. Gel shift assay showing that RpoN2 directly regulates XCC *fliQ*. (A)** Relative expression of XCC *fliQ* as determined by RNA-Seq. **(B)** Relative expression of XCC *fliQ* as determined by qRT-PCR. **(C)** Gel shift assay showing that RpoN2 directly regulates XCC *fliQ*. RpoN1 (0, 1, 2, 4 or 8 μM) was added to the reaction mixtures containing 50 ng of probe DNA, and the reaction mixtures were separated on polyacrylamide gels. Error bars, means ± standard deviations (n = 3). ∗ P < 0.05, ∗∗ P < 0.01, ∗∗∗∗ P < 0.0001, assessed by one-way ANOVA. All experiments were repeated three times with similar results.
