# Supplementary material for: RpoN1 and RpoN2 play different regulatory roles in virulence traits, flagellar biosynthesis, and basal metabolism in Xanthomonas campestris
Source: Mol Plant Pathol. 2020 Apr 13;21(7):907–22. doi: 10.1111/mpp.12938 (PMC7280030; doi:10.1111/mpp.12938)
Supplement: Supplementary file 5 [file MPP-21-907-s005.docx]

**Fig. S5**

**
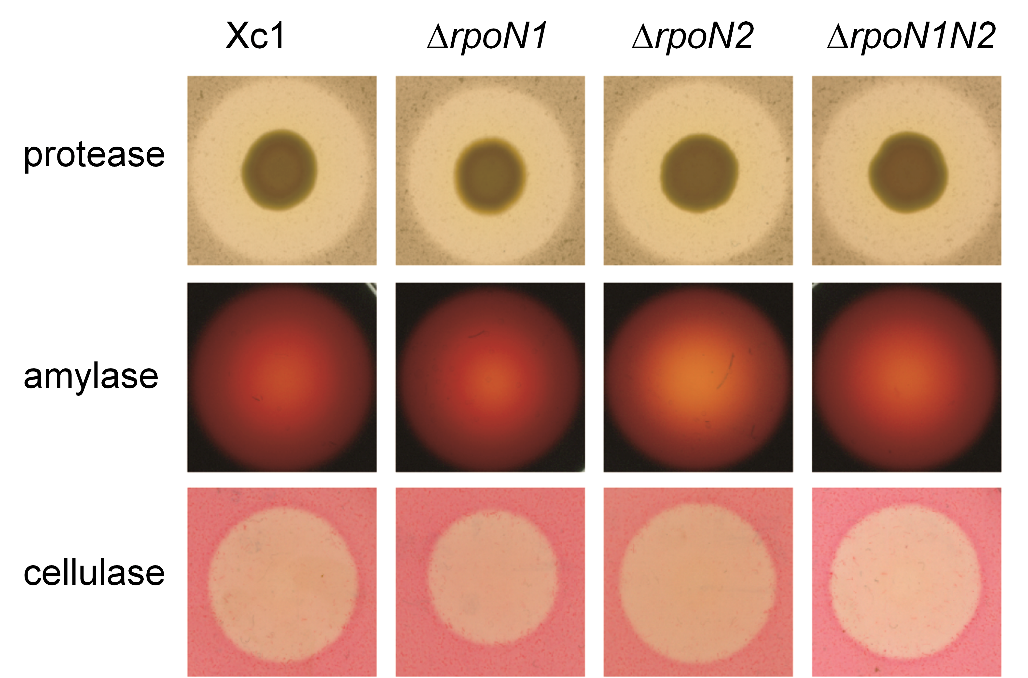
**

**Fig. S5. Relative activity of extracellular enzymes produced by *X. campestris* strains in NYG medium.** Error bars indicate standard deviations (n = 3). All experiments were repeated three times with similar results.
