# Supplementary material for: RpoN1 and RpoN2 play different regulatory roles in virulence traits, flagellar biosynthesis, and basal metabolism in Xanthomonas campestris
Source: Mol Plant Pathol. 2020 Apr 13;21(7):907–22. doi: 10.1111/mpp.12938 (PMC7280030; doi:10.1111/mpp.12938)
Supplement: Supplementary file 7 [file MPP-21-907-s007.docx]

**Table S1. Bacterial strains and plasmids used in this study**

| Strains | | Relevant characteristics | | References |
| --- | --- | --- | --- | --- |
| *E.coli* |  | |  | |
| BL21(DE3) | F^-^ *dcm* *omp T* *hsdS*(r_B_^-^m_B_^-^) *gal* (λDE3) | | Lab collection | |
| DH5α | F^-^ *deoR* *endA1 gyrA96 hsdR17*(r_K_^-^m_K_^+^) *recA1* *relA1* *supE*44 *thi-1* Δ(*lacZYA-argF*)*U*169(φ80*lacZ*ΔM15) | | Lab collection | |
| *X. campestris* | | |  | |
| Xc1 | Rif^R^, Wild-type strain | | Lab stock | |
| Δ*rpoN1* | Rif^R^, The XCC *rpoN1* in-frame deletion mutant of strain Xc1 | | This study | |
| Δ*rpoN2* | Rif^R^, The XCC *rpoN2* in-frame deletion mutant of strain Xc1 | | This study | |
| Δ*rpoN1N2* | Rif^R^, The XCC *rpoN1* and XCC *rpoN2* in-frame deletion mutant of strain Xc1 | | This study | |
| Δ*rpoN1/N1* | Rif^R^, Gm^r^, The *rpoN1* in-frame deletion mutant harboring the *rpoN1* expression plasmid pBBR1- *rpoN1*. | | This study | |
| Δ*rpoN1/N2* | Rif^R^, Gm^r^, The *rpoN1* in-frame deletion mutant harboring the *rpoN2* expression plasmid pBBR1-*rpoN2*. | | This study | |
| Δ*rpoN2/N1* | Rif^R^, Gm^r^, The *rpoN2* in-frame deletion mutant harboring the *rpoN1* expression plasmid pBBR1-*rpoN1*. | | This study | |
| Δ*rpoN2/N2* | Rif^R^, Gm^r^, The *rpoN2* in-frame deletion mutant harboring the *rpoN2* expression plasmid pBBR1-*rpoN2*. | | This study | |
| Δ*rpoN1N2/N1* | Rif^R^, Gm^r^, The *rpoN1* and *rpoN2* double mutant strain harboring the *rpoN1* expression plasmid pBBR1-*rpoN1*. | | This study | |
| Δ*rpoN1N2/N2* | Rif^R^, Gm^r^, The *rpoN1* and *rpoN2* double mutant strain harboring the *rpoN2* expression plasmid pBBR1-*rpoN2*. | | This study | |
| Δ*rpfC* | Rif^R^, The XCC *rpfC* in-frame deletion mutant of strain Xc1 | | (Zhou et al., 2015) | |
| Δ*rpfC*Δ*rpoN1* | Rif^R^, The XCC *rpoN1* in-frame deletion mutant of strain Δ*rpfC* | | This study | |
| Δ*rpfC*Δ*rpoN2* | Rif^R^, The XCC *rpoN2* in-frame deletion mutant of strain Δ*rpfC* | | This study | |
| Δ*rpfC*Δ*rpoN1N2* | Rif^R^, The XCC *rpoN1* and *rpoN2* in-frame deletion mutant of strain Δ*rpfC* | | This study | |
|  |  | |  | |
| Plasmids |  | |  | |
| pET28(b) | Km^R^, T7 promoter-based expression vector | | Lab collection | |
| pK18mobsacB | Km^R^, *sacB*-based gene replacement vector | | (Schafer et al., 1994) | |
| pBBR1MCS5 | Gm^R^, Broad host range cloning vector. | | (Kovach et al., 1995) | |
| pET-*rpoN1* | Km^R^, XCC *rpoN1* in pET-28b | | This study | |
| pET-*rpoN2* | Km^R^, XCC *rpoN2* in pET-28b | | This study | |
| pBBR1-*rpoN1* | Gm^R^, XCC *rpoN1* in pBBR1MCS5 | | This study | |
| pBBR1-*rpoN2* | Gm^R^, XCC *rpoN2* in pBBR1MCS5 | | This study | |
| pK18-Δ*rpoN1* | Km^R^, XCC *rpoN1* in-fame deletion fragment inserted to pK18mobscaB vector between EcoRI/HindIII sites | | This study | |
| pK18-Δ*rpoN2* | Km^R^, XCC *rpoN2* in-fame deletion fragment inserted to pK18mobscaB vector between EcoRI/HindIII sites | | This study | |

Km^R^, Gm^R^, Rif^R^: Kanamycin, Gentamicin, Rifampicin resistance, respectively.
