# Supplementary material for: RpoN1 and RpoN2 play different regulatory roles in virulence traits, flagellar biosynthesis, and basal metabolism in Xanthomonas campestris
Source: Mol Plant Pathol. 2020 Apr 13;21(7):907–22. doi: 10.1111/mpp.12938 (PMC7280030; doi:10.1111/mpp.12938)
Supplement: Supplementary file 8 [file MPP-21-907-s008.docx]

**Table S2. Sequences of the PCR primers used in this work**

| Primer name | Primer sequence (5’ to 3’) | Digestion sites ^a^ |
| --- | --- | --- |
| **For deletion** |  |  |
| *rpoN1* *Bam*HⅠ | aattGGATCCtgttgatcaccgatcacaacg | *Bam*HⅠ |
| *rpoN1* up1 | atcgatcaggcgccggatcatgttgtcgtcatcgtcgaagct |  |
| *rpoN1* down1 | agcttcgacgatgacgacaacatgatccggcgcctgatcgat |  |
| *rpoN1* *Hin*dIII | tataAAGCTTagcttgtcgaccaacaggtc | *Hin*dIII |
| *rpoN2* *Bam*HⅠ | aattGGATCCaagcatcacaacgagctggtc | *Bam*HⅠ |
| *rpoN2* up1 | gaaatgcttcagttcgaaggtgatggtgctttcgggcaactcgt |  |
| *rpoN2* down1 | acgagttgcccgaaagcaccatcaccttcgaactgaagcatttc |  |
| *rpoN2* *Hin*dIII | tataAAGCTTatcgtcctgcgtcttgatcag | *Hin*dIII |
| **For in trans expression** |  |  |
| *rpoN1*-F | atccAAGCTTcaggccaggagaaagaactg | *Hin*dIII |
| *rpoN1*-R | tgcTCTAGAggtggccatacgtctcgata | *Xba* I |
| *rpoN2*-F | atccAAGCTTtgagcccgcagatctcttc | *Hin*dIII |
| *rpoN2*-R | tgcTCTAGAcactgcccacgatattgagc | *Xba* I |
| **For protein expression** |  |  |
| 28b-*rpoN1* P1 | aattATGCATatgaaagcccggctccagacatc | *Nde* I |
| 28b-*rpoN1* P2 | aattAAGCTTgttgcggtggttgcaacgtcag | *Hin*dIII |
| 28b-*rpoN2* P1 | aattATGCATatgaagacgaccatctctg | *Nde* I |
| 28b-*rpoN2* P2 | aattAAGCTTgagtttgctcatagtagtc | *Hin*dIII |
| **For RT-PCR** |  |  |
| RT-*filC*-F | ccaaccagaccagcttcaac |  |
| RT-*filC*-R | tgaaggccagagtgataccg |  |
| RT-*filQ*-F | gattgccggaccgatgttg |  |
| RT-*filQ*-R | aactccaccagatgtcccag |  |
| RT-*leuA*-F | ctgcatgtgttcctgtccac |  |
| RT-*leuA*-R | cggccaggaaatcttcttcg |  |
| RT-*rpfB*-F | caaccacctgatcagcaacc |  |
| RT-*rpfB*-R | agggtcatcttcaacgacga |  |
| RT-*cheY*-F | agatggtctctttcgccctc |  |
| RT-*cheY*-R | gccttgccttcggatttctt |  |
| RT-*cheW*-F | gaaatccgcggctatgactc |  |
| RT-*cheW*-R | gatcatcacggtgaaggcg |  |
| RT-*filD*-F | ccgacaccaatgcctacaag |  |
| RT-*filD*-R | gtccacggtgatgcttttgt |  |
| RT-*yoaJ*-F | tcaggattggaaccgggtag |  |
| RT-*yoaJ*-R | caacttcggcatcaacacca |  |
| RT-*ecaA*-F | ccgaactggccaaggaaaat |  |
| RT-*ecaA*-R | cgctgggatgatggaaatgc |  |
| RT-*eptA*-F | acttcaaatccgattcgccg |  |
| RT-*eptA*-R | gcatgtccgggaagttgatc |  |
| RT-*XCC2012*-F | gttatgaacaccacggccac |  |
| RT-*XCC2012*-R | tcgccgagtttgtagaggtt |  |
| RT-*cbhA*-F | tccatgccactcccaatctt |  |
| RT-*cbhA*-R | gtgtaattggcggtgttggt |  |
| RT-2574 F | gacccggagtactacaccag |  |
| RT-2574 R | agatcgggttataggccagc |  |
| RT-16S rRNA-F | taaagcgtgcgtaggtggtg |  |
| RT-16S rRNA-R | tttcgtgcctcagtgtcagt |  |
| **For EMSA** |  |  |
| pXCC *fliC* up | gctgttcaatctgatccgct |  |
| pXCC *fliC* dn | tcgaactgttggtgttgagg |  |
| pXCC *fliQ* up | acggccgagaacagctagta |  |
| pXCC *fliQ* dn | cacaccaatcacgacaccaa |  |
| pXCC *leuA* up | cggtggttatctcaggtcca |  |
| pXCC *leuA* dn | tcgttgctcattgaaaatc |  |
| pXCC *rpfB* up | tggtgacctggtagtgctg |  |
| pXCC *rpfB* dn | gccggataactttgcaacca |  |
| pXCC 2574 up | cagtgtatggagacgaccgt |  |
| pXCC 2574 dn | tacgaatcacaagggctcca |  |

*^a^* Underlined, restriction endonuclease sites.
