# Supplementary material for: RpoN1 and RpoN2 play different regulatory roles in virulence traits, flagellar biosynthesis, and basal metabolism in Xanthomonas campestris
Source: Mol Plant Pathol. 2020 Apr 13;21(7):907–22. doi: 10.1111/mpp.12938 (PMC7280030; doi:10.1111/mpp.12938)
Supplement: Supplementary file 9 [file MPP-21-907-s009.docx]

**Table S3. List of genes differentially expressed** **in the Δ*rpoN1* mutant compared to the wild-type strain (log_2_ fold change ≥ 1).**

| Gene name | log2 fold change (Δ*rpoN1* / Xc1) | Gene Description |
| --- | --- | --- |
| XCC0026 | -1.69971 | cellulase |
| XCC0029 | 2.040515 | alcohol dehydrogenase |
| XCC0034 | 2.248364 | DNA topoisomerase |
| XCC0035 | 2.287791 | SAM-dependent methyltransferase |
| XCC0036 | 2.829797 | hypothetical protein |
| XCC0052 | 1.142458 | avirulence protein |
| XCC0069 | -3.84645 | dihydrofolate reductase |
| XCC0070 | 1.847647 | CsbD family protein |
| XCC0072 | -1.07538 | hypothetical protein |
| XCC0077 | 1.583238 | NAD-dependent dehydratase |
| XCC0080 | -1.68443 | esterase |
| XCC0081 | -1.77852 | attachment protein |
| XCC0091 | -1.05437 | ATPase AAA |
| XCC0102 | 1.325112 | acetaldehyde dehydrogenase |
| XCC0104 | 1.525691 | Ku protein |
| XCC0113 | 1.676837 | hypothetical protein |
| XCC0114 | 1.271267 | transcriptional regulator |
| XCC0119 | -2.44927 | TonB-dependent receptor |
| XCC0120 | -3.81081 | TonB-dependent receptor |
| XCC0121 | -2.40656 | pectin methylesterase |
| XCC0122 | -2.81138 | pectate lyase |
| XCC0141 | 1.754593 | L-fucose dehydrogenase |
| XCC0142 | -2.03924 | lysophospholipase |
| XCC0143 | -1.97592 | carboxylesterase |
| XCC0144 | -2.3347 | xylanase |
| XCC0145 | -2.62085 | 2-keto-4-pentenoate hydratase |
| XCC0146 | 2.844191 | C4-dicarboxylate ABC transporter |
| XCC0147 | 2.712508 | C4-dicarboxylate ABC transporter |
| XCC0148 | 1.683549 | C4-dicarboxylate transport protein |
| XCC0149 | 1.409306 | xylosidase/arabinosidase |
| XCC0151 | -1.71336 | 5-keto-4-deoxyuronate isomerase |
| XCC0152 | -1.55127 | 2-deoxy-D-gluconate 3-dehydrogenase |
| XCC0153 | -1.17141 | sugar-phosphate isomerase |
| XCC0154 | -1.58249 | rhamnogalacturonan acetylesterase |
| XCC0167 | 1.714498 | ergothioneine biosynthesis protein EgtB |
| XCC0168 | 1.637468 | L-histidine N(alpha)-methyltransferase |
| XCC0206 | 1.938119 | pyruvate oxidase |
| XCC0247 | -1.02838 | AcrR family transcriptional regulator |
| XCC0251 | 1.829303 | membrane protein |
| XCC0274 | 1.31733 | conserved small membrane protein |
| XCC0281 | 3.704398 | DUF4440 domain-containing protein |
| XCC0284 | 1.918316 | Zn-dependent hydrolase |
| XCC0324 | 1.920423 | methyl-accepting chemotaxis protein |
| XCC0353 | 1.99372 | 3-alpha-hydroxysteroid dehydrogenase |
| XCC0354 | 2.063086 | benzaldehyde dehydrogenase |
| XCC0357 | 1.828877 | hypothetical protein |
| XCC0360 | 1.209894 | glycerol-3-phosphate dehydrogenase |
| XCC0362 | 1.154317 | diguanylate cyclase |
| XCC0363 | 1.376293 | (2Fe-2S)-binding protein |
| XCC0364 | 1.824019 | 3-oxoadipate--succinyl-CoA transferase subunit A |
| XCC0365 | 1.775555 | 3-oxoadipate--succinyl-CoA transferase subunit B |
| XCC0366 | 1.843542 | 3-oxoadipyl-CoA thiolase |
| XCC0367 | 1.06376 | protocatechuate 3,4-dioxygenase subunit beta |
| XCC0368 | 1.191728 | protocatechuate 3,4-dioxygenase subunit alpha |
| XCC0393 | -3 | transposase, partial |
| XCC0394 | -1.29535 | TonB-dependent siderophore receptor |
| XCC0401 | 1.111413 | hypothetical protein |
| XCC0404 | 2.288845 | ABC transporter substrate-binding protein |
| XCC0408 | 1.159 | starch synthase |
| XCC0418 | 2.172975 | VirK protein |
| XCC0436 | -1.04752 | 4-hydroxyphenylpyruvate dioxygenase |
| XCC0438 | -1.11103 | homogentisate 1,2-dioxygenase |
| XCC0450 | 1.612912 | putative exported protein |
| XCC0458 | 1.377572 | diacylglycerol kinase-like enzyme |
| XCC0511 | 2.847531 | hypothetical protein |
| XCC0521 | 1.925596 | ribonuclease |
| XCC0531 | -1.47443 | membrane protein |
| XCC0537 | 1.280256 | putative exported protein |
| XCC0538 | 1.78544 | secreted protein |
| XCC0546 | 2.08327 | secreted protein |
| XCC0572 | 1.128843 | Putative secreted protein |
| XCC0644 | 3.58965 | pectate lyase |
| XCC0645 | 2.190011 | pectate lyase |
| XCC0658 | 1.015237 | membrane protein |
| XCC0659 | 1.202299 | protease |
| XCC0666 | 1.18588 | type II secretion system protein K |
| XCC0684 | 1.513971 | serine protease |
| XCC0694 | -1.1404 | putative exported protein |
| XCC0703 | 1.156947 | potassium-transporting ATPase subunit B |
| XCC0706 | 1.042519 | DNA-binding response regulator |
| XCC0741 | 1.196631 | hypothetical protein |
| XCC0756 | 1.27742 | membrane protein |
| XCC0767 | 1.424811 | hemin transporter HemP |
| XCC0768 | 1.352182 | sugar transporter |
| XCC0779 | 1.300392 | DNA-binding response regulator |
| XCC0780 | 1.360701 | sensor histidine kinase |
| XCC0783 | 1.2462 | LemA family protein |
| XCC0813 | -1.16096 | hypothetical protein |
| XCC0814 | -1.66345 | gluconolactonase |
| XCC0815 | -1.08095 | glucose-fructose oxidoreductase |
| XCC0819 | 1.921612 | multidrug DMT transporter permease |
| XCC0843 | 2.921913 | alpha/beta hydrolase |
| XCC0851 | 2.57022 | protease |
| XCC0852 | 2.679767 | peptidase S8 |
| XCC0857 | 1.991901 | xylanase |
| XCC0923 | 1.206954 | membrane protein |
| XCC1019 | 1.187272 | acyl carrier protein |
| XCC1029 | 2.438292 | lipoprotein |
| XCC1031 | -2.80919 | methylisocitrate lyase |
| XCC1032 | -2.16242 | 2-methylcitrate synthase |
| XCC1033 | -2.43998 | Fe/S-dependent 2-methylisocitrate dehydratase AcnD |
| XCC1045 | 1.927107 | bacterioferritin |
| XCC1046 | 2.017007 | peroxiredoxin |
| XCC1074 | 1.109119 | TetR family transcriptional regulator |
| XCC1075 | 1.248461 | histidine phosphatase family protein |
| XCC1076 | 1.896907 | ADP-dependent (S)-NAD(P)H-hydrate dehydratase |
| XCC1077 | -1.04542 | glycosyl hydrolase |
| XCC1078 | -3.58496 | glycosyl hydrolase |
| XCC1081 | -1.39391 | sugar hydrolase |
| XCC1082 | -1.81047 | short-chain dehydrogenase |
| XCC1097 | 3.104996 | DNA-binding protein |
| XCC1117 | -2.10469 | phosphoethanolamine transferase |
| XCC1119 | -1.85635 | membrane protein |
| XCC1179 | -1.22451 | TonB-dependent receptor |
| XCC1186 | -1.15957 | PAS domain-containing sensor histidine kinase |
| XCC1187 | -1.20608 | response regulator |
| XCC1188 | 2.01415 | endo-1,3-beta-glucanase |
| XCC1294 | 2.091922 | GGDEF domain-containing protein |
| XCC1295 | 2.129085 | membrane protein |
| XCC1304 | -1.30791 | phage/conjugal plasmid C-4 type zinc finger protein, TraR family |
| XCC1306 | -3.37744 | RNA polymerase sigma factor |
| XCC1307 | -2.59378 | hypothetical protein |
| XCC1310 | -1.11132 | peptidyl-prolyl cis-trans isomerase |
| XCC1315 | 2.181704 | alkene reductase |
| XCC1319 | -2.27455 | 2'-5' RNA ligase |
| XCC1323 | 1.161456 | membrane protein |
| XCC1331 | 1.309906 | dehydrogenase |
| XCC1333 | 1.197133 | hypothetical protein |
| XCC1340 | 4.430572 | TonB-dependent receptor |
| XCC1343 | 2.293814 | S-methylmethionine permease |
| XCC1344 | 1.66157 | homocysteine S-methyltransferase |
| XCC1390 | 1.187528 | Nitrous oxidase accessory protein |
| XCC1404 | -1.21869 | glycosyl hydrolase |
| XCC1410 | 1.214687 | DUF3298 domain-containing protein |
| XCC1412 | -1.11196 | dipeptidyl carboxypeptidase II |
| XCC1420 | 1.617436 | phospholipase |
| XCC1422 | 1.018919 | cold-shock protein |
| XCC1433 | 1.687326 | NADPH-dependent oxidoreductase |
| XCC1443 | 1.227307 | GGDEF domain-containing protein |
| XCC1444 | 3.232661 | hypothetical protein |
| XCC1455 | 1.061041 | transposase |
| XCC1461 | 1.810623 | phage-related protein |
| XCC1481 | 1.270543 | DNA-binding transcriptional regulator OxyR |
| XCC1482 | 1.225801 | ABC transporter ATP-binding protein |
| XCC1523 | 1.124155 | phosphate transport system protein |
| XCC1538 | 1.301388 | lipoprotein |
| XCC1539 | 1.259054 | N-acetylmuramoyl-L-alanine amidase |
| XCC1575 | 1.823851 | membrane-bound PQQ-dependent dehydrogenase, glucose/quinate/shikimate family |
| XCC1577 | -2.04204 | urocanate hydratase |
| XCC1578 | -1.92336 | N-formylglutamate deformylase |
| XCC1579 | -1.80044 | histidine ammonia-lyase |
| XCC1580 | -1.45945 | formimidoylglutamate deiminase |
| XCC1581 | -1.32077 | imidazolonepropionase |
| XCC1615 | 1.127143 | hypothetical protein |
| XCC1616 | 4.444789 | hypothetical protein |
| XCC1617 | 4.360053 | hypothetical protein |
| XCC1634 | 11.5068 | ISxac3 transposase |
| XCC1636 | 4.416363 | IS5/IS1182 family transposase |
| XCC1648 | -2.46716 | hypothetical protein |
| XCC1670 | 2.754423 | dTDP-6-deoxy-3,4-keto-hexulose isomerase |
| XCC1688 | 1.283833 | exod protein |
| XCC1728 | 1.669997 | quercetin 2,3-dioxygenase |
| XCC1778 | 1.104236 | mannan endo-1,4-beta-mannosidase |
| XCC1786 | 1.031004 | EamA family transporter |
| XCC1800 | 1.006999 | bifunctional aspartokinase I/homoserine dehydrogenase I |
| XCC1802 | 1.160341 | ethyl tert-butyl ether degradation protein EthD |
| XCC1807 | -1.37938 | DNA-binding transcriptional regulator |
| XCC1875 | 4.125516 | methyl-accepting chemotaxis protein |
| XCC1884 | 2.316591 | methyl-accepting chemotaxis protein |
| XCC1912 | 1.079322 | GGDEF domain-containing protein |
| XCC1961 | 1.26791 | hypothetical protein |
| XCC2002 | 1.24519 | hypothetical protein |
| XCC2012 | -2.6681 | NAD(P)-dependent oxidoreductase |
| XCC2014 | -1.9324 | NAD-dependent epimerase |
| XCC2021 | -1.45851 | chemotaxis protein-glutamate methylesterase |
| XCC2023 | -1.48608 | diguanylate phosphodiesterase |
| XCC2024 | -5.81442 | YapH protein |
| XCC2031 | 1.566233 | membrane protein |
| XCC2032 | 1.129109 | sorbosone dehydrogenase |
| XCC2034 | -1.31805 | succinoglycan biosynthesis protein |
| XCC2035 | -1.01379 | peptidase |
| XCC2047 | 3.89154 | methyl-accepting chemotaxis protein |
| XCC2059 | -1.57809 | single-stranded DNA binding protein |
| XCC2074 | -1.57809 | single-stranded DNA binding protein |
| XCC2135 | -1.00594 | ABC transporter ATP-binding protein |
| XCC2150 | 2.809587 | hypothetical protein |
| XCC2156 | 2.086367 | oxidoreductase |
| XCC2180 | 1.01178 | DNA-binding response regulator |
| XCC2208 | -1.37793 | membrane protein |
| XCC2216 | 1.307379 | glutamine cyclotransferase |
| XCC2223 | 1.142273 | cytochrome c-type biogenesis protein CcmE 2 |
| XCC2224 | 1.133861 | c-type cytochrome biogenesis protein CcmF |
| XCC2227 | 1.085041 | cytochrome c biogenesis protein |
| XCC2231 | 1.243502 | thiol reductant ABC exporter subunit CydD |
| XCC2262 | 1.627858 | hypothetical protein |
| XCC2265 | -2.07053 | pectinesterase |
| XCC2312 | 2.510177 | RecA/RadA recombinase |
| XCC2336 | 1.079428 | succinate-semialdehyde dehydrogenase |
| XCC2339 | 1.280928 | Putrescine ABC transporter permease |
| XCC2343 | 1.225852 | transporter |
| XCC2349 | 1.11038 | FAD-dependent oxidoreductase |
| XCC2363 | 1.127614 | isochorismatase |
| XCC2382 | 2.18516 | hypothetical protein |
| XCC2453 | -1.29109 | GumC protein |
| XCC2509 | -1.03387 | tRNA pseudouridine(55) synthase TruB |
| XCC2562 | 1.06925 | rhomboid family intramembrane serine protease |
| XCC2574 | -1.29185 | peptidase |
| XCC2575 | 2.51188 | peptidase |
| XCC2601 | 2.475795 | peptidase |
| XCC2602 | 2.474129 | peptidase M35 |
| XCC2628 | 1.638111 | transcriptional regulator |
| XCC2629 | 1.947733 | hypothetical protein |
| XCC2630 | 2.029279 | hypothetical protein |
| XCC2650 | 1.571601 | membrane protein |
| XCC2664 | 1.605282 | formate dehydrogenase subunit alpha |
| XCC2666 | 1.294846 | serine protease |
| XCC2667 | 1.351079 | serine protease |
| XCC2682 | 1.520823 | multidrug efflux RND transporter permease subunit |
| XCC2683 | 1.872888 | MexE family multidrug efflux RND transporter periplasmic adaptor subunit |
| XCC2694 | 1.002868 | two-component sensor histidine kinase |
| XCC2710 | -1.29857 | peptidase |
| XCC2713 | 2.697265 | endonuclease V |
| XCC2725 | 1.866572 | cytidylyltransferase-like enzyme |
| XCC2745 | 2.117207 | peroxiredoxin |
| XCC2752 | 3.363748 | DUF72 protein |
| XCC2760 | -1.23726 | DUF3861 domain-containing protein |
| XCC2775 | 2.026928 | membrane protein |
| XCC2778 | 1.113532 | thiamine biosynthesis protein ApbE |
| XCC2781 | 1.317285 | competence protein ComEA |
| XCC2815 | 1.342651 | pectate lyase |
| XCC2818 | 1.536524 | LuxR family transcriptional regulator |
| XCC2820 | 1.788474 | endoproteinase ArgC, partial |
| XCC2821 | 1.796515 | serine protease |
| XCC2830 | -1.22765 | peptidase M1 |
| XCC2845 | 1.64655 | MFS transporter |
| XCC2848 | 1.120112 | hybrid sensor histidine kinase/response regulator |
| XCC2854 | 1.074659 | hydrolase |
| XCC2861 | 2.906498 | membrane protein |
| XCC2943 | -3.2094 | glucokinase |
| XCC2944 | -3.97225 | TonB-dependent receptor |
| XCC2956 | 1.174153 | chloride channel protein |
| XCC2958 | 1.504965 | response regulator |
| XCC2959 | 1.158645 | histidine kinase |
| XCC3044 | 1.383264 | glyoxalase |
| XCC3049 | 2.110971 | 4-hydroxy-2-oxovalerate aldolase |
| XCC3050 | 3.153287 | citrate-dependent iron transporter |
| XCC3051 | 4.244028 | carboxylate--amine ligase |
| XCC3052 | 4.293914 | IucA/IucC family siderophore biosynthesis protein |
| XCC3054 | 3.317131 | iron transporter |
| XCC3055 | 3.920353 | diaminopimelate decarboxylase |
| XCC3081 | 1.451379 | glucoamylase |
| XCC3082 | 1.637446 | trehalose-6-phosphate synthase |
| XCC3083 | 1.222481 | membrane-bound PQQ-dependent dehydrogenase, glucose/quinate/shikimate family |
| XCC3109 | 1.337172 | glycogen debranching enzyme |
| XCC3110 | -1.04745 | virulence regulator |
| XCC3111 | 1.69855 | hypothetical protein |
| XCC3113 | 11.5068 | ISxac3 transposase |
| XCC3125 | -1.82552 | transposase, partial |
| XCC3160 | 1.761869 | 1,4-beta-cellobiosidase |
| XCC3175 | -1.21553 | phosphoadenosine phosphosulfate reductase |
| XCC3176 | -1.13987 | peptidase |
| XCC3177 | -1.33902 | TonB-dependent receptor |
| XCC3184 | 2.415767 | O-acetyl-ADP-ribose deacetylase |
| XCC3210 | 1.104857 | peptidoglycan-binding protein |
| XCC3223 | 1.078976 | membrane protein |
| XCC3288 | 2.278792 | membrane protein |
| XCC3326 | -1.00497 | serine/threonine dehydratase |
| XCC3327 | -1.14652 | 2-isopropylmalate synthase |
| XCC3333 | 1.326241 | GGDEF domain-containing protein |
| XCC3350 | 1.123816 | ABC transporter substrate-binding protein |
| XCC3351 | 1.06301 | sensor histidine kinase |
| XCC3352 | 1.147512 | DNA-binding response regulator |
| XCC3360 | 1.043926 | dimethylallyltransferase |
| XCC3363 | 1.063374 | putative membrane protein |
| XCC3366 | 1.134747 | methyltransferase |
| XCC3370 | 1.381917 | hypothetical protein |
| XCC3377 | 2.660519 | truncated rhamnogalacturonase B |
| XCC3378 | 2.691457 | truncated rhamnogalacturonase B |
| XCC3379 | 2.61132 | truncated rhamnogalacturonase B |
| XCC3381 | 1.642099 | cellulase |
| XCC3383 | 2.13921 | hypothetical protein |
| XCC3402 | 3.229426 | beta-aspartyl-peptidase |
| XCC3403 | 3.509916 | betaine-aldehyde dehydrogenase |
| XCC3404 | 3.15371 | choline dehydrogenase |
| XCC3429 | -1.77466 | proline dioxygenase |
| XCC3436 | 1.041247 | sensor histidine kinase |
| XCC3437 | 3.205239 | BON domain-containing protein |
| XCC3441 | 1.662085 | membrane protein |
| XCC3455 | 1.417999 | membrane protein |
| XCC3462 | 1.047408 | type VI secretion protein |
| XCC3463 | -10.6573 | transposase |
| XCC3472 | 1.615206 | sigma-54-dependent Fis family transcriptional regulator |
| XCC3475 | -1.4132 | alcohol dehydrogenase |
| XCC3476 | -1.515 | surface antigen gene |
| XCC3487 | 2.421036 | MFS transporter |
| XCC3508 | 1.249353 | outer membrane lipoprotein |
| XCC3527 | 1.824315 | endonuclease |
| XCC3534 | 5.201132 | 1,4-beta-cellobiosidase |
| XCC3535 | 4.983488 | glycosidase |
| XCC3539 | 1.07045 | spermidine synthase-like enzyme |
| XCC3546 | 1.791892 | GGDEF domain-containing protein |
| XCC3560 | -2.01478 | glyoxalase |
| XCC3561 | 1.188077 | glutathione-dependent reductase |
| XCC3569 | -1.27498 | hypothetical protein |
| XCC3570 | -1.58496 | conserved hypothetical protein |
| XCC3589 | 1.055377 | hypothetical protein |
| XCC3590 | 1.24664 | hypothetical protein |
| XCC3593 | 1.619198 | RNA polymerase sigma factor |
| XCC3594 | 1.783717 | iron dicitrate transporter FecR |
| XCC3623 | 1.380098 | lipase |
| XCC3624 | 4.679601 | arabinogalactan endo-1,4-beta-galactosidase |
| XCC3640 | 2.21963 | hypothetical protein |
| XCC3642 | 2.16486 | hypothetical protein |
| XCC3643 | 1.836449 | hybrid sensor histidine kinase/response regulator |
| XCC3645 | 3.19849 | hypothetical protein |
| XCC3668 | 1.879375 | tryptophan repressor binding protein |
| XCC3670 | 3.123896 | glycosyl transferase family 2 |
| XCC3682 | -2.81574 | protein YciF |
| XCC3683 | -2.29624 | Mn-containing catalase |
| XCC3685 | -8.49016 | hypothetical protein |
| XCC3687 | -2.44649 | response regulator |
| XCC3689 | 1.280185 | sigma-54-dependent Fis family transcriptional regulator |
| XCC3724 | -1.30835 | aldo/keto reductase |
| XCC3725 | -1.30806 | LysR family transcriptional regulator |
| XCC3732 | -12.2848 | transposase |
| XCC3778 | -1.16865 | Transcription termination factor Rho |
| XCC3790 | -1.75996 | dehydrogenase |
| XCC3797 | 1.329403 | hypothetical protein |
| XCC3811 | 1.678165 | hypothetical protein |
| XCC3815 | 1.189515 | dihydroneopterin aldolase |
| XCC3819 | 1.790141 | ribonuclease BN |
| XCC3827 | 1.382368 | membrane protein |
| XCC3831 | 1.994273 | membrane protein |
| XCC3833 | 1.046208 | cytochrome c oxidase subunit II |
| XCC3865 | 1.169513 | acyltransferase |
| XCC3871 | 1.463277 | kinase |
| XCC3877 | 1.101893 | cyclic diguanylate phosphodiesterase |
| XCC3882 | -1.38262 | stomatin/prohibitin-family membrane protease subunit |
| XCC3883 | -1.23978 | RNA-splicing ligase RtcB |
| XCC3884 | -1.14688 | Conserved hypothetical protein |
| XCC3888 | 1.292247 | hypothetical protein |
| XCC3890 | 3.08092 | epimerase |
| XCC3894 | 1.628253 | two-component sensor histidine kinase |
| XCC3897 | 1.602564 | histidine biosynthesis protein HisIE |
| XCC3908 | -1.6446 | putative secreted protein |
| XCC3911 | -1.38904 | acriflavin resistance protein |
| XCC3912 | -1.31353 | ABC transporter ATP-binding protein |
| XCC3913 | -1.18318 | membrane protein |
| XCC3915 | -2.53329 | alpha/beta hydrolase |
| XCC3925 | 2.077226 | entericidin A |
| XCC3948 | 2.133963 | ankyrin-like protein |
| XCC3949 | 1.067381 | catalase |
| XCC3955 | 2.401824 | iron-uptake factor |
| XCC3963 | -1.09142 | TonB-dependent receptor |
| XCC3981 | 2.424079 | lipoprotein |
| XCC3989 | 3.077002 | hypothetical protein |
| XCC3991 | 2.62847 | carbonic anhydrase |
| XCC4028 | 1.060336 | cardiolipin synthase A |
| XCC4038 | 1.044747 | cation efflux system protein |
| XCC4040 | -1.041 | putative membrane protein |
| XCC4044 | 2.721772 | transglycosylase |
| XCC4048 | 1.203141 | phospholipase A(1) |
| XCC4050 | 1.086135 | ankyrin-like protein |
| XCC4057 | 3.01272 | cation acetate symporter |
| XCC4058 | 2.957499 | membrane protein |
| XCC4059 | 2.353342 | putative exported protein |
| XCC4060 | 1.131004 | acetate-CoA ligase |
| XCC4061 | 1.814428 | DNA-binding response regulator |
| XCC4063 | -2.15549 | cytochrome c biogenesis protein |
| XCC4080 | 1.175389 | polyvinylalcohol dehydrogenase |
| XCC4102 | 1.014179 | alpha-glucuronidase |
| XCC4103 | 1.117849 | 9-O-acetylesterase |
| XCC4108 | -1.69334 | restriction endonuclease or methylase |
| XCC4112 | -2.22698 | ring canal kelch-like protein |
| XCC4127 | 1.038351 | alcohol dehydrogenase |
| XCC4144 | 2.558304 | sensor histidine kinase |
| XCC4153 | 1.130554 | benzoate transporter |
| XCC4158 | 3.463488 | putative exported protein |
| XCC4162 | 2.828624 | TonB-dependent siderophore receptor |
| XCC4176 | 1.209506 | cardiolipin synthase B |
| XCC4195 | 1.081957 | lipoprotein |
| XCC4206 | 1.325335 | hypothetical protein |
| XCC4214 | 1.515798 | hypothetical protein |
| XCC4227 | -1.42105 | MFS transporter |
| XCC4232 | 1.476135 | membrane protein |
