# Supplementary material for: RpoN1 and RpoN2 play different regulatory roles in virulence traits, flagellar biosynthesis, and basal metabolism in Xanthomonas campestris
Source: Mol Plant Pathol. 2020 Apr 13;21(7):907–22. doi: 10.1111/mpp.12938 (PMC7280030; doi:10.1111/mpp.12938)
Supplement: Supplementary file 10 [file MPP-21-907-s010.docx]

**Table S4. List of genes differentially expressed in the Δ*rpoN2* mutant compared to the wild-type strain (log_2_ fold change ≥ 1).**

| Gene name | log2 fold change (Δ*rpoN2* / Xc1) | Gene Description |
| --- | --- | --- |
| XCC0017 | -1.19195 | membrane protein |
| XCC0025 | 2.138704 | peptidase propeptide and ypeb domain-containing protein |
| XCC0027 | 1.532772 | cellulase |
| XCC0028 | 1.158061 | cellulase |
| XCC0034 | 2.643193 | DNA topoisomerase |
| XCC0036 | 2.026003 | hypothetical protein |
| XCC0070 | 2.016322 | CsbD family protein |
| XCC0077 | 1.896396 | NAD-dependent dehydratase |
| XCC0102 | 1.442732 | acetaldehyde dehydrogenase |
| XCC0104 | 3.212246 | Ku protein |
| XCC0113 | 1.949244 | hypothetical protein |
| XCC0133 | 3.261454 | alpha-amylase |
| XCC0134 | 3.586339 | trehalose synthase |
| XCC0141 | 3.852219 | L-fucose dehydrogenase |
| XCC0158 | -1.28138 | TonB-dependent siderophore receptor |
| XCC0167 | 2.523945 | ergothioneine biosynthesis protein EgtB |
| XCC0168 | 3.071478 | L-histidine N(alpha)-methyltransferase |
| XCC0191 | 1.207815 | superoxide dismutase |
| XCC0206 | 2.32317 | pyruvate oxidase |
| XCC0230 | -1.26776 | dipeptidyl carboxypeptidase II |
| XCC0241 | -4.45983 | putative modified peptide |
| XCC0247 | 1.048032 | AcrR family transcriptional regulator |
| XCC0250 | -4.39731 | membrane protein |
| XCC0253 | -1.45725 | nickel uptake substrate-specific transmembrane region family protein |
| XCC0268 | 1.999479 | hypothetical protein |
| XCC0274 | 1.982562 | conserved small membrane protein |
| XCC0276 | -4.20824 | methyl-accepting chemotaxis protein |
| XCC0289 | 11.51668 | aminoacyl-tRNA hydrolase |
| XCC0302 | 2.115381 | MFS transporter |
| XCC0313 | 2.39897 | conditioned medium factor |
| XCC0320 | 1.415037 | porin |
| XCC0350 | -4.07276 | phosphodiesterase |
| XCC0358 | 1.924609 | glycerol kinase |
| XCC0359 | 1.553481 | aquaporin |
| XCC0360 | 1.531102 | glycerol-3-phosphate dehydrogenase |
| XCC0393 | -12.9144 | transposase, partial |
| XCC0395 | 1.251221 | superoxide dismutase |
| XCC0398 | 1.140241 | isopenicillin-N epimerase |
| XCC0401 | 2.582668 | hypothetical protein |
| XCC0404 | 2.706287 | ABC transporter substrate-binding protein |
| XCC0407 | -3.33873 | GGDEF domain-containing protein |
| XCC0408 | 2.430467 | starch synthase |
| XCC0409 | 2.160581 | 1,4-alpha-glucan branching enzyme |
| XCC0431 | 1.736771 | nuclease |
| XCC0458 | 1.541166 | diacylglycerol kinase-like enzyme |
| XCC0476 | -1.23119 | 50S ribosomal protein L13 |
| XCC0477 | -1.02146 | 30S ribosomal protein S9 |
| XCC0482 | 1.914667 | bacterioferritin |
| XCC0487 | 1.006639 | cell shape determination protein CcmA |
| XCC0535 | 2.855816 | IS5/IS1182 family transposase |
| XCC0538 | 1.353047 | secreted protein |
| XCC0546 | 3.50832 | secreted protein |
| XCC0570 | 2.855816 | IS5/IS1182 family transposase |
| XCC0572 | -1.88172 | Putative secreted protein |
| XCC0596 | 1.203559 | UptF |
| XCC0638 | 2.855816 | IS5/IS1182 family transposase |
| XCC0659 | 2.311173 | protease |
| XCC0694 | -2.88956 | putative exported protein |
| XCC0741 | 1.285833 | hypothetical protein |
| XCC0748 | 2.311481 | alpha-amylase |
| XCC0779 | 1.49128 | DNA-binding response regulator |
| XCC0811 | -1.64481 | MarR family transcriptional regulator |
| XCC0812 | -2.123 | hypothetical protein |
| XCC0815 | 1.139708 | glucose-fructose oxidoreductase |
| XCC0827 | 7.632995 | DUF4440 domain-containing protein |
| XCC0829 | 1.110374 | glutamine amidotransferase |
| XCC0847 | 1.699906 | RNA polymerase sigma factor |
| XCC0851 | 1.679664 | protease |
| XCC0852 | 1.997737 | peptidase S8 |
| XCC0854 | -3.05024 | peptidase S8 |
| XCC0863 | -3.98004 | putative membrane protein |
| XCC0874 | -1.29908 | 50S ribosomal protein L25/general stress protein Ctc |
| XCC0884 | -1.1745 | 50S ribosomal protein L11 |
| XCC0885 | -1.22961 | 50S ribosomal protein L1 |
| XCC0886 | -1.71278 | 50S ribosomal protein L10 |
| XCC0890 | -1.15516 | 30S ribosomal protein S12 |
| XCC0891 | -1.18186 | 30S ribosomal protein S7 |
| XCC0895 | -1.1241 | 50S ribosomal protein L3 |
| XCC0896 | -1.28176 | 50S ribosomal protein L4 |
| XCC0897 | -1.486 | 50S ribosomal protein L23 |
| XCC0898 | -1.42917 | 50S ribosomal protein L2 |
| XCC0899 | -1.38852 | 30S ribosomal protein S19 |
| XCC0900 | -1.58616 | 50S ribosomal protein L22 |
| XCC0901 | -1.5017 | 30S ribosomal protein S3 |
| XCC0902 | -1.49145 | 50S ribosomal protein L16 |
| XCC0904 | -1.27927 | 30S ribosomal protein S17 |
| XCC0905 | -1.569 | 50S ribosomal protein L14 |
| XCC0906 | -1.51553 | 50S ribosomal protein L24 |
| XCC0907 | -1.50974 | 50S ribosomal protein L5 |
| XCC0908 | -1.55811 | 30S ribosomal protein S14 |
| XCC0909 | -1.03339 | 30S ribosomal protein S8 |
| XCC0910 | -1.05518 | 50S ribosomal protein L6 |
| XCC0911 | -1.2784 | 50S ribosomal protein L18 |
| XCC0912 | -1.17587 | 30S ribosomal protein S5 |
| XCC0913 | -1.56412 | 50S ribosomal protein L30 |
| XCC0915 | -1.11868 | preprotein translocase subunit SecY |
| XCC0916 | -1.16252 | 30S ribosomal protein S13 |
| XCC0917 | -1.17991 | 30S ribosomal protein S11 |
| XCC0918 | -1.09623 | 30S ribosomal protein S4 |
| XCC0919 | -1.16452 | DNA-directed RNA polymerase subunit alpha |
| XCC0920 | -1.47736 | 50S ribosomal protein L17 |
| XCC0923 | 1.599107 | membrane protein |
| XCC0955 | 1.589787 | peptidyl-Asp metalloendopeptidase |
| XCC1045 | 2.908684 | bacterioferritin |
| XCC1046 | 2.28423 | peroxiredoxin |
| XCC1054 | -1.01857 | aminopeptidase |
| XCC1072 | 1.013261 | type III secretion system effector protein |
| XCC1079 | 3.329278 | NAD(P)-dependent oxidoreductase |
| XCC1084 | 1.201517 | thioredoxin reductase |
| XCC1086 | -1.98893 | EAL domain-containing protein |
| XCC1089 | 1.027417 | transducer protein car |
| XCC1091 | 2.708564 | aldehyde oxidase |
| XCC1092 | 2.372025 | FAD-binding molybdopterin dehydrogenase |
| XCC1093 | 2.124438 | ferredoxin |
| XCC1097 | 4.107819 | DNA-binding protein |
| XCC1118 | 2.097079 | membrane protein |
| XCC1142 | 1.709214 | calcium-binding protein |
| XCC1149 | -1.04427 | 50S ribosomal protein L21 |
| XCC1165 | -1.65093 | DUF805 domain-containing protein |
| XCC1181 | 1.609823 | RNA signal recognition particle 4.5S RNA |
| XCC1191 | 1.10382 | alpha-L-arabinofuranosidase |
| XCC1209 | 2.855816 | IS5/IS1182 family transposase |
| XCC1219 | 1.380804 | HrpW protein |
| XCC1221 | 1.724818 | HPr kinase |
| XCC1236 | 1.278223 | EscN/YscN/HrcN family type III secretion system ATPase |
| XCC1239 | 1.311517 | EscC/YscC/HrcC family type III secretion system outer membrane ring protein |
| XCC1240 | 1.555245 | Hpa1 protein |
| XCC1255 | 2.855816 | IS5/IS1182 family transposase |
| XCC1289 | 1.882037 | DNA ligase-associated DEXH box helicase |
| XCC1308 | 1.093993 | putative exported protein |
| XCC1311 | 3.196955 | cation transporter |
| XCC1318 | 2.252479 | hypothetical protein |
| XCC1350 | -2.27393 | hypothetical protein |
| XCC1374 | -1.45174 | elongation factor Ts |
| XCC1375 | -1.20079 | 30S ribosomal protein S2 |
| XCC1376 | -2.46827 | pilus assembly protein |
| XCC1377 | -3.2207 | putative exported protein |
| XCC1379 | 1.072247 | outer membrane usher protein FasD |
| XCC1381 | 1.087734 | protein U |
| XCC1388 | -1.29152 | asparagine synthase B |
| XCC1391 | -1.63372 | TonB-dependent siderophore receptor |
| XCC1393 | 1.455333 | bacterioferritin |
| XCC1401 | -1.51298 | Putative secreted protein |
| XCC1404 | -1.67722 | glycosyl hydrolase |
| XCC1408 | -3.48395 | hypothetical protein |
| XCC1412 | -1.88419 | dipeptidyl carboxypeptidase II |
| XCC1433 | 2.382233 | NADPH-dependent oxidoreductase |
| XCC1438 | 1.84345 | aklaviketone reductase |
| XCC1439 | 1.868957 | MexE family multidrug efflux RND transporter periplasmic adaptor subunit |
| XCC1440 | 1.868028 | multidrug efflux RND transporter permease subunit |
| XCC1441 | 1.871883 | short-chain dehydrogenase |
| XCC1442 | 1.767263 | RND transporter |
| XCC1443 | -2.14989 | GGDEF domain-containing protein |
| XCC1444 | 2.78445 | hypothetical protein |
| XCC1445 | -4.2379 | sensor histidine kinase |
| XCC1454 | 2.855816 | IS5/IS1182 family transposase |
| XCC1494 | 1.79864 | class II fumarate hydratase |
| XCC1524 | 1.322331 | phosphate ABC transporter ATP-binding protein |
| XCC1539 | 1.258495 | N-acetylmuramoyl-L-alanine amidase |
| XCC1565 | -1.1771 | 50S ribosomal protein L9 |
| XCC1575 | 3.467153 | membrane-bound PQQ-dependent dehydrogenase, glucose/quinate/shikimate family |
| XCC1576 | -2.6802 | secreted protein |
| XCC1577 | 1.350194 | urocanate hydratase |
| XCC1634 | 11.36085 | ISxac3 transposase |
| XCC1636 | 4.214795 | IS5/IS1182 family transposase |
| XCC1638 | 2.855816 | IS5/IS1182 family transposase |
| XCC1647 | 3.058154 | hypothetical protein |
| XCC1653 | 2.631088 | response regulator |
| XCC1685 | 1.19034 | MFS transporter |
| XCC1727 | -3.51894 | chemotaxis protein |
| XCC1750 | -1.51023 | TonB-dependent receptor, partial |
| XCC1751 | -1.53196 | TonB-dependent receptor, partial |
| XCC1777 | -4.1312 | sensor domain-containing phosphodiesterase |
| XCC1778 | 1.064722 | mannan endo-1,4-beta-mannosidase |
| XCC1797 | 2.855816 | IS5/IS1182 family transposase |
| XCC1865 | -4.00796 | bifunctional diguanylate cyclase/phosphodiesterase |
| XCC1866 | -4.401 | chemotaxis response regulator protein-glutamate methylesterase |
| XCC1867 | -4.03869 | chemoreceptor glutamine deamidase CheD |
| XCC1868 | -5.0882 | chemotaxis protein CheR |
| XCC1869 | -5.39316 | methyl-accepting chemotaxis protein |
| XCC1870 | -5.08235 | putative secreted protein |
| XCC1871 | -5.09797 | chemotaxis protein |
| XCC1872 | -4.57515 | pilus assembly protein PilZ |
| XCC1873 | -4.30812 | chemotaxis protein |
| XCC1874 | -4.43965 | methyl-accepting chemotaxis protein |
| XCC1876 | -4.17193 | chemotaxis protein |
| XCC1877 | -13.6073 | methyl-accepting chemotaxis protein |
| XCC1878 | -4.77966 | methyl-accepting chemotaxis protein |
| XCC1879 | -5.1061 | methyl-accepting chemotaxis protein |
| XCC1880 | -4.15701 | chemotaxis protein |
| XCC1881 | -4.43225 | chemotaxis protein |
| XCC1882 | -5.32982 | chemotaxis protein, partial |
| XCC1883 | -6.37244 | lipoprotein |
| XCC1884 | -2.3901 | methyl-accepting chemotaxis protein |
| XCC1885 | -2.41917 | chemotaxis protein CheA |
| XCC1886 | -2.69657 | chemotaxis response regulator |
| XCC1887 | -2.47669 | anti-sigma factor antagonist-like protein |
| XCC1890 | -2.91744 | flagellar motor protein MotD |
| XCC1891 | -4.58296 | flagellar motor protein |
| XCC1903 | -1.52972 | chemotaxis protein CheA |
| XCC1909 | -1.795 | flagellar biosynthesis protein FlhA |
| XCC1910 | -2.99653 | flagellar biosynthesis protein FlhB |
| XCC1911 | -4.09523 | bifunctional diguanylate cyclase/phosphodiesterase |
| XCC1912 | -2.35364 | GGDEF domain-containing protein |
| XCC1916 | 2.812702 | flagellar biosynthetic protein FliP |
| XCC1917 | 2.761241 | flagellar protein |
| XCC1918 | 2.913999 | flagellar motor switch protein FliN |
| XCC1919 | 3.09437 | flagellar motor switch protein FliM |
| XCC1920 | 2.872654 | flagellar biosynthesis protein |
| XCC1927 | -1.83383 | flagellar hook-basal body complex protein FliE |
| XCC1928 | 1.236567 | O-antigen biosynthesis protein |
| XCC1929 | 1.235206 | Methyltransferase domain-containing protein |
| XCC1930 | 1.052794 | methyltransferase domain protein |
| XCC1931 | 1.324035 | carboxyl transferase |
| XCC1932 | 1.475478 | aminotransferase |
| XCC1933 | 1.250632 | sigma-54-dependent Fis family transcriptional regulator |
| XCC1934 | 1.203311 | response regulator |
| XCC1937 | -2.00119 | PilZ domain-containing protein |
| XCC1938 | -1.94278 | hypothetical protein |
| XCC1939 | -2.7817 | flagellar protein FliS |
| XCC1940 | -1.7933 | flagellar protein |
| XCC1941 | -3.32338 | flagellin |
| XCC1948 | 1.718527 | flagellar basal body rod protein FlgF |
| XCC1949 | 1.853817 | flagellar hook protein FlgE |
| XCC1950 | 1.82024 | flagellar basal body rod modification protein FlgD |
| XCC1951 | 1.751505 | flagellar basal body rod protein FlgC |
| XCC1953 | -4.07029 | chemotaxis protein |
| XCC1954 | -2.59006 | flagellar basal body P-ring biosynthesis protein FlgA |
| XCC1955 | -3.2513 | flagellar biosynthesis anti-sigma factor FlgM |
| XCC1956 | -3.24541 | flagella protein |
| XCC1959 | -3.62958 | GGDEF domain-containing protein |
| XCC1961 | -3.33097 | hypothetical protein |
| XCC1962 | -4.36342 | chemotaxis protein |
| XCC1990 | 2.553875 | TonB-dependent receptor |
| XCC1992 | 4.763412 | short-chain dehydrogenase |
| XCC1993 | 5.571542 | cytochrome P450 |
| XCC1994 | 4.628524 | hypothetical protein |
| XCC1996 | 4.043236 | glutathione-dependent formaldehyde dehydrogenase |
| XCC1998 | 2.887412 | exodeoxyribonuclease III |
| XCC1999 | 2.792195 | membrane protein |
| XCC2002 | 1.369366 | hypothetical protein |
| XCC2012 | 2.21661 | NAD(P)-dependent oxidoreductase |
| XCC2016 | 2.898503 | hypothetical protein |
| XCC2017 | 1.166449 | dehydrogenase |
| XCC2018 | 2.678072 | hypothetical protein |
| XCC2020 | 4.992466 | YciE |
| XCC2024 | 1.135016 | YapH protein |
| XCC2025 | -2.32193 | serine protease |
| XCC2031 | 2.927036 | membrane protein |
| XCC2032 | 3.015891 | sorbosone dehydrogenase |
| XCC2035 | 1.085343 | peptidase |
| XCC2036 | 2.051811 | hydroxylase |
| XCC2038 | 1.780541 | SCO family protein |
| XCC2039 | 2.224193 | transporter |
| XCC2041 | 2.355826 | transcriptional regulator |
| XCC2047 | -3.03905 | methyl-accepting chemotaxis protein |
| XCC2105 | 2.855816 | IS5/IS1182 family transposase |
| XCC2124 | 1.290906 | NAD dependent epimerase/dehydratase |
| XCC2181 | -2.43118 | HAMP domain-containing protein |
| XCC2182 | -1.67276 | hypothetical protein |
| XCC2208 | -1.32339 | membrane protein |
| XCC2230 | 1.894847 | thiol reductant ABC exporter subunit CydC |
| XCC2231 | 1.305708 | thiol reductant ABC exporter subunit CydD |
| XCC2232 | 1.598608 | cytochrome d ubiquinol oxidase subunit I |
| XCC2233 | 2.522257 | cytochrome D ubiquinol oxidase subunit II |
| XCC2234 | 3.13913 | cyd operon protein YbgT |
| XCC2254 | 2.698126 | hypothetical protein |
| XCC2262 | 2.750698 | hypothetical protein |
| XCC2264 | 3.283905 | general stress protein |
| XCC2284 | 1.209581 | SELO family protein |
| XCC2299 | 1.126605 | N-acetylmuramoyl-L-alanine amidase |
| XCC2307 | 1.760221 | ATP-dependent DNA ligase |
| XCC2308 | 3.973702 | DUF3606 domain-containing protein |
| XCC2312 | 3.471967 | RecA/RadA recombinase |
| XCC2314 | -3.47182 | chemotaxis protein CheW |
| XCC2315 | -4.39324 | methyl-accepting chemotaxis protein |
| XCC2322 | 1.142507 | ubiquinol cytochrome C oxidoreductase |
| XCC2336 | 1.743726 | succinate-semialdehyde dehydrogenase |
| XCC2350 | -4.68274 | diguanylate cyclase |
| XCC2354 | -1.80226 | formate dehydrogenase a chain |
| XCC2355 | -2.00577 | sulfurtransferase FdhD |
| XCC2356 | -3.04002 | MFS transporter |
| XCC2360 | 1.174886 | histidine kinase |
| XCC2361 | 1.312824 | two-component system response regulator |
| XCC2402 | -10.0498 | hypothetical protein |
| XCC2403 | -3.5248 | hypothetical protein |
| XCC2425 | 5.202848 | stress-induced protein |
| XCC2443 | 1.421852 | GumM protein |
| XCC2444 | 1.527944 | GumL protein |
| XCC2445 | 1.646469 | UDP-glucuronate--glycolipid 2-beta-glucuronosyltransferase |
| XCC2446 | 1.94485 | lipopolysaccharide biosynthesis protein |
| XCC2447 | 1.941236 | GDP-mannose--glycolipid 4-beta-D-mannosyltransferase |
| XCC2448 | 1.882732 | glycosyl transferase family 1 |
| XCC2449 | 1.992749 | GumG protein |
| XCC2450 | 1.723495 | GumF protein |
| XCC2451 | 2.542248 | gumE protein |
| XCC2452 | 2.966436 | GumD protein |
| XCC2453 | 1.758048 | GumC protein |
| XCC2454 | 1.725844 | polysaccharide biosynthesis protein GumB |
| XCC2458 | -1.2438 | phenylalanine--tRNA ligase subunit beta |
| XCC2490 | 2.855816 | IS5/IS1182 family transposase |
| XCC2541 | -1.45475 | tryptophan synthase subunit alpha |
| XCC2542 | -1.08495 | hypothetical protein |
| XCC2559 | 1.27289 | GDP-mannose pyrophosphatase |
| XCC2574 | -1.57237 | peptidase |
| XCC2624 | 2.828999 | conserved membrane protein |
| XCC2630 | 2.424244 | hypothetical protein |
| XCC2632 | -1.03508 | putative secreted protein |
| XCC2641 | -3.59873 | GGDEF domain-containing protein |
| XCC2658 | -1.25138 | ligand-gated channel |
| XCC2663 | 3.844129 | Uncharacterized conserved protein YjgD, DUF1641 family |
| XCC2664 | 5.008607 | formate dehydrogenase subunit alpha |
| XCC2666 | 2.158636 | serine protease |
| XCC2667 | 1.735069 | serine protease |
| XCC2692 | 1.693825 | cell envelope integrity protein CreD |
| XCC2699 | -5.35491 | anti-anti-sigma factor |
| XCC2700 | -4.59715 | chemotaxis protein CheA |
| XCC2701 | -4.86303 | methyl-accepting chemotaxis protein |
| XCC2702 | -3.49261 | chemotaxis protein CheW |
| XCC2703 | -3.81295 | transcriptional regulator |
| XCC2704 | -4.13564 | SAM-dependent methyltransferase |
| XCC2705 | -4.72103 | chemotaxis response regulator protein-glutamate methylesterase |
| XCC2728 | 1.244057 | oxidoreductase |
| XCC2729 | 1.3219 | aldehyde dehydrogenase iron-sulfur subunit |
| XCC2745 | 2.959906 | peroxiredoxin |
| XCC2772 | -1.83608 | TonB-dependent siderophore receptor |
| XCC2773 | -2.20169 | PKHD-type hydroxylase |
| XCC2774 | -2.30857 | Sel1 repeat-containing protein |
| XCC2776 | -2.44194 | conserved exported protein |
| XCC2777 | -2.51045 | ABC transporter permease |
| XCC2803 | 1.062123 | sigma-54 modulation protein |
| XCC2812 | 2.326784 | cytochrome D ubiquinol oxidase subunit II |
| XCC2813 | 1.865497 | cytochrome ubiquinol oxidase subunit I |
| XCC2820 | 2.559328 | endoproteinase ArgC, partial |
| XCC2821 | 2.292372 | serine protease |
| XCC2822 | 1.54223 | protein-glutamate methylesterase |
| XCC2823 | 4.408247 | stress-induced protein |
| XCC2845 | 2.03661 | MFS transporter |
| XCC2861 | 2.843902 | membrane protein |
| XCC2867 | -1.27476 | TonB-dependent receptor |
| XCC2887 | -1.66531 | TonB-dependent receptor |
| XCC2899 | 1.008462 | type III secretion system effector protein |
| XCC2907 | -3.69335 | MULTISPECIES: hypothetical protein |
| XCC2908 | -4.29422 | RebB protein |
| XCC2921 | -1.06915 | two-component system sensor histidine kinase/response regulator |
| XCC2922 | -1.06698 | pilus biogenesis protein |
| XCC2923 | -1.42675 | pilus biogenesis protein |
| XCC2924 | -1.57242 | response regulator |
| XCC2925 | -1.21947 | response regulator |
| XCC2949 | 1.214551 | response regulator |
| XCC2958 | 1.985553 | response regulator |
| XCC3034 | 1.424315 | MFS transporter |
| XCC3045 | 1.054313 | ligand-gated channel |
| XCC3046 | 1.476212 | ferric enterobactin receptor |
| XCC3074 | -1.20457 | membrane protein |
| XCC3081 | 1.893349 | glucoamylase |
| XCC3082 | 2.510604 | trehalose-6-phosphate synthase |
| XCC3084 | -3.63024 | chemotaxis protein |
| XCC3109 | 2.189974 | glycogen debranching enzyme |
| XCC3113 | 11.36085 | ISxac3 transposase |
| XCC3122 | 2.855816 | IS5/IS1182 family transposase |
| XCC3160 | 1.966127 | 1,4-beta-cellobiosidase |
| XCC3161 | 1.184386 | TonB-dependent receptor |
| XCC3162 | 1.70008 | glycoside hydrolase family 2 |
| XCC3168 | -1.33999 | aminopeptidase |
| XCC3176 | 1.83719 | peptidase |
| XCC3177 | 2.068128 | TonB-dependent receptor |
| XCC3184 | 2.686557 | O-acetyl-ADP-ribose deacetylase |
| XCC3193 | -11.6861 | conserved exported protein |
| XCC3194 | 1.504929 | membrane protein |
| XCC3199 | 11.4818 | IS5/IS1182 family transposase |
| XCC3210 | 2.110547 | peptidoglycan-binding protein |
| XCC3214 | 2.855816 | IS5/IS1182 family transposase |
| XCC3312 | 1.463373 | K+-insensitive pyrophosphate-energized proton pump |
| XCC3317 | 2.31202 | transcriptional regulator |
| XCC3321 | -1.03201 | chemotaxis protein |
| XCC3343 | 2.855816 | IS5/IS1182 family transposase |
| XCC3346 | -1.48268 | C4-dicarboxylate transporter |
| XCC3347 | -1.48942 | porin |
| XCC3382 | -1.87667 | fatty acid-binding protein DegV |
| XCC3392 | 2.992421 | C4-dicarboxylate ABC transporter |
| XCC3435 | 1.199063 | response regulator |
| XCC3437 | 4.234078 | BON domain-containing protein |
| XCC3441 | 2.793717 | membrane protein |
| XCC3463 | -10.6573 | transposase |
| XCC3474 | 2.794977 | TonB-dependent receptor |
| XCC3475 | 2.609323 | alcohol dehydrogenase |
| XCC3476 | 2.313756 | surface antigen gene |
| XCC3477 | 2.888233 | hypothetical protein |
| XCC3478 | 2.817666 | MoxJ protein |
| XCC3479 | 2.764129 | methanol dehydrogenase |
| XCC3482 | 3.350778 | methanol dehydrogenase |
| XCC3519 | -2.50112 | GGDEF domain-containing protein |
| XCC3522 | -4.37683 | chemotaxis protein |
| XCC3523 | -3.9263 | sensor domain-containing diguanylate cyclase |
| XCC3527 | 2.595948 | endonuclease |
| XCC3543 | -2.40854 | lipoprotein |
| XCC3546 | -10.388 | GGDEF domain-containing protein |
| XCC3561 | 1.795737 | glutathione-dependent reductase |
| XCC3578 | -12.5746 | transposase |
| XCC3586 | 2.855816 | IS5/IS1182 family transposase |
| XCC3591 | 2.491422 | gluconolactonase |
| XCC3594 | -2.14439 | iron dicitrate transporter FecR |
| XCC3595 | -1.67892 | TonB-dependent siderophore receptor |
| XCC3627 | 2.855816 | IS5/IS1182 family transposase |
| XCC3640 | 3.988549 | hypothetical protein |
| XCC3642 | 2.885679 | hypothetical protein |
| XCC3643 | 2.242692 | hybrid sensor histidine kinase/response regulator |
| XCC3645 | 3.824493 | hypothetical protein |
| XCC3653 | -3.27083 | flagellar motor protein MotA |
| XCC3654 | -3.38986 | flagellar motor protein MotB |
| XCC3670 | 3.827377 | glycosyl transferase family 2 |
| XCC3672 | 1.369728 | hypothetical protein |
| XCC3681 | 12.19229 | YciE |
| XCC3682 | 3.747278 | protein YciF |
| XCC3683 | 2.467108 | Mn-containing catalase |
| XCC3685 | 1.228164 | hypothetical protein |
| XCC3687 | 2.529072 | response regulator |
| XCC3690 | 5.529628 | hypothetical protein |
| XCC3691 | 2.376223 | cyanide insensitive terminal oxidase |
| XCC3692 | 2.734974 | cytochrome D ubiquinol oxidase subunit II |
| XCC3694 | 1.811198 | membrane protein |
| XCC3695 | 2.177054 | oxidoreductase |
| XCC3702 | 3.66862 | hypothetical protein |
| XCC3708 | 12.68497 | DNA-binding protein |
| XCC3735 | 3.29512 | hypothetical protein |
| XCC3783 | 1.979703 | putative exported protein |
| XCC3784 | 2.814644 | peptidoglycan-binding protein LysM |
| XCC3790 | 1.362723 | dehydrogenase |
| XCC3797 | 1.876779 | hypothetical protein |
| XCC3798 | 1.235763 | EF hand domain-containing protein |
| XCC3810 | 2.545016 | hypothetical protein |
| XCC3811 | 2.985786 | hypothetical protein |
| XCC3813 | 1.085085 | dehydrogenase |
| XCC3819 | 1.842805 | ribonuclease BN |
| XCC3829 | 1.172875 | cytochrome c oxidase subunit III |
| XCC3830 | 1.063188 | cytochrome c oxidase assembly protein |
| XCC3832 | 1.156179 | cytochrome c oxidase subunit I |
| XCC3848 | -1.34144 | putative exported protein |
| XCC3870 | -1.79339 | hypothetical protein |
| XCC3886 | 1.826436 | hypothetical protein |
| XCC3887 | 1.956668 | hypothetical protein |
| XCC3888 | 3.47326 | hypothetical protein |
| XCC3889 | 2.682072 | lipoprotein, putative |
| XCC3891 | 1.237717 | mercuric reductase |
| XCC3899 | 1.485326 | hypothetical protein |
| XCC3901 | 1.209381 | hypothetical protein |
| XCC3925 | 2.639128 | entericidin A |
| XCC3947 | -3.4251 | membrane protein |
| XCC3967 | -1.14805 | cell envelope biogenesis protein TonB |
| XCC4024 | 1.744449 | universal stress protein A |
| XCC4042 | 3.332708 | alkaline phosphatase |
| XCC4063 | 1.339607 | cytochrome c biogenesis protein |
| XCC4111 | 2.0164 | histone |
| XCC4132 | -1.22477 | membrane protein |
| XCC4133 | -1.12862 | tryptophan halogenase |
| XCC4158 | 4.108709 | putative exported protein |
| XCC4185 | 2.747726 | IS1479 transposase |
| XCC4192 | 2.855816 | IS5/IS1182 family transposase |
| XCC4195 | 1.417356 | lipoprotein |
| XCC4214 | 2.273413 | hypothetical protein |
| XCC4221 | 1.367368 | alpha-1 2-mannosidase |
