# Supplementary material for: RpoN1 and RpoN2 play different regulatory roles in virulence traits, flagellar biosynthesis, and basal metabolism in Xanthomonas campestris
Source: Mol Plant Pathol. 2020 Apr 13;21(7):907–22. doi: 10.1111/mpp.12938 (PMC7280030; doi:10.1111/mpp.12938)
Supplement: Supplementary file 11 [file MPP-21-907-s011.docx]

**Table S5. List of genes differentially expressed in the Δ*rpoN1N2* mutant compared to the wild-type strain (log_2_ fold change ≥ 1).**

| Gene name | log2 fold change (Δ*rpoN1N2* / Xc1) | Gene Description |
| --- | --- | --- |
| XCC0017 | -1.75787 | membrane protein |
| XCC0025 | 3.106605 | peptidase propeptide and ypeb domain-containing protein |
| XCC0026 | -7.568 | cellulase |
| XCC0027 | 1.707429 | cellulase |
| XCC0028 | 1.360617 | cellulase |
| XCC0029 | 2.929031 | alcohol dehydrogenase |
| XCC0034 | 3.270873 | DNA topoisomerase |
| XCC0035 | 2.194401 | SAM-dependent methyltransferase |
| XCC0036 | 4.047302 | hypothetical protein |
| XCC0052 | 1.614041 | avirulence protein |
| XCC0056 | 1.666897 | hemolysin III |
| XCC0070 | 2.567468 | CsbD family protein |
| XCC0072 | -2.40872 | hypothetical protein |
| XCC0076 | 2.294368 | metalloprotease |
| XCC0077 | 2.18687 | NAD-dependent dehydratase |
| XCC0083 | 1.584963 | proline/betaine transporter |
| XCC0084 | -2.20031 | ATPase |
| XCC0101 | 1.309556 | aldehyde dehydrogenase family protein |
| XCC0102 | 1.624259 | acetaldehyde dehydrogenase |
| XCC0103 | 2.437347 | hypothetical protein xcc-b100_0113 |
| XCC0104 | 3.134407 | Ku protein |
| XCC0105 | 2.631727 | ATP-dependent DNA ligase |
| XCC0119 | -2.33939 | TonB-dependent receptor |
| XCC0120 | -2.75851 | TonB-dependent receptor |
| XCC0121 | -2.29108 | pectin methylesterase |
| XCC0122 | -2.12122 | pectate lyase |
| XCC0133 | 3.118784 | alpha-amylase |
| XCC0134 | 3.59379 | trehalose synthase |
| XCC0135 | 1.154391 | 1,4-alpha-glucan branching enzyme |
| XCC0141 | 4.309966 | L-fucose dehydrogenase |
| XCC0145 | -1.11814 | 2-keto-4-pentenoate hydratase |
| XCC0146 | 3.149574 | C4-dicarboxylate ABC transporter |
| XCC0147 | 3.180773 | C4-dicarboxylate ABC transporter |
| XCC0148 | 2.122433 | C4-dicarboxylate transport protein |
| XCC0149 | 1.851512 | xylosidase/arabinosidase |
| XCC0154 | -1.31884 | rhamnogalacturonan acetylesterase |
| XCC0165 | 1.470543 | amino acid ABC transporter permease |
| XCC0167 | 2.463145 | ergothioneine biosynthesis protein EgtB |
| XCC0168 | 2.790414 | L-histidine N(alpha)-methyltransferase |
| XCC0171 | -2.15664 | putative exported protein |
| XCC0182 | 1.246342 | phosphatidylethanolamine-binding protein |
| XCC0191 | 2.111117 | superoxide dismutase |
| XCC0205 | 1.072003 | Ax21 family protein |
| XCC0206 | 3.16839 | pyruvate oxidase |
| XCC0232 | 1.546779 | hydrolase |
| XCC0237 | 3.836262 | malate synthase A |
| XCC0238 | 4.546101 | isocitrate lyase |
| XCC0241 | -2.22172 | putative modified peptide |
| XCC0254 | -1.11212 | cytochrome c |
| XCC0269 | 1.710259 | 2,5-diketo-D-gluconate reductase B |
| XCC0270 | 2.112985 | NADP-dependent oxidoreductase |
| XCC0271 | 1.253911 | oxidoreductase |
| XCC0272 | 1.170795 | methyltransferase |
| XCC0274 | 2.659511 | conserved small membrane protein |
| XCC0276 | -6.05836 | methyl-accepting chemotaxis protein |
| XCC0289 | 10.02237 | aminoacyl-tRNA hydrolase |
| XCC0313 | 2.473443 | conditioned medium factor |
| XCC0316 | -1.03589 | FMN reductase |
| XCC0322 | 1.003468 | HxlR family transcriptional regulator |
| XCC0350 | -5.64534 | phosphodiesterase |
| XCC0353 | 1.612443 | 3-alpha-hydroxysteroid dehydrogenase |
| XCC0354 | 1.776456 | benzaldehyde dehydrogenase |
| XCC0356 | -9.39874 | 4-hydroxybenzoate 3-monooxygenase |
| XCC0357 | 4.364491 | hypothetical protein |
| XCC0358 | 2.27817 | glycerol kinase |
| XCC0359 | 1.94518 | aquaporin |
| XCC0360 | 2.161338 | glycerol-3-phosphate dehydrogenase |
| XCC0362 | 1.116569 | diguanylate cyclase |
| XCC0363 | 1.484206 | (2Fe-2S)-binding protein |
| XCC0384 | 1.339643 | oxidoreductase |
| XCC0388 | 1.820294 | biotin synthase |
| XCC0389 | 3.638138 | amidophosphoribosyltransferase |
| XCC0394 | -1.69839 | TonB-dependent siderophore receptor |
| XCC0397 | -1.50655 | TonB-dependent receptor |
| XCC0401 | 3.139824 | hypothetical protein |
| XCC0403 | 1.27164 | phosphoglycerol transferase I |
| XCC0404 | 3.710742 | ABC transporter substrate-binding protein |
| XCC0407 | -5.85522 | GGDEF domain-containing protein |
| XCC0408 | 1.483231 | starch synthase |
| XCC0410 | 1.205743 | malto-oligosyltrehalose trehalohydrolase |
| XCC0411 | 1.589429 | 4-alpha-glucanotransferase |
| XCC0414 | 1.235628 | glycogen debranching enzyme |
| XCC0416 | 1.145552 | 3-oxoacyl-ACP reductase |
| XCC0417 | 4.558873 | pyridine nucleotide-disulfide oxidoreductase |
| XCC0418 | 4.022474 | VirK protein |
| XCC0431 | 1.25914 | nuclease |
| XCC0439 | 1.050778 | lectin |
| XCC0449 | 1.42951 | lytic transglycosylase |
| XCC0450 | 2.185108 | putative exported protein |
| XCC0451 | 1.284961 | lipoprotein |
| XCC0456 | 1.160004 | membrane protein |
| XCC0458 | 1.528525 | diacylglycerol kinase-like enzyme |
| XCC0491 | -1.51568 | membrane protein |
| XCC0492 | -1.31069 | membrane protein |
| XCC0507 | 2.757377 | ice nucleation protein |
| XCC0521 | 1.091898 | ribonuclease |
| XCC0535 | 2.924554 | IS5/IS1182 family transposase |
| XCC0539 | 2.179382 | membrane protein |
| XCC0546 | 4.742417 | secreted protein |
| XCC0556 | 1.32432 | chorismate mutase |
| XCC0570 | 2.924554 | IS5/IS1182 family transposase |
| XCC0571 | -2.30686 | hypothetical protein |
| XCC0572 | -4.12022 | Putative secreted protein |
| XCC0575 | 1.551612 | lactoylglutathione lyase |
| XCC0584 | 1.735238 | phosphoglycolate phosphatase, bacterial |
| XCC0593 | -1.71736 | twitching motility protein PilT |
| XCC0625 | 1.039593 | xanthan biosynthesis protein XanB |
| XCC0626 | 1.289577 | phosphoglucomutase |
| XCC0638 | 2.924554 | IS5/IS1182 family transposase |
| XCC0644 | 4.103166 | pectate lyase |
| XCC0645 | 2.315938 | pectate lyase |
| XCC0659 | 2.076844 | protease |
| XCC0678 | 1.253869 | glycosyl transferase |
| XCC0679 | 1.431125 | membrane protein |
| XCC0689 | 1.137538 | putative membrane protein |
| XCC0694 | -2.44179 | putative exported protein |
| XCC0695 | -1.82062 | riboflavin synthase |
| XCC0696 | -1.07811 | 3,4-dihydroxy-2-butanone-4-phosphate synthase |
| XCC0742 | 1.44716 | NAD(P)H quinone oxidoreductase |
| XCC0748 | 2.830386 | alpha-amylase |
| XCC0758 | 1.272107 | membrane protein |
| XCC0767 | 1.44423 | hemin transporter HemP |
| XCC0768 | 1.009523 | sugar transporter |
| XCC0771 | 1.46223 | transmembrane signal peptide protein |
| XCC0772 | 3.571906 | permease |
| XCC0773 | 1.876286 | ABC transporter ATP-binding protein |
| XCC0774 | 1.972389 | nitrate ABC transporter substrate-binding protein |
| XCC0779 | 1.28086 | DNA-binding response regulator |
| XCC0783 | 2.007901 | LemA family protein |
| XCC0784 | 1.389325 | glycine rich protein |
| XCC0785 | 1.153176 | membrane protein |
| XCC0804 | 2.023847 | protocatechuate 4,5-dioxygenase subunit beta |
| XCC0805 | 1.509183 | protocatechuate 4,5-dioxygenase subunit alpha |
| XCC0806 | 1.917706 | transcriptional regulator |
| XCC0807 | -1.07072 | acyl-CoA synthetase |
| XCC0808 | -1.16148 | aldehyde dehydrogenase |
| XCC0809 | -1.05521 | aldehyde dehydrogenase |
| XCC0811 | -2.12856 | MarR family transcriptional regulator |
| XCC0812 | -3.80403 | hypothetical protein |
| XCC0815 | -1.01345 | glucose-fructose oxidoreductase |
| XCC0818 | 3.577185 | hypothetical protein |
| XCC0829 | 1.659801 | glutamine amidotransferase |
| XCC0843 | 4.126093 | alpha/beta hydrolase |
| XCC0845 | 1.883385 | conserved hypothetical protein |
| XCC0846 | 1.87277 | membrane protein |
| XCC0847 | 2.221967 | RNA polymerase sigma factor |
| XCC0851 | 2.983998 | protease |
| XCC0852 | 2.965097 | peptidase S8 |
| XCC0854 | -3.8838 | peptidase S8 |
| XCC0863 | -5.89625 | putative membrane protein |
| XCC0864 | 1.579633 | transcriptional regulator |
| XCC0923 | 2.580217 | membrane protein |
| XCC0930 | 3.082511 | cell wall hydrolase |
| XCC0931 | 2.277741 | hypothetical protein |
| XCC0940 | 1.235471 | sulfate transporter subunit |
| XCC0943 | 1.012003 | sulfate/thiosulfate import ATP-binding protein CysA |
| XCC0944 | 1.188557 | Putative secreted protein |
| XCC0997 | 1.027215 | cyclic pyranopterin monophosphate synthase MoaC |
| XCC1029 | 3.040766 | lipoprotein |
| XCC1030 | 1.942022 | propionate catabolism regulatory protein |
| XCC1031 | -5.8147 | methylisocitrate lyase |
| XCC1032 | -4.22549 | 2-methylcitrate synthase |
| XCC1033 | -4.05481 | Fe/S-dependent 2-methylisocitrate dehydratase AcnD |
| XCC1034 | -1.38399 | addiction module antitoxin |
| XCC1035 | -1.66781 | plasmid stabilization protein |
| XCC1036 | -1.12033 | 3-methylitaconate isomerase |
| XCC1045 | 3.241182 | bacterioferritin |
| XCC1046 | 3.077886 | peroxiredoxin |
| XCC1047 | 1.4763 | heat-shock protein |
| XCC1072 | 2.03778 | type III secretion system effector protein |
| XCC1073 | 1.531428 | sugar phosphate isomerases/epimerases |
| XCC1075 | 3.424142 | histidine phosphatase family protein |
| XCC1076 | 2.864095 | ADP-dependent (S)-NAD(P)H-hydrate dehydratase |
| XCC1077 | -1.39468 | glycosyl hydrolase |
| XCC1078 | 2.021311 | glycosyl hydrolase |
| XCC1079 | 2.828529 | NAD(P)-dependent oxidoreductase |
| XCC1082 | -1.6503 | short-chain dehydrogenase |
| XCC1086 | -2.95236 | EAL domain-containing protein |
| XCC1088 | 1.046307 | excinuclease ABC subunit A |
| XCC1089 | 1.50789 | transducer protein car |
| XCC1091 | 3.40337 | aldehyde oxidase |
| XCC1092 | 3.07092 | FAD-binding molybdopterin dehydrogenase |
| XCC1093 | 2.664972 | ferredoxin |
| XCC1097 | 4.87948 | DNA-binding protein |
| XCC1102 | 1.105111 | hypothetical protein |
| XCC1107 | -1.22546 | putative secreted protein |
| XCC1108 | -1.20547 | AraC family transcriptional regulator |
| XCC1109 | 2.411119 | catalase HPII |
| XCC1115 | 1.039788 | hypothetical protein |
| XCC1117 | -1.02402 | phosphoethanolamine transferase |
| XCC1127 | -1.07555 | two-component sensor histidine kinase |
| XCC1142 | 4.701305 | calcium-binding protein |
| XCC1159 | -1.44089 | cytochrome ubiquinol oxidase subunit II |
| XCC1160 | -1.10729 | cytochrome ubiquinol oxidase subunit I |
| XCC1161 | -1.23181 | cytochrome o ubiquinol oxidase subunit III |
| XCC1162 | -1.37692 | cytochrome o ubiquinol oxidase subunit IV |
| XCC1171 | -1.34833 | polyvinylalcohol dehydrogenase |
| XCC1172 | -1.72829 | anti-sigma factor antagonist |
| XCC1173 | -1.13692 | anti-sigma regulatory factor |
| XCC1174 | -1.43495 | transcriptional regulator |
| XCC1175 | -1.38549 | two-component system sensor protein |
| XCC1176 | -1.16899 | hybrid sensor histidine kinase/response regulator |
| XCC1178 | -1.18603 | glycoside hydrolase 43 family protein |
| XCC1182 | -1.03544 | hybrid sensor histidine kinase/response regulator |
| XCC1208 | -1.78147 | lactoylglutathione lyase |
| XCC1209 | 2.924554 | IS5/IS1182 family transposase |
| XCC1217 | 2.199483 | HPr kinase |
| XCC1218 | 1.306772 | type III secretion system effector protein |
| XCC1219 | 2.674232 | HrpW protein |
| XCC1220 | 2.425776 | HpaB protein |
| XCC1221 | 2.501905 | HPr kinase |
| XCC1222 | 2.382479 | HPr kinase |
| XCC1223 | 2.315324 | HPr kinase |
| XCC1224 | 2.23146 | HpaA protein |
| XCC1226 | 1.510833 | type III secretion system protein |
| XCC1227 | 1.4668 | HrcQ protein |
| XCC1228 | 1.762838 | type III secretion protein |
| XCC1229 | 1.430443 | hypersensitivity response secretion protein hrcV |
| XCC1230 | 1.219909 | EscU/YscU/HrcU family type III secretion system export apparatus switch protein |
| XCC1231 | 2.350707 | HPr kinase |
| XCC1232 | 2.123681 | type III secretion protein HrpB2 |
| XCC1233 | 2.282155 | EscJ/YscJ/HrcJ family type III secretion inner membrane ring protein |
| XCC1234 | 1.992489 | type III secretion protein HrpB4 |
| XCC1235 | 3.015891 | type III secretion system protein |
| XCC1236 | 1.936587 | EscN/YscN/HrcN family type III secretion system ATPase |
| XCC1238 | 1.353542 | EscT/YscT/HrcT family type III secretion system export apparatus protein |
| XCC1239 | 2.416299 | EscC/YscC/HrcC family type III secretion system outer membrane ring protein |
| XCC1240 | 3.335296 | Hpa1 protein |
| XCC1241 | 3.482891 | lytic transglycosylase |
| XCC1244 | 1.820379 | hypothetical protein |
| XCC1245 | 2.310262 | transposase |
| XCC1246 | 2.327891 | type III effector |
| XCC1249 | 3.016302 | hypothetical protein |
| XCC1252 | 1.440042 | hypothetical protein |
| XCC1255 | 2.924554 | IS5/IS1182 family transposase |
| XCC1256 | 1.119981 | beta-galactosidase |
| XCC1260 | 1.01978 | methylmalonate-semialdehyde dehydrogenase (CoA acylating) |
| XCC1261 | 1.057067 | acyl-CoA dehydrogenase |
| XCC1262 | 1.143399 | enoyl-CoA hydratase |
| XCC1272 | -1.41859 | DUF4845 domain-containing protein |
| XCC1278 | 1.092752 | hypothetical protein |
| XCC1289 | 2.486278 | DNA ligase-associated DEXH box helicase |
| XCC1290 | 2.040358 | ATP-dependent DNA ligase |
| XCC1291 | 1.55883 | DNA ligase-associated DEXH box helicase |
| XCC1294 | 1.999201 | GGDEF domain-containing protein |
| XCC1295 | 2.271482 | membrane protein |
| XCC1298 | -1.42387 | serine protease |
| XCC1308 | 1.640644 | putative exported protein |
| XCC1310 | 1.159045 | peptidyl-prolyl cis-trans isomerase |
| XCC1311 | 4.91607 | cation transporter |
| XCC1313 | -1.16749 | hypothetical protein |
| XCC1315 | 2.399385 | alkene reductase |
| XCC1318 | 4.666027 | hypothetical protein |
| XCC1332 | 1.356574 | delta 9 acyl-lipid fatty acid desaturase |
| XCC1333 | 1.126028 | hypothetical protein |
| XCC1334 | 1.17323 | RNA polymerase sigma factor |
| XCC1335 | 1.885477 | membrane protein |
| XCC1340 | 5.508024 | TonB-dependent receptor |
| XCC1343 | 2.042091 | S-methylmethionine permease |
| XCC1344 | 1.417895 | homocysteine S-methyltransferase |
| XCC1350 | -3.14322 | hypothetical protein |
| XCC1372 | -1.54484 | GGDEF domain-containing protein, partial |
| XCC1373 | -1.02855 | GGDEF domain-containing protein |
| XCC1376 | -3.84628 | pilus assembly protein |
| XCC1377 | -14.6433 | putative exported protein |
| XCC1378 | -2.07409 | spore coat protein U |
| XCC1379 | -2.16102 | outer membrane usher protein FasD |
| XCC1380 | -2.85031 | pilus assembly protein |
| XCC1381 | -4.39263 | protein U |
| XCC1390 | 2.399293 | Nitrous oxidase accessory protein |
| XCC1408 | -8.84683 | hypothetical protein |
| XCC1409 | 1.567375 | N-acetyltransferase |
| XCC1410 | 1.174819 | DUF3298 domain-containing protein |
| XCC1420 | 2.338783 | phospholipase |
| XCC1423 | 1.796875 | peptidoglycan-associated outer membrane lipoprotein |
| XCC1433 | 3.105616 | NADPH-dependent oxidoreductase |
| XCC1435 | 1.60605 | deoxyribodipyrimidine photo-lyase |
| XCC1436 | 1.610112 | cell envelope biogenesis protein OmpA |
| XCC1438 | -2.40038 | aklaviketone reductase |
| XCC1439 | -2.4681 | MexE family multidrug efflux RND transporter periplasmic adaptor subunit |
| XCC1440 | -2.26125 | multidrug efflux RND transporter permease subunit |
| XCC1441 | -1.96406 | short-chain dehydrogenase |
| XCC1442 | -2.24116 | RND transporter |
| XCC1443 | -4.28699 | GGDEF domain-containing protein |
| XCC1444 | 3.200025 | hypothetical protein |
| XCC1445 | -5.37035 | sensor histidine kinase |
| XCC1447 | -11.8842 | hypothetical protein |
| XCC1454 | 2.924554 | IS5/IS1182 family transposase |
| XCC1506 | 1.217043 | hypothetical protein |
| XCC1530 | 1.49868 | carbonic anhydrase |
| XCC1531 | 1.332165 | sulfate transporter |
| XCC1543 | 1.058923 | SAM-dependent methyltransferase |
| XCC1575 | 3.280563 | membrane-bound PQQ-dependent dehydrogenase, glucose/quinate/shikimate family |
| XCC1576 | -15.1882 | secreted protein |
| XCC1577 | -2.22018 | urocanate hydratase |
| XCC1578 | -2.1274 | N-formylglutamate deformylase |
| XCC1579 | -1.82035 | histidine ammonia-lyase |
| XCC1580 | -1.05996 | formimidoylglutamate deiminase |
| XCC1581 | -1.09086 | imidazolonepropionase |
| XCC1584 | 1.588547 | poly(hydroxyalcanoate) granule associated protein |
| XCC1592 | 1.711234 | cell envelope biogenesis protein TonB |
| XCC1616 | 4.582308 | hypothetical protein |
| XCC1617 | 4.661217 | hypothetical protein |
| XCC1634 | 9.194757 | ISxac3 transposase |
| XCC1636 | 5.089957 | IS5/IS1182 family transposase |
| XCC1638 | 2.924554 | IS5/IS1182 family transposase |
| XCC1653 | 2.575788 | response regulator |
| XCC1654 | -1.1363 | circadian clock protein KaiC |
| XCC1662 | -1.86974 | heme ABC transporter permease |
| XCC1665 | -2.31094 | DNA-directed RNA polymerase sigma-70 factor |
| XCC1687 | 1.709804 | general stress protein |
| XCC1692 | -1.06249 | transmembrane |
| XCC1727 | -4.81701 | chemotaxis protein |
| XCC1743 | -1.02793 | ferredoxin |
| XCC1744 | 1.085782 | MFS transporter |
| XCC1745 | 1.296819 | regucalcin |
| XCC1750 | -1.94439 | TonB-dependent receptor, partial |
| XCC1751 | -2.41303 | TonB-dependent receptor, partial |
| XCC1752 | -1.94262 | cellulase |
| XCC1774 | 1.708302 | alkaline phosphatase |
| XCC1776 | -1.10471 | sodium transporter |
| XCC1777 | -6.57687 | sensor domain-containing phosphodiesterase |
| XCC1779 | -1.43399 | DNA-binding response regulator |
| XCC1780 | -1.55816 | two-component sensor histidine kinase |
| XCC1797 | 2.924554 | IS5/IS1182 family transposase |
| XCC1837 | -1.28398 | hypothetical protein |
| XCC1849 | -2.95191 | membrane protein |
| XCC1858 | -1.67798 | long-chain fatty acid--CoA ligase |
| XCC1859 | -1.33862 | hypothetical protein |
| XCC1860 | 1.889141 | aconitate hydratase |
| XCC1861 | 1.206574 | AbrB family transcriptional regulator |
| XCC1862 | 1.500191 | DNA-binding protein |
| XCC1863 | 1.474364 | aconitate hydratase B |
| XCC1865 | -3.9234 | bifunctional diguanylate cyclase/phosphodiesterase |
| XCC1866 | -4.38409 | chemotaxis response regulator protein-glutamate methylesterase |
| XCC1867 | -3.61851 | chemoreceptor glutamine deamidase CheD |
| XCC1868 | -8.41983 | chemotaxis protein CheR |
| XCC1869 | -10.2712 | methyl-accepting chemotaxis protein |
| XCC1870 | -8.95996 | putative secreted protein |
| XCC1871 | -8.62717 | chemotaxis protein |
| XCC1872 | -10.5555 | pilus assembly protein PilZ |
| XCC1873 | -6.41504 | chemotaxis protein |
| XCC1874 | -6.72086 | methyl-accepting chemotaxis protein |
| XCC1875 | -1.80091 | methyl-accepting chemotaxis protein |
| XCC1876 | -5.94125 | chemotaxis protein |
| XCC1877 | -13.6073 | methyl-accepting chemotaxis protein |
| XCC1878 | -9.04061 | methyl-accepting chemotaxis protein |
| XCC1879 | -8.0773 | methyl-accepting chemotaxis protein |
| XCC1880 | -5.39308 | chemotaxis protein |
| XCC1881 | -5.89692 | chemotaxis protein |
| XCC1882 | -8.36401 | chemotaxis protein, partial |
| XCC1883 | -15.683 | lipoprotein |
| XCC1885 | -6.6164 | chemotaxis protein CheA |
| XCC1886 | -6.16651 | chemotaxis response regulator |
| XCC1887 | -8.12972 | anti-sigma factor antagonist-like protein |
| XCC1888 | -8.36055 | chemotaxis protein |
| XCC1889 | -7.86062 | chromosome partioning protein |
| XCC1890 | -10.0994 | flagellar motor protein MotD |
| XCC1891 | -11.8118 | flagellar motor protein |
| XCC1892 | -1.78035 | TonB-dependent receptor |
| XCC1903 | -7.42999 | chemotaxis protein CheA |
| XCC1904 | -6.30431 | chemotaxis protein |
| XCC1905 | -6.58213 | chemotaxis protein |
| XCC1906 | -8.09543 | RNA polymerase sigma factor |
| XCC1907 | -8.21681 | cobyrinic acid a,c-diamide synthase |
| XCC1908 | -10.1308 | flagellar biosynthesis regulator FlhF |
| XCC1909 | -5.64557 | flagellar biosynthesis protein FlhA |
| XCC1910 | -3.41157 | flagellar biosynthesis protein FlhB |
| XCC1911 | -4.61548 | bifunctional diguanylate cyclase/phosphodiesterase |
| XCC1912 | -4.47045 | GGDEF domain-containing protein |
| XCC1913 | -3.551 | GGDEF domain-containing protein |
| XCC1914 | -5.58496 | flagellar biosynthetic protein FliR |
| XCC1915 | -4.39725 | flagellar biosynthesis |
| XCC1916 | -7.48074 | flagellar biosynthetic protein FliP |
| XCC1917 | -6.15446 | flagellar protein |
| XCC1918 | -4.00511 | flagellar motor switch protein FliN |
| XCC1919 | -6.43611 | flagellar motor switch protein FliM |
| XCC1920 | -5.07915 | flagellar biosynthesis protein |
| XCC1921 | -3.77892 | flagellar protein |
| XCC1922 | -3.52109 | flagellar export protein FliJ |
| XCC1923 | -3.97522 | flagellar protein export ATPase FliI |
| XCC1924 | -5.03649 | flagellar assembly protein FliH |
| XCC1925 | -3.98834 | flagellar motor switch protein FliG |
| XCC1926 | -2.93639 | flagellar M-ring protein FliF |
| XCC1927 | -4.91411 | flagellar hook-basal body complex protein FliE |
| XCC1935 | -1.32103 | RNA polymerase sigma-54 factor |
| XCC1937 | -5.61263 | PilZ domain-containing protein |
| XCC1938 | -5.15134 | hypothetical protein |
| XCC1939 | -6.79416 | flagellar protein FliS |
| XCC1940 | -9.09627 | flagellar protein |
| XCC1941 | -8.91223 | flagellin |
| XCC1942 | -5.60668 | flagellar hook-associated protein FlgL |
| XCC1943 | -5.98773 | flagellar hook-associated protein FlgK |
| XCC1944 | -6.35867 | flagellar rod assembly protein FlgJ |
| XCC1945 | -6.63528 | flagellar P-ring protein |
| XCC1946 | -7.68816 | flagellar L-ring protein |
| XCC1947 | -7.99938 | flagellar basal body rod protein FlgG |
| XCC1948 | -5.86346 | flagellar basal body rod protein FlgF |
| XCC1949 | -6.6917 | flagellar hook protein FlgE |
| XCC1950 | -6.27601 | flagellar basal body rod modification protein FlgD |
| XCC1951 | -6.77755 | flagellar basal body rod protein FlgC |
| XCC1952 | -8.05965 | flagellar biosynthesis protein FlgB |
| XCC1953 | -7.30801 | chemotaxis protein |
| XCC1954 | -5.21475 | flagellar basal body P-ring biosynthesis protein FlgA |
| XCC1955 | -6.54857 | flagellar biosynthesis anti-sigma factor FlgM |
| XCC1956 | -5.63298 | flagella protein |
| XCC1959 | -6.91171 | GGDEF domain-containing protein |
| XCC1961 | -3.17625 | hypothetical protein |
| XCC1962 | -6.69264 | chemotaxis protein |
| XCC1991 | 3.727537 | hypothetical protein |
| XCC1992 | 2.112367 | short-chain dehydrogenase |
| XCC1994 | 1.970725 | hypothetical protein |
| XCC1997 | 1.298523 | outer membrane or secreted lipoprotein |
| XCC2001 | -1.28021 | molybdenum cofactor guanylyltransferase |
| XCC2012 | -2.2109 | NAD(P)-dependent oxidoreductase |
| XCC2013 | -3.13804 | dTDP-4-dehydrorhamnose 3,5-epimerase |
| XCC2014 | -2.21178 | NAD-dependent epimerase |
| XCC2015 | -3.62189 | glucose-1-phosphate cytidylyltransferase |
| XCC2021 | -1.67979 | chemotaxis protein-glutamate methylesterase |
| XCC2023 | -3.10103 | diguanylate phosphodiesterase |
| XCC2024 | -9.03937 | YapH protein |
| XCC2025 | -3.18068 | serine protease |
| XCC2031 | 2.87606 | membrane protein |
| XCC2032 | 2.80458 | sorbosone dehydrogenase |
| XCC2034 | -1.23638 | succinoglycan biosynthesis protein |
| XCC2037 | -1.14512 | putative exported protein |
| XCC2038 | -1.27686 | SCO family protein |
| XCC2047 | -2.69617 | methyl-accepting chemotaxis protein |
| XCC2050 | 9.78136 | alginate export family protein |
| XCC2053 | 1.848409 | response regulator |
| XCC2057 | -3.42836 | phage-related protein |
| XCC2059 | -2.21085 | single-stranded DNA binding protein |
| XCC2074 | -2.21085 | single-stranded DNA binding protein |
| XCC2099 | 1.554941 | avirulence protein |
| XCC2105 | 2.924554 | IS5/IS1182 family transposase |
| XCC2124 | 1.927983 | NAD dependent epimerase/dehydratase |
| XCC2142 | -1.03325 | transporter |
| XCC2143 | -1.3253 | MexH family multidrug efflux RND transporter periplasmic adaptor subunit |
| XCC2150 | 3.506634 | hypothetical protein |
| XCC2155 | 4.030613 | hypothetical protein |
| XCC2156 | 4.198215 | oxidoreductase |
| XCC2181 | -4.36811 | HAMP domain-containing protein |
| XCC2182 | -2.71597 | hypothetical protein |
| XCC2188 | 1.002328 | UTP--glucose-1-phosphate uridylyltransferase |
| XCC2207 | -1.01809 | lipoprotein, putative |
| XCC2208 | 1.233027 | membrane protein |
| XCC2215 | 2.118807 | putative exported protein |
| XCC2262 | 2.99243 | hypothetical protein |
| XCC2263 | 2.153304 | MBL fold metallo-hydrolase |
| XCC2264 | 3.245968 | general stress protein |
| XCC2266 | 1.095847 | polygalacturonase |
| XCC2279 | 1.377312 | ribonuclease |
| XCC2284 | 1.000277 | SELO family protein |
| XCC2286 | 1.477233 | glutathione S-transferase |
| XCC2287 | -1.18748 | cold-shock protein |
| XCC2296 | -1.05248 | TraB/GumN family protein |
| XCC2297 | -1.79899 | hypothetical protein |
| XCC2304 | 2.661181 | virulence factor |
| XCC2307 | 1.476544 | ATP-dependent DNA ligase |
| XCC2308 | 3.111302 | DUF3606 domain-containing protein |
| XCC2312 | 2.818682 | RecA/RadA recombinase |
| XCC2314 | -6.57441 | chemotaxis protein CheW |
| XCC2315 | -8.23089 | methyl-accepting chemotaxis protein |
| XCC2325 | -1.08427 | murein transglycosylase |
| XCC2336 | 3.300973 | succinate-semialdehyde dehydrogenase |
| XCC2341 | 1.239766 | membrane protein |
| XCC2342 | 1.06658 | MFS transporter |
| XCC2343 | 1.404308 | transporter |
| XCC2350 | -6.46975 | diguanylate cyclase |
| XCC2353 | -2.96408 | hypothetical protein |
| XCC2354 | -1.97812 | formate dehydrogenase a chain |
| XCC2355 | -2.81968 | sulfurtransferase FdhD |
| XCC2356 | -3.42014 | MFS transporter |
| XCC2372 | 1.315904 | PTS fructose EIIBC component |
| XCC2373 | 1.354515 | carbohydrate porin |
| XCC2374 | 1.089965 | lipase |
| XCC2396 | 1.026103 | avirulence protein |
| XCC2403 | -5.89008 | hypothetical protein |
| XCC2420 | 1.261795 | hypothetical protein |
| XCC2443 | 1.677178 | GumM protein |
| XCC2444 | 1.903532 | GumL protein |
| XCC2445 | 1.767406 | UDP-glucuronate--glycolipid 2-beta-glucuronosyltransferase |
| XCC2446 | 1.65097 | lipopolysaccharide biosynthesis protein |
| XCC2447 | 1.775003 | GDP-mannose--glycolipid 4-beta-D-mannosyltransferase |
| XCC2448 | 1.451413 | glycosyl transferase family 1 |
| XCC2449 | 1.244393 | GumG protein |
| XCC2450 | 1.112153 | GumF protein |
| XCC2451 | 1.763335 | gumE protein |
| XCC2452 | 2.423243 | GumD protein |
| XCC2453 | 1.037851 | GumC protein |
| XCC2490 | 2.924554 | IS5/IS1182 family transposase |
| XCC2497 | -1.35079 | Oar protein |
| XCC2506 | 1.767339 | hypothetical protein |
| XCC2534 | -2.22686 | cyanoglobin |
| XCC2535 | -1.35674 | hypothetical protein |
| XCC2536 | -2.00889 | phosphodiesterase |
| XCC2537 | -1.46535 | methylamine utilization protein |
| XCC2547 | -1.10119 | glyoxalase |
| XCC2560 | -1.27701 | membrane protein |
| XCC2561 | -1.71411 | membrane protein |
| XCC2565 | 1.902275 | leucin rich protein |
| XCC2566 | 1.352619 | carboxymethylenebutenolidase |
| XCC2567 | 1.019575 | HIT family protein |
| XCC2574 | -1.08413 | peptidase |
| XCC2575 | 4.942648 | peptidase |
| XCC2593 | 1.10767 | acyl-CoA thioesterase |
| XCC2601 | 2.48586 | peptidase |
| XCC2602 | 2.226973 | peptidase M35 |
| XCC2603 | -1.51489 | membrane protein |
| XCC2624 | 2.305422 | conserved membrane protein |
| XCC2630 | 2.775053 | hypothetical protein |
| XCC2639 | -1.2094 | membrane protein |
| XCC2641 | -3.82424 | GGDEF domain-containing protein |
| XCC2664 | 3.481035 | formate dehydrogenase subunit alpha |
| XCC2665 | 1.555806 | TonB-dependent receptor |
| XCC2666 | 4.570772 | serine protease |
| XCC2667 | 4.501413 | serine protease |
| XCC2668 | 2.942078 | hypothetical protein |
| XCC2669 | 3.196999 | serine protease |
| XCC2680 | 2.23654 | transcriptional regulator |
| XCC2681 | 2.289225 | multidrug transporter |
| XCC2682 | 3.452902 | multidrug efflux RND transporter permease subunit |
| XCC2683 | 4.23081 | MexE family multidrug efflux RND transporter periplasmic adaptor subunit |
| XCC2699 | -8.69278 | anti-anti-sigma factor |
| XCC2700 | -9.67752 | chemotaxis protein CheA |
| XCC2701 | -7.37984 | methyl-accepting chemotaxis protein |
| XCC2702 | -14.8032 | chemotaxis protein CheW |
| XCC2703 | -5.85924 | transcriptional regulator |
| XCC2704 | -6.30367 | SAM-dependent methyltransferase |
| XCC2705 | -4.30504 | chemotaxis response regulator protein-glutamate methylesterase |
| XCC2713 | 3.503712 | endonuclease V |
| XCC2715 | -1.32879 | Putative secreted protein |
| XCC2721 | 1.259011 | ABC transporter permease |
| XCC2722 | 1.480416 | phospholipase A(1) |
| XCC2724 | 12.67176 | hypothetical protein |
| XCC2725 | 1.538693 | cytidylyltransferase-like enzyme |
| XCC2729 | 1.249786 | aldehyde dehydrogenase iron-sulfur subunit |
| XCC2730 | 1.890983 | NAD(P)-dependent alcohol dehydrogenase |
| XCC2731 | -1.0022 | transcriptional regulator |
| XCC2737 | 1.181823 | dienelactone hydrolase |
| XCC2745 | 3.571332 | peroxiredoxin |
| XCC2752 | 3.47478 | DUF72 protein |
| XCC2754 | -1.23718 | twitching motility protein |
| XCC2755 | -1.50563 | twitching motility protein |
| XCC2759 | 1.268534 | peptidase |
| XCC2763 | 2.13245 | glutamine amidotransferase |
| XCC2781 | 1.985532 | competence protein ComEA |
| XCC2812 | -3.3973 | cytochrome D ubiquinol oxidase subunit II |
| XCC2813 | -3.21685 | cytochrome ubiquinol oxidase subunit I |
| XCC2815 | 1.07304 | pectate lyase |
| XCC2818 | 1.586026 | LuxR family transcriptional regulator |
| XCC2820 | 2.488254 | endoproteinase ArgC, partial |
| XCC2821 | 2.413142 | serine protease |
| XCC2828 | -1.66387 | TonB-dependent receptor |
| XCC2854 | 2.033366 | hydrolase |
| XCC2861 | 3.516118 | membrane protein |
| XCC2867 | -2.50413 | TonB-dependent receptor |
| XCC2878 | -2.02379 | membrane protein |
| XCC2887 | -1.37867 | TonB-dependent receptor |
| XCC2888 | -1.05784 | alpha-L-fucosidase |
| XCC2896 | 1.924449 | virulence factor |
| XCC2899 | 1.895161 | type III secretion system effector protein |
| XCC2907 | -4.5536 | MULTISPECIES: hypothetical protein |
| XCC2908 | -6.13315 | RebB protein |
| XCC2910 | -1.0298 | hypothetical protein |
| XCC2915 | 4.287264 | VWA domain-containing protein |
| XCC2918 | -3.14048 | alanine acetyltransferase |
| XCC2919 | -2.06149 | chemotaxis protein CheW |
| XCC2920 | -1.63281 | chemotaxis protein |
| XCC2921 | -2.31363 | two-component system sensor histidine kinase/response regulator |
| XCC2922 | -2.27817 | pilus biogenesis protein |
| XCC2923 | -2.59784 | pilus biogenesis protein |
| XCC2924 | -2.96994 | response regulator |
| XCC2925 | -1.93799 | response regulator |
| XCC2937 | 1.075628 | pyrroloquinoline quinone biosynthesis protein PqqB |
| XCC2940 | 1.777449 | coenzyme PQQ synthesis protein E |
| XCC2943 | -5.47284 | glucokinase |
| XCC2944 | -5.51778 | TonB-dependent receptor |
| XCC2949 | 1.092827 | response regulator |
| XCC2957 | 1.453718 | lipase |
| XCC2958 | 1.840628 | response regulator |
| XCC2959 | 1.041122 | histidine kinase |
| XCC3031 | 1.022617 | lipoprotein |
| XCC3034 | 1.072604 | MFS transporter |
| XCC3035 | 1.55709 | alpha,alpha-trehalose-phosphate synthase |
| XCC3043 | 1.550807 | ligand-gated channel |
| XCC3044 | 3.405579 | glyoxalase |
| XCC3045 | 1.947297 | ligand-gated channel |
| XCC3046 | 2.559762 | ferric enterobactin receptor |
| XCC3049 | 2.376292 | 4-hydroxy-2-oxovalerate aldolase |
| XCC3050 | 2.643878 | citrate-dependent iron transporter |
| XCC3051 | 3.911814 | carboxylate--amine ligase |
| XCC3052 | 3.91427 | IucA/IucC family siderophore biosynthesis protein |
| XCC3054 | 3.396587 | iron transporter |
| XCC3055 | 4.253847 | diaminopimelate decarboxylase |
| XCC3068 | 1.68278 | O-acetylhomoserine aminocarboxypropyltransferase |
| XCC3073 | 1.335072 | glutathione S-transferase |
| XCC3080 | 2.372051 | trehalose-phosphatase |
| XCC3081 | 2.792983 | glucoamylase |
| XCC3082 | 2.98016 | trehalose-6-phosphate synthase |
| XCC3083 | 2.215385 | membrane-bound PQQ-dependent dehydrogenase, glucose/quinate/shikimate family |
| XCC3084 | -5.49846 | chemotaxis protein |
| XCC3101 | -1.00189 | type 4 prepilin-like proteins leader peptide-processing enzyme |
| XCC3102 | -1.39677 | dephospho-CoA kinase |
| XCC3109 | 4.22879 | glycogen debranching enzyme |
| XCC3113 | 9.194757 | ISxac3 transposase |
| XCC3122 | 2.924554 | IS5/IS1182 family transposase |
| XCC3167 | -1.42223 | N-acetyltransferase |
| XCC3171 | 1.922463 | adenylyl-sulfate kinase |
| XCC3172 | 1.941383 | sulfate adenylyltransferase subunit 2 |
| XCC3173 | 1.089122 | sulfite reductase subunit alpha |
| XCC3174 | 1.719022 | sulfite reductase subunit beta |
| XCC3175 | 1.903718 | phosphoadenosine phosphosulfate reductase |
| XCC3176 | -2.07757 | peptidase |
| XCC3177 | -1.67392 | TonB-dependent receptor |
| XCC3181 | 1.931828 | uroporphyrinogen-III C-methyltransferase |
| XCC3182 | 2.352159 | cysteine synthase A |
| XCC3184 | 2.875597 | O-acetyl-ADP-ribose deacetylase |
| XCC3196 | -1.87192 | IS5/IS1182 family transposase |
| XCC3210 | 3.761232 | peptidoglycan-binding protein |
| XCC3211 | 3.406424 | exported lysostaphin-like metalloendopeptidase |
| XCC3214 | 2.924554 | IS5/IS1182 family transposase |
| XCC3223 | 1.37598 | membrane protein |
| XCC3228 | -1.86057 | fimbrial protein |
| XCC3229 | -1.82629 | fimbrial assembly protein |
| XCC3230 | -1.99865 | fimbrial protein |
| XCC3231 | -1.93611 | fimbrial protein |
| XCC3232 | -1.98956 | fimbrial assembly protein |
| XCC3284 | 2.532322 | hypothetical protein |
| XCC3288 | 3.273211 | membrane protein |
| XCC3311 | 2.302457 | putative exported protein |
| XCC3317 | 1.87696 | transcriptional regulator |
| XCC3321 | -1.61514 | chemotaxis protein |
| XCC3327 | -1.0796 | 2-isopropylmalate synthase |
| XCC3343 | 2.924554 | IS5/IS1182 family transposase |
| XCC3346 | -1.10806 | C4-dicarboxylate transporter |
| XCC3347 | -1.22851 | porin |
| XCC3353 | 1.449353 | porin |
| XCC3354 | 1.173658 | citrate transporter |
| XCC3355 | 1.537665 | 3-ketoacyl-ACP reductase |
| XCC3356 | -1.07445 | LacI family transcriptional regulator |
| XCC3360 | 2.830422 | dimethylallyltransferase |
| XCC3367 | -1.07603 | acetylxylan esterase |
| XCC3377 | 2.91893 | truncated rhamnogalacturonase B |
| XCC3378 | 2.893406 | truncated rhamnogalacturonase B |
| XCC3379 | 2.536844 | truncated rhamnogalacturonase B |
| XCC3380 | 1.125838 | cellulase |
| XCC3382 | -3.92668 | fatty acid-binding protein DegV |
| XCC3383 | 3.481261 | hypothetical protein |
| XCC3384 | -1.12829 | EamA family transporter |
| XCC3402 | 4.127691 | beta-aspartyl-peptidase |
| XCC3403 | 5.33033 | betaine-aldehyde dehydrogenase |
| XCC3404 | 5.324305 | choline dehydrogenase |
| XCC3408 | 1.960724 | TonB-dependent receptor |
| XCC3417 | 3.934112 | type II secretion system protein L |
| XCC3421 | 10.70563 | type II secretion system protein H |
| XCC3437 | 5.58189 | BON domain-containing protein |
| XCC3438 | 1.23209 | oxidoreductase |
| XCC3439 | 1.045169 | 6-phosphogluconate dehydrogenase |
| XCC3440 | 1.537645 | hypothetical protein |
| XCC3441 | 3.389819 | membrane protein |
| XCC3447 | 1.349348 | endonuclease |
| XCC3455 | 2.089579 | membrane protein |
| XCC3459 | 1.482766 | endopolygalacturonase |
| XCC3463 | -10.6573 | transposase |
| XCC3472 | -1.00487 | sigma-54-dependent Fis family transcriptional regulator |
| XCC3474 | -1.23362 | TonB-dependent receptor |
| XCC3475 | -3.04301 | alcohol dehydrogenase |
| XCC3476 | -3.50855 | surface antigen gene |
| XCC3477 | -2.63141 | hypothetical protein |
| XCC3478 | -2.25341 | MoxJ protein |
| XCC3479 | -1.54443 | methanol dehydrogenase |
| XCC3481 | 13.85282 | ISxac3 transposase |
| XCC3482 | -3.59201 | methanol dehydrogenase |
| XCC3486 | -1.01656 | GGDEF domain-containing protein |
| XCC3487 | 2.624491 | MFS transporter |
| XCC3508 | 1.143157 | outer membrane lipoprotein |
| XCC3512 | -1.42997 | DNA-binding response regulator |
| XCC3519 | -4.12832 | GGDEF domain-containing protein |
| XCC3521 | 1.007248 | endoglucanase |
| XCC3522 | -7.09381 | chemotaxis protein |
| XCC3523 | -8.02433 | sensor domain-containing diguanylate cyclase |
| XCC3527 | 2.154059 | endonuclease |
| XCC3534 | 6.116838 | 1,4-beta-cellobiosidase |
| XCC3535 | 5.974056 | glycosidase |
| XCC3536 | 2.680287 | hypothetical protein |
| XCC3546 | -3.1592 | GGDEF domain-containing protein |
| XCC3559 | 11.88035 | DUF4126 domain-containing protein |
| XCC3561 | 2.128802 | glutathione-dependent reductase |
| XCC3566 | -3.5759 | aldehyde-activating protein |
| XCC3586 | 2.924554 | IS5/IS1182 family transposase |
| XCC3589 | 1.772895 | hypothetical protein |
| XCC3590 | 1.822413 | hypothetical protein |
| XCC3591 | 3.053968 | gluconolactonase |
| XCC3592 | 2.51245 | hypothetical protein |
| XCC3600 | 1.147844 | exported type III secretion effector protein |
| XCC3606 | 1.201446 | polyisoprenoid-binding protein |
| XCC3622 | 1.542198 | secreted protein |
| XCC3623 | 1.683759 | lipase |
| XCC3624 | 6.049066 | arabinogalactan endo-1,4-beta-galactosidase |
| XCC3627 | 2.924554 | IS5/IS1182 family transposase |
| XCC3640 | 3.602566 | hypothetical protein |
| XCC3641 | 1.346332 | sorbosone dehydrogenase |
| XCC3642 | 3.534691 | hypothetical protein |
| XCC3643 | 2.454171 | hybrid sensor histidine kinase/response regulator |
| XCC3645 | 4.912001 | hypothetical protein |
| XCC3647 | -1.283 | alanine racemase |
| XCC3653 | -5.44869 | flagellar motor protein MotA |
| XCC3654 | -6.11942 | flagellar motor protein MotB |
| XCC3663 | 1.340568 | fasciclin |
| XCC3666 | 1.036188 | dioxygenase |
| XCC3668 | 2.638676 | tryptophan repressor binding protein |
| XCC3669 | 2.865459 | oxidoreductase |
| XCC3670 | 4.631256 | glycosyl transferase family 2 |
| XCC3677 | -1.30202 | sensor histidine kinase |
| XCC3678 | -1.03001 | D-amino-acid oxidase |
| XCC3682 | 1.097513 | protein YciF |
| XCC3685 | -8.05128 | hypothetical protein |
| XCC3693 | 3.041512 | hypothetical protein |
| XCC3694 | 1.169925 | membrane protein |
| XCC3695 | 1.297213 | oxidoreductase |
| XCC3702 | 3.837793 | hypothetical protein |
| XCC3725 | -1.65211 | LysR family transcriptional regulator |
| XCC3732 | -12.2848 | transposase |
| XCC3735 | 3.868971 | hypothetical protein |
| XCC3750 | -1.84129 | peptidoglycan-binding protein LysM |
| XCC3754 | -1.37445 | RDD family protein |
| XCC3783 | 1.72557 | putative exported protein |
| XCC3784 | 2.065135 | peptidoglycan-binding protein LysM |
| XCC3790 | -2.4399 | dehydrogenase |
| XCC3791 | -1.1801 | VOC family protein |
| XCC3792 | -1.31086 | RNA polymerase subunit sigma-24 |
| XCC3797 | 2.364391 | hypothetical protein |
| XCC3798 | 1.965936 | EF hand domain-containing protein |
| XCC3802 | 1.106312 | peptidoglycan-binding protein |
| XCC3804 | 1.284543 | hypothetical protein |
| XCC3810 | 1.82979 | hypothetical protein |
| XCC3811 | 2.657548 | hypothetical protein |
| XCC3819 | 2.251104 | ribonuclease BN |
| XCC3825 | -1.02028 | cytochrome oxidase assembly protein |
| XCC3826 | -1.03974 | Putative membrane protein |
| XCC3835 | -1.22855 | bifunctional proline dehydrogenase/L-glutamate gamma-semialdehyde dehydrogenase |
| XCC3848 | -2.51813 | putative exported protein |
| XCC3857 | -1.85531 | phosphomannomutase/phosphoglucomutase |
| XCC3870 | -3.07244 | hypothetical protein |
| XCC3871 | 1.129359 | kinase |
| XCC3875 | 1.907861 | membrane protein |
| XCC3884 | 1.525339 | Conserved hypothetical protein |
| XCC3885 | 1.210102 | alpha/beta hydrolase |
| XCC3886 | 2.046485 | hypothetical protein |
| XCC3887 | 2.958398 | hypothetical protein |
| XCC3888 | 3.33886 | hypothetical protein |
| XCC3889 | 3.542137 | lipoprotein, putative |
| XCC3890 | 3.598463 | epimerase |
| XCC3891 | -1.36295 | mercuric reductase |
| XCC3897 | 1.670483 | histidine biosynthesis protein HisIE |
| XCC3899 | 1.875884 | hypothetical protein |
| XCC3900 | 1.559474 | membrane protein |
| XCC3901 | 1.533245 | hypothetical protein |
| XCC3908 | -1.90264 | putative secreted protein |
| XCC3911 | -1.29551 | acriflavin resistance protein |
| XCC3912 | -1.06459 | ABC transporter ATP-binding protein |
| XCC3913 | -1.63107 | membrane protein |
| XCC3914 | -1.36541 | membrane protein |
| XCC3915 | -2.47055 | alpha/beta hydrolase |
| XCC3924 | 5.379339 | CsbD family protein |
| XCC3925 | 3.133989 | entericidin A |
| XCC3927 | -2.39205 | roadblock-LC7 domain family |
| XCC3928 | -2.34825 | secreted protein |
| XCC3929 | -2.31364 | hypothetical protein |
| XCC3930 | -1.29232 | GTPase |
| XCC3947 | -3.22752 | membrane protein |
| XCC3955 | 1.096829 | iron-uptake factor |
| XCC3963 | -2.06343 | TonB-dependent receptor |
| XCC3964 | -1.28949 | peptidase |
| XCC3965 | -1.61147 | cupin |
| XCC3966 | -1.33844 | tryptophan halogenase |
| XCC3967 | -2.11806 | cell envelope biogenesis protein TonB |
| XCC3981 | 3.981204 | lipoprotein |
| XCC3989 | 2.729989 | hypothetical protein |
| XCC3991 | 2.088339 | carbonic anhydrase |
| XCC3993 | 1.399701 | glucose-6-phosphate dehydrogenase |
| XCC4008 | -1.01162 | acyltransferase |
| XCC4036 | -1.26373 | CusA/CzcA family heavy metal efflux RND transporter |
| XCC4037 | -1.01925 | cation efflux system protein |
| XCC4040 | -1.0991 | putative membrane protein |
| XCC4042 | 2.314697 | alkaline phosphatase |
| XCC4044 | 4.095523 | transglycosylase |
| XCC4057 | -1.37645 | cation acetate symporter |
| XCC4058 | -1.65037 | membrane protein |
| XCC4059 | -2.57911 | putative exported protein |
| XCC4062 | -3.62026 | hypothetical protein |
| XCC4063 | -2.50905 | cytochrome c biogenesis protein |
| XCC4080 | 2.907256 | polyvinylalcohol dehydrogenase |
| XCC4089 | 1.093828 | GMP synthase |
| XCC4108 | -3.9906 | restriction endonuclease or methylase |
| XCC4111 | 1.758341 | histone |
| XCC4112 | -1.69209 | ring canal kelch-like protein |
| XCC4127 | 3.135311 | alcohol dehydrogenase |
| XCC4130 | -1.02581 | conserved hypothetical protein |
| XCC4144 | 1.552312 | sensor histidine kinase |
| XCC4157 | 1.10144 | epimerase |
| XCC4158 | 3.8571 | putative exported protein |
| XCC4162 | 2.109973 | TonB-dependent siderophore receptor |
| XCC4176 | 2.011302 | cardiolipin synthase B |
| XCC4182 | 1.472347 | hypothetical protein |
| XCC4189 | -1.63651 | pseudouridine synthase |
| XCC4192 | 2.924554 | IS5/IS1182 family transposase |
| XCC4195 | 2.235593 | lipoprotein |
| XCC4201 | -1.94112 | hemagglutinin |
| XCC4213 | 1.287622 | glutathione peroxidase |
| XCC4214 | 2.612741 | hypothetical protein |
| XCC4222 | -1.85549 | membrane protein |
| XCC4237 | -1.57998 | Oar protein |
